# Seven naphtho-*γ*-pyrones from the marine-derived fungus *Alternaria alternata*: structure elucidation and biological properties

**DOI:** 10.1186/2191-2858-2-6

**Published:** 2012-02-29

**Authors:** Mohamed Shaaban, Khaled A Shaaban, Mohamed S Abdel-Aziz

**Affiliations:** 1 Chemistry of Natural Compounds Department, Pharmaceutical Industries Division, National Research Centre, El-Behoos St., Dokki-Cairo 12622, Egypt; 2 Institute of Organic and Biomolecular Chemistry, University of Göttingen, Tammannstrasse 2, D-37077 Göttingen, Germany; 3 Department of Microbial Chemistry, Genetic Engineering and Biotechnology Division, National Research Centre, El-Behoos St., Dokki-Cairo 12622, Egypt

**Keywords:** pyrone derivatives, *Alternaria alternata*, marine fungi, biological activity

## Abstract

Eight bioactive pyrone derivatives were identified from the culture of *Alternaria alternata *strain D2006, isolated from the marine soft coral *Denderonephthya hemprichi*, which was selected as its profound antimicrobial activities. The compounds were assigned as pyrophen (**1**), rubrofusarin B (**2**), fonsecin (**3**), and fonsecin B (**5**) beside to the four dimeric naphtho-γ-pyrones; aurasperone A (**6**), aurasperone B (**7**), aurasperone C (**8**), and aurasperone F (**9**). Structures of the isolated compounds were identified on the basis of 1D and 2D NMR spectroscopy and mass (EI, ESI, HRESI) data, and by comparison with the literature. Configuration of the four dimeric naphtho-γ-pyrones **6-9 **was analyzed by CD spectra, exhibiting an identical stereochemistry.

## 1. Background

Infectional diseases and drug resistance phenomena are the most effective reasons for the death of ca. 20 millions yearly. For example, tuberculosis (TB) was the leading cause of ca. two million deaths due to a bacterial pathogen, *Mycobacterium tuberculosis*, among them more than 80% of TB patients living in sub-Africa and Asia [1-4]. Thus, new and more-powerful drugs are necessary to solve these problems. Marine microorganisms, especially fungi, are still a less investigated resource of bioactive substances [5,6]; recent investigations indicated their tremendous potential as source of new drugs [7-13].

In this article, a report on the antimicrobial activity of naphtho-γ-pyrones (naphthopyran-4-ones) attracted our interest [14]. During the investigation of fungal strains for the production of structurally novel active compounds from marine microorganisms, we found that the EtOAc extract of the marine-derived fungal strain *Alternaria alternata *D2006 (isolated from a red soft coral, *Denderonephthya hemprichi*, collected from the Red Sea at Safaga coasts, Egypt) was selected due to its distinctive features in the chemical and biological assays. We therefore performed a bioassay-guided fractionation.

The crude extract possessed in the agar diffusion test potent activity against *Pseudomonas aeruginosa, Staphylococcus aureus *and *Candida albicans*. For isolation of the bioactive constituents, *A. alternata *D2006 was up-scaled as a shaker-culture using GYMP medium [15] (100% seawater) for 10 days. Thereafter, the obtained black broth was worked up [16] and separated by a series of chromatographic steps, yielding colourless semisolid of pyrophen (**1**) and seven naphtho-*γ*-pyrones (**2**, **3**, **5**-**9**) as yellow solids, among them four dimeric analogues (**6**-**9**). Herein, we describe their separation, structure elucidation (using 1D and 2D NMR and MS (EI, ESI, HRESI) data and antimicrobial properties.

## 2. Taxonomy and characterization

The fungal isolate was identified as *A. alternata *(Dematiaceae) according to Barnett [17]. Microscopically, the conidiophores were dark, simple, rather short or elongate and contained simple or branched chains of conidia. Conidia were dark, typically with both cross and longitudinal septa, with various shapes, obclavate to elliptical or ovoid. The fungal spores were multicellular, dark and having thick cell walls.

## 3. Results and discussion

The fungal extract showed several UV absorbing (254 nm) yellow bands, exhibiting yellowish-green UV fluorescence at 366 nm. On spraying with anisaldehyde/sulphuric acid and heating they turned orange to dark red, but showed no colour change with sodium hydroxide, thus excluding *peri*-hydroxyquinones.

**Figure 1:**
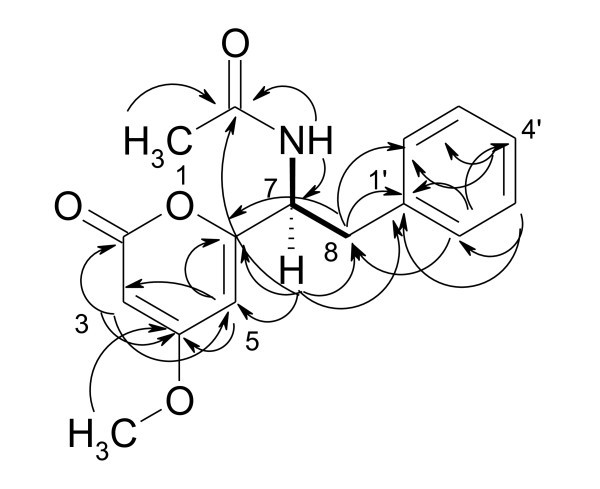
**Selected HMBC (→) and H, H-COSY (bold lines) correlations of pyrophen (1)**.

The molecular formula of compound **1 **was determined by HRMS as C_16_H_17_NO_4_; the ^1^H NMR spectrum revealed signals for a phenyl residue, an amino NH doublet, and two *m*-coupled methines (*δ *5.90, 5.43). Further signals were a methine quartet, a methylene 2H multiplet and two methyl singlets. The ^13^C NMR/HMQC spectra indicated the existence of 16 carbons corresponding to a phenyl residue, 2 up-field *sp*^2 ^methines (*δ*100.6, 88.0), 4 quaternary *sp*^2 ^atoms (*δ*171.0-161.9), representing carbonyls or phenolic carbons, and 4 *sp*^3 ^carbon signals (*δ*55.7-22.3). According to these data, compound **1 **was identified as pyrophen (**1**) [5], which was isolated and reported previously from *Aspergillus niger *[18,19] and elucidated by crystal structure analysis. Here, we report the full NMR assignments data for **1 **using the 2D NMR experiments for the first time (Figure 1 and Table 1 [see Additional file 1]).

**Table 1:** ^13^C and ^1^H NMR data of pyrophen (1) in CDCl_3 _(*J *in [Hz])

Number	δ_c_	δ_h_	Number	δ_c_	δ_h_
2	164.7	-	7-NHCOCH_3_	170.3	-
3	88.0	5.43 (d, 2.2)	7-NHCOCH_3_	22.3	1.95 (s)
4	171.0	-	8	38.1	3.09 (m)
4-OCH_3_	55.7	3.73 (s)	1'	136.0	-
5	100.6	5.90 (d, 2.2)	2',6'	128.6	7.16 (m)
6	161.9	-	3',5'	128.2	7.25 (m)
7	52.3	4.98 (q, 7.8)	4'	126.5	7.21 (m)
7-NHCOCH_3_	-	7.79 (d, 8.4)			

**Figure 2:**
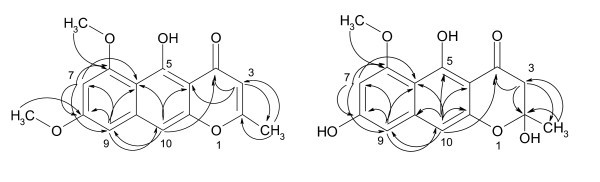
**HMBC couplings in Rubrofusarin B (2) and Fonsecin (3)**.

Compound **2 **showed a molecular weight of *m/z *287.09137 (HRESI MS), corresponding to the molecular formula C_16_H_15_O_5 _[M+H]^+^. The ^1^H NMR spectra (Table 2) displayed a chelated hydroxyl group (*δ *14.96), two *m-*coupled doublets (*δ *6.56, 6.38) and two singlets (*δ *6.94 and 5.98), along with two methoxy signals (*δ *3.99, 3.91) and an *sp*^2 ^linked methyl (*δ *2.35). The ^13^C/HMQC spectra (Table 2) indicated the presence of 16 carbon signals, including 4 *sp*^2 ^methines (*δ *107.3-97.2), 3 *sp*^2^-oxy carbons (*δ *162.6-160.6), 1 carbonyl of γ -lactone (*δ *184.2) [20], 5 non-oxygenated *sp*^2^, 2 aromatic-attached methyl ethers (*δ *56.0, 55.4) and 1 *sp*^2^-attached methyl (*δ *20.6). Full assignment of the 2D NMR experiments (Figure 2 and Table 2) established the structure of **2 **as rubrofusarin B, and excluded the structure of the isomeric asperxanthon (**11**) in the same way [21]. Structure of **2 **was not fully assigned using 2D NMR before, which we report her to first time (see Additional file 2).

**Table 2:** ^13^C and ^1^H NMR data of rubrofusarin B (2) and fonsecin (3) in CDCl_3 _(*J *in [Hz])

Number	2		3	
	
	*δ*_c_	*δ*_H_	*δ*_c_	*δ*_H_
2	167.4	-	100.0	-
2-CH_3_	20.6	2.35 (s)	27.6	1.60 (s)
2-OH	-	-	-	6.95 (brs)
3	107.3	5.98 (s)	47.6	3.14 (d, 16.8), 2.72 (d, 16.8)
4	184.2	-	197.5	-
4a	104.3	-	102.5	-
5	162.6	-	164.2	-
5-OH	-	14.96 (s)	-	14.19 (s)
5a	108.4	-	105.2	-
6	160.6	-	161.4	-
6-OCH3	56.0	3.99 (s)	55.6	3.84 (s)
7	97.2	6.38 (d, 2.2)	96.6	6.31 (brd, 1.1)
8	161.5	-	160.7	-
8-OH	-	-	-	10.18 (brs)
8-OCH3	55.4	3.91 (s)	-	-
9	97.8	6.56 (d, 2.2)	101.5	6.47 (s)
9a	141.0	-	142.9	-
10	101.0	6.94 (s)	101.0	6.41 (s)
10a	153.3	-	153.4	-

The closely related compound **3 **afforded a molecular weight of 290 Da (C_15_H_12_O_5 _by HRESI MS); EI MS gave easily an ion peak at *m/z *272 by expulsion of water molecule. The ^1^H NMR spectrum exhibited aromatic *m*-coupled doublets (*δ *6.47, 6.31, *J *~ 1.1 Hz) and a methine singlet (*δ *6.41), but in contrast to **2**, two phenolic hydroxy signals (*δ *14.19, 10.18), and only one methoxy signal (*δ *3.84). In addition, an AB signal of diastereotopic methylene protons (*δ *3.14, 2.72, *J *~ 16.8) and a methyl singlet (*δ *1.60) were visible. Based on ^13^C/HMQC spectra (Table 2) and HMBC experiment (as it was not fully assigned before using 2D NMR) (Figure 2), compound **3 **was finally established as fonsecin (**3**) (see Additional file 3). The facile loss of water by EI MS corresponded to the formation of TMC-256 A1 (**4**).

Compound **5 **displayed similar chromatographic properties and the same ^1^H NMR pattern as **3**. The molecular weight of **5 **was deduced as 304 Da, which is 14 *amu *higher than that of **3**, attributing to the methylation of the phenolic hydroxyl group at 8-position, hence compound **5 **was identified as fonsecin B [22] (see Additional file 4)

### 3.1. Aurasperones A-C and F

Compound **6 **was obtained from fraction II as middle polar yellow solid, displaying a molecular weight at *m/z *570. The expectation of a dimeric rubrofusarin B (**2**) was confirmed by ^1^H NMR spectra, where six *sp*^2 ^methine protons were visible, which were classified into two *m*-coupled protons, two α-methines of the consequent γ -pyrones (*δ *6.15, 6.08) and two singlet methines (*δ *7.35 and 7.24), together with six methyls, among them four methoxy signals. Based on these data and search in literature, compound **6 **was identified as aurasperone A [22] (see Additional file 5)

Compound **7 **exhibited a close structural similarity with fonsecin B (**5**); the molecular weight was determined as 606 Da, corresponding to the molecular formula C_32_H_30_O_12 _(HRESI MS). EI MS of **7 **displayed an ion signal at *m/z *570 as base peak, resulting from the expulsion of two water molecules, affording the molecular weight of aurasperone A (**6**). The ^1^H NMR spectrum established a dimeric pattern of fonsecin B (**5**), where four *sp*^2 ^methines protons being of two *m*-coupled protons and two singlet methines; two methylene signals (*δ *3.02 and 2.89) instead of the two α-methines of the consequent-γ-pyrones shown in **6**, along with six methyls, among them four methoxy signals and two *sp*^3^-bounded methyl signals (*δ *1.79, 1.46). In accordance, structure of **7 **was assigned as aurasperone B (**7**) [22]. (see Additional file 6)

A third dimer **8 **had a molecular weight of 592 Da and a corresponding molecular formula C_31_H_28_O_12_. Three consecutive fragment ions (*m/z *574, 556 and 525) on EI MS corresponded to the expulsion of one H_2_O molecule (to afford aurasperone F, **9**), two H_2_O (dianhydroaurasperone C, **10**) and 2H_2_O + OCH_3_, respectively. The ^1^H NMR spectrum displayed the same pattern as in aurasperone B (**7**), except that the methoxy signal (*δ *3.78) of 8-OCH_3 _in **7 **was replaced by a phenolic hydroxyl group, pointing to aurasperone C (**8**) [23]. (see Additional file 7)

Compound **9 **was a fourth dimer with a molecular formula C_31_H_26_O_11_; on EI MS, it displayed a fragment ion at *m/z *556 corresponding to an aromatized structural analogue (dianhydroaurasperone C, **10**), and a further fragment at *m/z *286 corresponded to rubrofusarin B (**2**). The ^1^H NMR spectra displayed five *sp*^2 ^methines (*δ *6.87-6.08), one less than in **6**, replaced by an *AB *signal of a methylene group (*δ *3.35-3.25). Accordingly, one of the β-bounded methyls of the lactones was up-field shifted (*δ *1.65), while the other one was retained at *δ *2.16 as in **6**. In contrast to **6**, only three methoxy signals (*δ *3.95-3.43) were visible, while the fourth one was replaced by a phenolic OH. Based on these spectroscopic features, structure **9 **was confirmed as aurasperone F [24] (see Additional file 8)

The four dimeric naphtho-*γ*-pyrones (**6-9**) were presently constructed from two naphtho-*γ*-pyrone units, which are not symmetrically linked; i.e. the first pyrone (above) unit is linked *via *a middle aromatic moiety (10'-position) to a terminal aromatic residue (7-position) of the second pyrone (down) unit.

The optical rotations of the dimers had the same negative sign and similar values indicating that the optical rotation value was dominated by the chiral axes between the two naphthopyranone moieties (atropisomerism). The absolute configurations of dimeric naphtho-*γ*-pyrones have been determined by circular-dichroism (CD). According to the literature [25], (*S*)-configured dimeric naphtho-*γ*-pyrones exhibit a first positive Cotton Effect in the long-wavelength region, a negative Cotton Effect at middle wavelength and then a positive Cotton Effect at shorter one. In our experimental data, the CD spectra for three representative dimeric naphtho-*γ*-pyrones (**6**-**8**) showed closely related values with pronounced Cotton Effects, recognizing them to have the same patterns. In accordance, the ellipticity of aurasperones A-C (**6-8**) showed three Cotton Effects, one peak was shown firstly in the region of [*θ*]_284-285 _+359274-22843.4, then one trough between [*θ*]_270-267 _-151670-339938 and the last elliptical peak was shown at [*θ*]_227-219 _+107899-5629. As the dimer **6 **has no further chiral elements, the chiral axis is dominating the absolute configuration. Based on the revealed features from the CD spectroscopic data, the four dimeric compounds (**6-9**) have identical (*S*)-configurations around their corresponding axis between C-10' and C-7 (see Additional file 9)

### 3.2. Biological activities

The antibiotic activity of compounds **1**-**8 **was examined against 11 microbial test organisms using the agar diffusion method (40 μg/disc) (Table 3). According to the antimicrobial assay, the crude extract of the fungal strain exhibited high activity against bacteria and yeasts (Table 4). Nevertheless, only three of the isolated metabolites were found to exhibited activity: pyrophen (**1**) and rubrofusarin B (**2**) displayed high (28 mm) and moderate (12 mm) activity against *C. albicans*, respectively, while aurosperone A (**6**) was active (13 mm) against the plant pathogenic fungi, *Rhizoctonia solani*. In the brine shrimp assay (10 μg/mL), all studied compounds here showed weak cytotoxicity (approx. 4-11%).

**Table 3:** Antimicrobial (40 μg/disc (∅ 9 mm; [mm]) and cytotoxic (10 μg/mL) activities of compounds 1-8

Compound number	BS^a^	SA^b^	SV^c^	EC^d^	CA^e^	MM^f^	CV^g^	CS^h^	SS^i^	PS^j^	PU^k^	Brine shrimp
**1**	ND	ND	ND	ND	28	ND	ND	ND	ND	ND	ND	4.2%
**2**	ND	ND	ND	ND	12	ND	ND	ND	ND	ND	ND	11%
**3**	ND	ND	ND	ND	ND	ND	ND	ND	ND	13	ND	Nt
**4**	ND	ND	ND	ND	ND	ND	ND	ND	ND	ND	ND	8.8%
**5**	ND	ND	ND	ND	ND	ND	ND	ND	ND	ND	ND	5.0%
**6**	ND	ND	ND	ND	ND	ND	ND	ND	ND	ND	ND	9.7%
**7**	ND	ND	ND	ND	ND	ND	ND	ND	ND	ND	ND	6.4%
**8**	ND	ND	ND	ND	ND	ND	ND	ND	ND	ND	ND	9.7%

^a^*Bacillus subtilis*, ^b^*S. aureus*, ^c^*Streptomyces viridochromogenes *(Tü 57), ^d^*Escherichia coli*, ^e^*C. albicans*, ^f^*Mucor miehi*, ^g^*Chlorella vulgaris*, ^h^*Chlorella sorokiniana*, ^i^*Scenedesmus subspicatus*, ^j^*R. solani; *^k^*Pythium ultimum*

ND, not detected.

**Table 4:** Antimicrobial activities of the fugal extract (60 μg/disc (5-mm diameter)

Test organism	Extract activity (mm)
*P. aeruginosa*	17

*S. aureus*	23

*C. albicans*	20

*A. niger*	ND

ND, not detected.

## 4. Experimental

The NMR spectra were measured on a Bruker AMX 300 (300.135 MHz), a Varian Unity 300 (300.145 MHz) and Varian Inova 500 (499.876 MHz) spectrometers. EI mass spectra were recorded on a Finnigan MAT 95 spectrometer (70 eV). ESI MS was recorded on a Finnigan LCQ with quaternary pump Rheos 4000 (Flux Instrument). HRMS were recorded by ESI MS on an Apex IV 7 Tesla Fourier-Transform Ion Cyclotron Resonance Mass Spectrometer (Bruker Daltonics, Billerica, MA, USA). Optical rotation was measured on a Perkin-Elmer Polarimeter, model 343. Flash chromatography was carried out on silica gel (230-400 mesh). *R*_f _values were measured on Polygram SIL G/UV_254 _(Macherey-Nagel & Co., Düren, Germany). Size exclusion chromatography was done on Sephadex LH-20 (Lipophilic Sephadex, Amersham Biosciences Ltd.; purchased from Sigma-Aldrich Chemie, Steinheim, Germany).

### 4.1. Sampling and isolation of the fungal strain

The reddish soft coral *D. hemprichi *was collected from the Red Sea; approx. 30 km offshore from Safaga (east Egypt) at a depth of approx. 30 m. Pieces of the coral were rinsed three times with sterile seawater and then aseptically cut into smaller pieces and shaken for 2 h. The aqueous supernatant was serially diluted, and each 200 μL were inoculated onto 15-cm Petri dishes, each containing 50 mL of yeast extract/starch agar (yeast extract 0.2 g/L, soluble starch 1.0 g/L, agar 20 g/L, chloramphenicol 50 mg/L natural seawater at pH 6.0) [7]. The black single colonies were picked from the plates after inoculation for 25 days at 30°C and sub-cultured on the same medium without chloramphenicol. The strain is deposited in the culture collection of the Department of Microbial Chemistry, NRC, Cairo, Egypt.

### 4.2. Fermentation and working up

The well-grown single colonies of *A. alternata *were inoculated in subculture agar slants containing malt extract medium: malt extract (30 g/L), peptone 5 g/L), agar (20 g/L), natural sea water (1000 mL); at pH approx. 5.5 for 7 days at 30°C). The obtained grown agar slants were served to inoculate 500-mL Erlenmeyer flasks, each containing 100 mL of GYMP medium (g/L): malt extract (3), yeast extract (3), peptone (5), glucose (10) and 1000 mL natural seawater at pH approx. 6.5 at 30°C. The culture media was in turn applied to cultivation on a rotary shaker (10 days). After harvesting, the afforded black broth was centrifuged (7,000 rpm for 15 min), and the obtained two phases, mycelial cake and supernatant, were individually extracted with ethyl acetate. The obtained unique black organic extracts were applied to biological and chemical screenings.

The well-grown agar slants of the fungal strain D2006 were served to inoculate 60 of 1-L Erlenmeyer flasks, each containing 300-mL of GYMP medium (g/L): malt extract (3), yeast extract (3), peptone (5), glucose (10), agar (20) and 1000 mL of 100% seawater at pH approx. 6.5. The inoculated media was applied to additional cultivation using a rotary shaker (150 rpm) for 10 days. After harvesting, the obtained black culture broth was mixed with celite (approx. 1.5 kg) and then filtered *in vacuo*. The afforded two phases, filtrate and mycelium, were applied to exhaustive extraction by ethyl acetate. TLC of both organic extracts recognized their unique, and they were combined therefore, and concentrated *in vacuo*, affording 5.5 g as black crude extract.

### 4.3. Isolation of the active constituents

The obtained extract was applied to column chromatography on silica gel eluted by CH_2_Cl_2_-MeOH gradient and monitored by TLC to afford five fractions: I (0.62 g), II (1.21 g), III (0.71 g), IV (1.52 g) and V (0.22 g). Fraction I was re-purified on silica gel column (DCM) followed by Sephadex LH-20 (DCM/40% MeOH) to afford a colourless semisolid of phyrophen (**1**) (468.0 mg). Application of Fraction II to PTLC (DCM/3% MeOH) followed by purification on Sephadex LH-20 (DCM/40% MeOH) lead to isolation of two yellow solids of rubrofusarin B (**2**, 11.0 mg) and aurosperone A (**6**, 13.0 mg), respectively. Fraction III was purified using a silica gel column (DCM-MeOH) followed by Sephadex LH-20 (DCM/40% MeOH) to give a yellow solid of aurasperone F (**9**, 15.0 mg). Purification of the middle polar fraction IV *via *PTLC (DCM/5% MeOH) followed by Sephadex LH-20 (MeOH) yielded three yellow solids of aurasperone B (**7**, 8.0 mg), aurasperone C (**8**, 14.0 mg) and fonsecin (**3**, 11.5 mg). As the same for IV, the polar fraction V afforded three yellow solids of fonsecin B (**5**, 12.0 mg), aurasperone B (**7**, 3.4 mg) and aurasperone C (**8**, 4.1 mg).

#### Pyrophen (1)

Colourless semisolid, UV-absorbing, no colour reaction on spraying with anisaldehyde/sulphuric acid; *R*_f _= 0.86 (CH_2_Cl_2_/5% MeOH); **^1^H NMR **(300 MHz, CDCl_3_) and **^13^C NMR **(125 MHz, CDCl_3_) see Table 1; **EI MS ***m/z *(%) = 287.2 ([M]^+^, 28), 228.1 (8), 196.1 (40), 154.2 (100), 125.1 (16), 111.1 (6), 91.1 (12), 43.1 (11); **(+)-ESI MS ***m/z *(%) = 596.9 ([2M+Na]^+^, 85), 310 [M+Na]^+^, 36), 288 ([M+H]^+^, 100); **(-)-ESI MS ***m/z *286 [M+H]^-^; **(+)-HRESI MS ***m/z *288.12301 ([M+H]^+^, calcd: 288.12303 for C_16_H_18_NO_4_); 310.10490 ([M+Na]^+^, calcd: 310.10497 for C_16_H_17_NO_4_Na).

#### Rubrofusarin B (2)

Yellow solid, UV-green fluorescence (365 nm), orange with anisaldehyde/sulphuric acid; *R*_f _= 0.78 (CH_2_Cl_2_/5% MeOH); **^1^H NMR **(300 MHz, CDCl_3_) and **^13^C NMR **(125 MHz, CDCl_3_) see Table 2; **EI MS ***m/z *(%) = 286.2 ([M]^+.^, 100), 268.1 ([M-H_2_O]^+^, 12), 257.2 ([M-CHO]^+^, 44), 240.2 (8) 213.2 (5), 43.1 (7); **(+)-ESI MS ***m/z *(%) = 594.9 ([2M+Na]^+^, 14), 287 ([M+H]^+^, 100); **(+)-HRESI MS ***m/z *287.09137 ([M+H]^+^, calcd: 287.09139 for C_16_H_15_O_5_).

#### Fonsecin (3)

Yellow solid, UV-green fluorescence (365 nm), turned dark red with anisaldehyde/sulphuric acid; *R*_f _= 0.38 (CH_2_Cl_2_/5% MeOH); **^1^H NMR **(300 MHz, CDCl_3_) and **^13^C NMR **(125 MHz, CDCl_3_) see Table 2; **EI MS ***m/z *(%) = 290.2 ([M]^+^, 24), 272.2 ([M-H_2_O]^+^, 16), 243.2 (8), 232.1 (21), 189.1 (7), 175.1 (16), 101.1 (15), 85.1 (22), 59.1 (36), 43.1 (100); **(+)-ESI MS ***m/z *(%) = 291 ([M+H]^+^); **(-)-ESI MS ***m/z *(%) = 289 ([M-H]^-^); **(+)-HRESI MS ***m/z *291.08631 ([M+H]^+^, calcd: 291.08631 for C_16_H_15_O_6_).

#### Fonsecin B (5)

Yellow solid, UV-green fluorescence (365 nm), turned dark red with anisaldehyde/sulphuric acid; *R*_f _= 0.44 (CH_2_Cl_2_/5% MeOH); **^1^H NMR **(300 MHz, DMSO-*d*_6_) *δ *= 14.09 (brs, 1H, 5-OH), 7.00 (brs, 1H, 2-OH), 6.68 (brd, 1H, *J *~ 1.1 Hz, H-9), 6.55 (s, 1H, H-10), 6.38 (brd, 1H, *J *~ 1.1 Hz, H-9), 3.84 (s, 6H, 6,8-OCH_3_), 3.14 (d, 1H, *J *~ 16.8 Hz, H-3a), 2.72 (d, 1H, *J *~ 16.8 Hz, H-3b), 1.61 (s, 3H, 2-CH_3_); **EI MS ***m/z *(%) = 304.3 ([M]^+^, 56), 286.3 ([M-H_2_O]^+^, 8), 262.3 (8), 247.2 (28), 246.2 (60), 220.2 (20), 218.2 (10), 149.2 (20), 145.2 (34), 127.2 (12), 116.2 (64), 101.2 (48), 84.1 (36), 66.1 (24), 59.1 (63), 43.1 (100).

#### Aurasperone A (6)

Yellow solid, UV-green fluorescence (365 nm), turned orange with anisaldehyde/sulphuric acid; *R*_f _= 0.82 (CH_2_Cl_2_/5% MeOH); [α]_D_^20 ^= -18.9 (*c *= 0.19, MeOH); CD (c 1.1929 × 10^-5 ^mol/L [c 6.8 μg/mL], MeOH) [*θ*]_400 _0 [*θ*]_284 _+22843, [*θ*]_270 _-36396, [*θ*]_219 _+5629; **^1^H NMR **(300 MHz, CD_3_OD) *δ *= 7.35 (s, 1H, H-10), 7.25 (s, 1H, H-9), 6.51 (brd, 1H, *J *~ 1.1 Hz, H-7'), 6.23 (brd, 1H, *J *= 1.1 Hz, H-7), 6.15 (s, 1H, H-3), 6.08 (s, 1H, H-3'), 3.95 (s, 3H, 6'-OCH_3_), 3.79 (s, 3H, 8-OCH_3_), 3.59 (s, 3H, 8'-OCH_3_), 3.46 (s, 3H, 6-OCH_3_), 2.42 (s, 3H, 2-CH_3_), 2.13 (s, 3H, 2'-CH_3_); **EI MS ***m/z *(%) = 570.5 ([M]^+^, 44), 539.5 ([M-OCH_3_]^+^, 10), 513.4 (5), 286 (7), 167.2 (7), 145.2 (44), 116.2 (100), 85.1 (39), 55.1 (22), 43.1 (24).

#### Aurasperone B (7)

Yellow solid, UV-green fluorescence (365 nm), turned orange with anisaldehyde/sulphuric acid; *R*_f _= 0.48 (CH_2_Cl_2_/5% MeOH); [α]_D_^20 ^= -18.3 (*c *= 0.12, MeOH); CD (c 2.83 × 10^-5 ^mol/L [c 17.2 μg/mL], MeOH) [*θ*]_400 _0, [*θ*]_284 _+143232, [*θ*]_267 _-151670, [*θ*]_227 _+46610; **^1^H NMR **(300 MHz, CDCl_3_) *δ *= 14.51 (brs, 1H, 5'-OH), 14.08 (brs, 1H, 5-OH), 6.84 (s, 1H, H-9), 6.72 (s, 1H, H-9), 6.37 (d, 1H, *J *~ 1.1 Hz, H-7'), 6.14 (d, 1H, *J *~ 1.1 Hz, H-9'), 3.99 (s, 3H, 6'-OCH_3_), 3.78 (s, 3H, 8-OCH_3_), 3.63 (s, 3H, 8'-OCH_3_), 3.39 (s, 3H, 6-OCH_3_), 3.02, (d, 2H, *J *~ 16.3 Hz, 3-H_2_), 2.89 (m, 2H, 3'-H_2_), 1.79 (s, 3H, 2-CH_3_), 1.46 (s, 3H, 2'-CH_3_); **EI MS ***m/z *(%) = 570.3 ([M-2H_2_O]^+^, 100), 539.4 ([M-(2H_2_O+OCH_3_)]**^+^**, 74), 524.3 (5), 299.2 (12), 272.2 (13), 269.7 (24), 230.2 (18), 193.1 (12), 154.2 (14), 149.1 (19), 130.1 (48), 91.1 (54), 57.1 (30), 43.1 (57); **(+)-HRESI MS ***m/z *607.18100 ([M+H]^+^, calcd: 607.18100 for C_32_H_31_O_12_), *m/z *629.16294 ([M+Na]^+^, calcd: 629.16295 for C_32_H_30_O_12_Na).

#### Aurasperone C (8)

Yellow solid, UV-green fluorescence (365 nm), turned orange with anisaldehyde/sulphuric acid; *R*_f _= 0.26 (CH_2_Cl_2_/5% MeOH); [α]_D_^20 ^= -33.5 (*c *= 0.17, MeOH); CD (c 4.29 × 10^-5 ^mol/L [c 24 μg/mL], MeOH) [*θ*]_400 _0, [*θ*]_285 _+359273, [*θ*]_268 _-339938, [*θ*]_226 _+107899.87; **^1^H NMR **(300 MHz, CD_3_OD): *δ *= 6.84 (s, 1H, H-10), 6.57 (s, 1H, H-9), 6.38 (d, 1H, *J *~ 1.2 Hz, H-9'), 6.20 (d, 1H, *J *~ 1.2 Hz, H-7'), 3.93 (s, 3H, 6'-OCH_3_), 3.60 (s, 3H, 8'-OCH_3_), 3.50 (s, 3H, 6-OCH_3_), 3.30-3.29 (m, 4H, 3,3'-H_2_), 1.69 (s, 3H, 2-CH_3_), 1.49 (s, 3H, 2'-CH_3_); **EI MS ***m/z *(%) = 574.3 ([M-H_2_O]^+^, 6), 556.3 ([M-2H_2_O]^+^, 42), 525.3 ([M-(2H_2_O+OCH_3_)]^+^, 32), 264.2 (7) 58.2 (28), 43.1 (100); **(+)-HRESI MS ***m/z *615.14779 ([M+Na]^+^, calcd: 615.14729 for C_31_H_28_O_12_Na), *m/z *593.16570 ([M+H]^+^, calcd: 593.16534 for C_31_H_29_O_12_).

#### Aurasperone F (9)

Yellow solid, UV-green fluorescence (365 nm), turned orange with anisaldehyde/sulphuric acid; *R*_f _= 0.55 (CH_2_Cl_2_/5% MeOH); **^1^H NMR **(300 MHz, CD_3_OD) *δ *= 6.87 (s, 1H, H-10), 6.55 (s, 1H, H-9), 6.51 (d, 1H, *J *~ 1.1 Hz, H-9'), 6.36 (brd, 1H, *J *~ 1.1 Hz, H-7'), 6.08 (s, 1H, H-3'), 3.95 (s, 3H, 6'-OCH_3_), 3.63 (s, 3H, 8'-OCH_3_), 3.43 (s, 3H, 6-OCH_3_), 3.35-3.25, (m, 2H, 3-H_2_), 2.16 (s, 3H, 2'-CH_3_), 1.65 (s, 3H, 2-CH_3_); **EI MS ***m/z *(%) = 556.5 ([M-H_2_O]^+^, 5), 286.3 ([rubrofusarin B (**2**)]**^+^**, 8), 84.1 (12), 57.2 (10), 44.1 (100); **(+)-ESI MS ***m/z *(%) = 1172 ([2M+Na+H]^+^, 19), 575 ([M+H]^+^, 100); **(-)-ESI MS ***m/z *(%) = 1721 ([3M-H]^-^, 31), 1147 ([2M-H]^-^, 22), 573 ([M-H]^-^, 100).

### 4.4. Biological activities

#### Antimicrobial activity

Compounds **1**-**8 **were dissolved in CH_2_Cl_2_/10% MeOH at a concentration of 1 mg/mL. Aliquots of 40 μL were soaked on filter paper discs (9 mm ∅, no. 2668, Schleicher & Schüll GmbH, Germany) and dried for 1 h at room temperature under sterilized conditions. The paper discs were placed on inoculated agar plats and incubated for 24 h at 38°C for bacterial and 48 h (30°C) for the fungal isolates, while the algal test strains were incubated at room temperature in day light.

For the fungal extract examination, representative test microbes; *P.aeruginosa, S. aureus, C. albicans *and *A. niger *were used. Both bacterial and yeast strains were grown on nutrient agar medium (g/L): Beef extract 3; peptone, 10; and agar, 20. The pH was adjusted to 7.2. The fungal strain was grown on Czapek-Dox medium (g/l): Sucrose, 30; NaNO_3_, 3; MgSO_4_.7H_2_O, 0.5l; KCl, 0.5; FeSO_4_, 0.01; K_2_HPO_4_, 1; and agar, 20. The pH was maintained at 6.0. The disc diffusion test has been done according to Collins and Lyne [26]. Filter paper discs (5 mm diameter) were saturated with 200 μ*g *from the culture extract, and located on the surface of the agar plates (150 mm diameter containing 50 mL of solidified media). The paper discs were placed on inoculated agar plats and incubated for 24 h at 38°C (bacteria and yeast) and 48 h at 30°C (fungi).

#### Brine shrimp microwell cytotoxicity assay

The cytotoxic assay was performed according to Takahashi et al. [27] and Sajid et al. [28].

## 5. Conclusions

In this research article, eight bioactive pyrone derivatives were identified from the culture of *A. alternata *strain D2006, isolated from the marine soft coral *D. hemprichi*. Selection of the strain was based on its profound antibiotic and antimicrobial activities. Structures of the isolated compounds were identified on the basis of 1D and 2D NMR spectroscopy and mass (EI, ESI, HRESI) data, and by comparison with the literature. Configuration of the four dimeric naphtha-γ-pyrones **6-9 **was analyzed by CD spectra, exhibiting an identical stereochemistry. The biological activity (antimicrobial and cytotoxicity) of the fungal extract and its corresponding isolated compounds were comparatively studied. This is as a trial to find out new leading drugs to overcome some of the recently discovered diseases.

## Competing interests

The authors declare that they have no competing interests.

## Supplementary Material

Additional file 1 **Spectral data of Pyrophen (1)**. Ten charts (chart 1-10) containing the mass (ESI, HRESI, EI MS) and NMR (^1^HNMR, ^13^CNMR, H, H COSY, HMQC, HSQC, HMBC) spectral data of Pyrophen (**1**). Click here for file

Additional file 2 **Spectral data of Rubrofusarin B (2)**. Thirteen charts (chart 11-23) containing the mass (ESI, EI, HRESI MS) and NMR (^1^HNMR, ^13^CNMR, H, H COSY, HMQC, HSQC, HMBC) spectral data of Rubrofusarin B (**2**). Click here for file

Additional file 3 **Spectral data of Fonsecin (3)**. Nine charts (chart 24-32) containing the mass (ESI, EI MS) and NMR (^1^HNMR, ^13^CNMR, H, H COSY, HMQC, HSQC, HMBC) spectral data of Fonsecin (**3**). Click here for file

Additional file 4 **Spectral data of Fonsecin B (5)**. Two charts (chart 33-34) containing the mass (EI MS) and NMR (^1^HNMR) spectral data of Fonsecin B (**5**). Click here for file

Additional file 5 **Spectral data of Aurasperone A (6)**. Two charts (chart 35-36) containing the mass (EI MS) and NMR (^1^HNMR) spectral data of Aurasperone A (**6**) Click here for file

Additional file 6 **Spectral data of Aurasperone B (7)**. Three charts (chart 37-39) containing the mass (HRESI, EI MS) and NMR (^1^HNMR) spectral data of Aurasperone B (**7**) Click here for file

Additional file 7 **Spectral data of Aurasperone C (8)**. Four charts (chart 40-43) containing the mass (ESI, HRESI MS) and NMR (^1^HNMR) spectral data of Aurasperone C (**8**) Click here for file

Additional file 8 **Spectral data of Aurasperone F (9)**. Three charts (chart 44-46) containing the mass (ESI, EI MS) and NMR (^1^HNMR) spectral data of Aurasperone F (**9**) Click here for file

Additional file 9 **CD Spectra of Aurasperones A-C (6-8)**. Three charts (chart 47-49) containing the CD spectral data of Aurasperones A-C (**6-8**). Click here for file
