# Supplementary material for: Seven naphtho-γ-pyrones from the marine-derived fungus Alternaria alternata: structure elucidation and biological properties
Source: Org Med Chem Lett. 2012 Feb 29;2:6. doi: 10.1186/2191-2858-2-6 (PMC3350997; doi:10.1186/2191-2858-2-6)
Supplement: Additional file 1 — Spectral data of Pyrophen (1). Ten charts (chart 1-10) containing the mass (ESI, HRESI, EI MS) and NMR (1HNMR, 13CNMR, H, H COSY, HMQC, HSQC, HMBC) spectral data of Pyrophen (1). [file 2191-2858-2-6-S1.DOC]

**Additional files**

**1. Additional file 1**

**Title:** Spectral data of Pyrophen (**1**).

**Description:** Ten charts (chart 1-10) containing the mass (ESI, HRESI, EI MS) and NMR (^1^HNMR, ^13^CNMR, H,H COSY, HMQC, HSQC, HMBC) spectral data of Pyrophen (**1**).

**
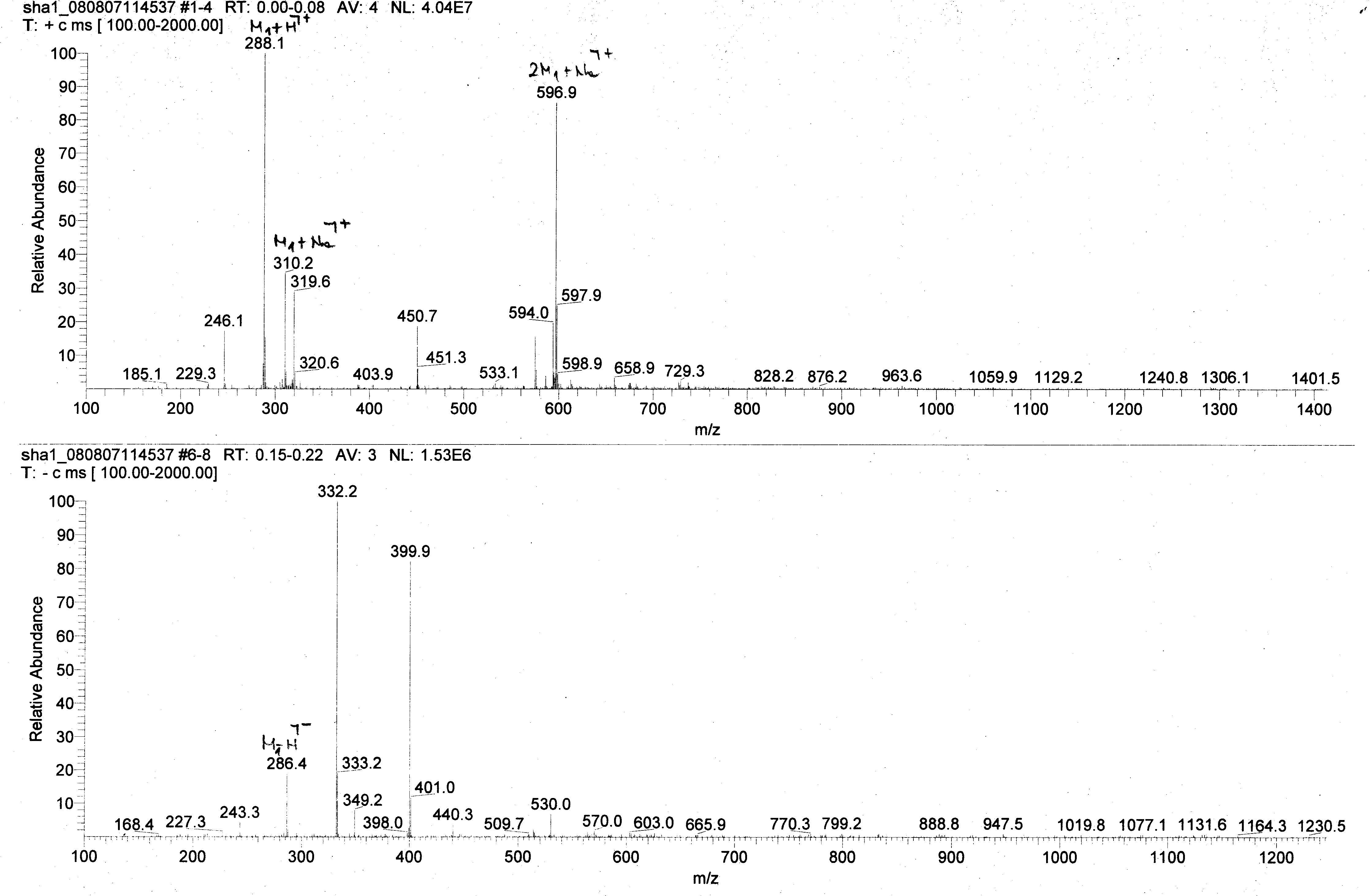
**

**Chart 1:** (+)-ESI MS and (-)-ESI MS spectra of Pyrophen (**1**)

**
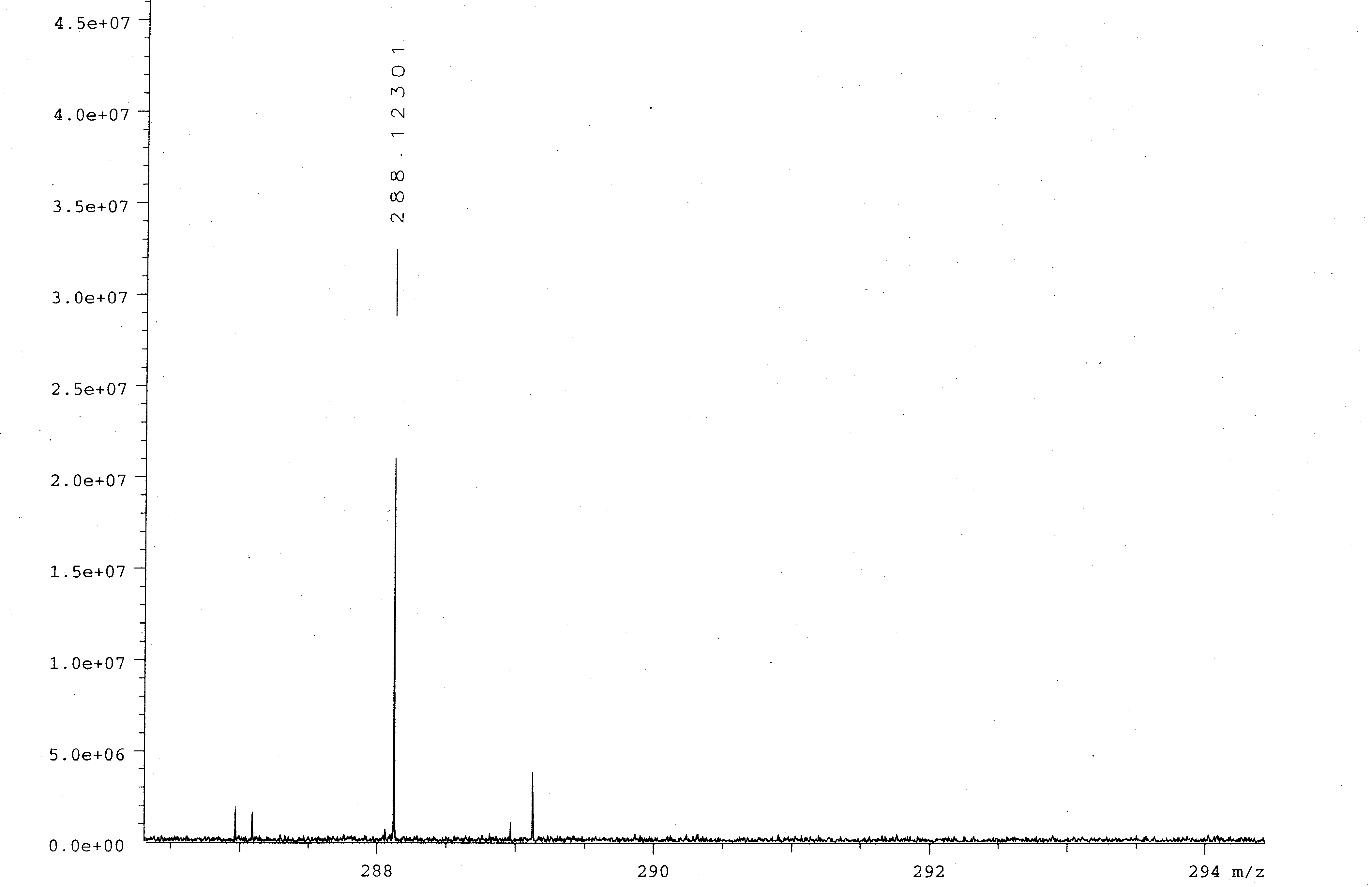
**

**Chart 2:** (+)-HRESI MS spectrum of Pyrophen (**1**)

**
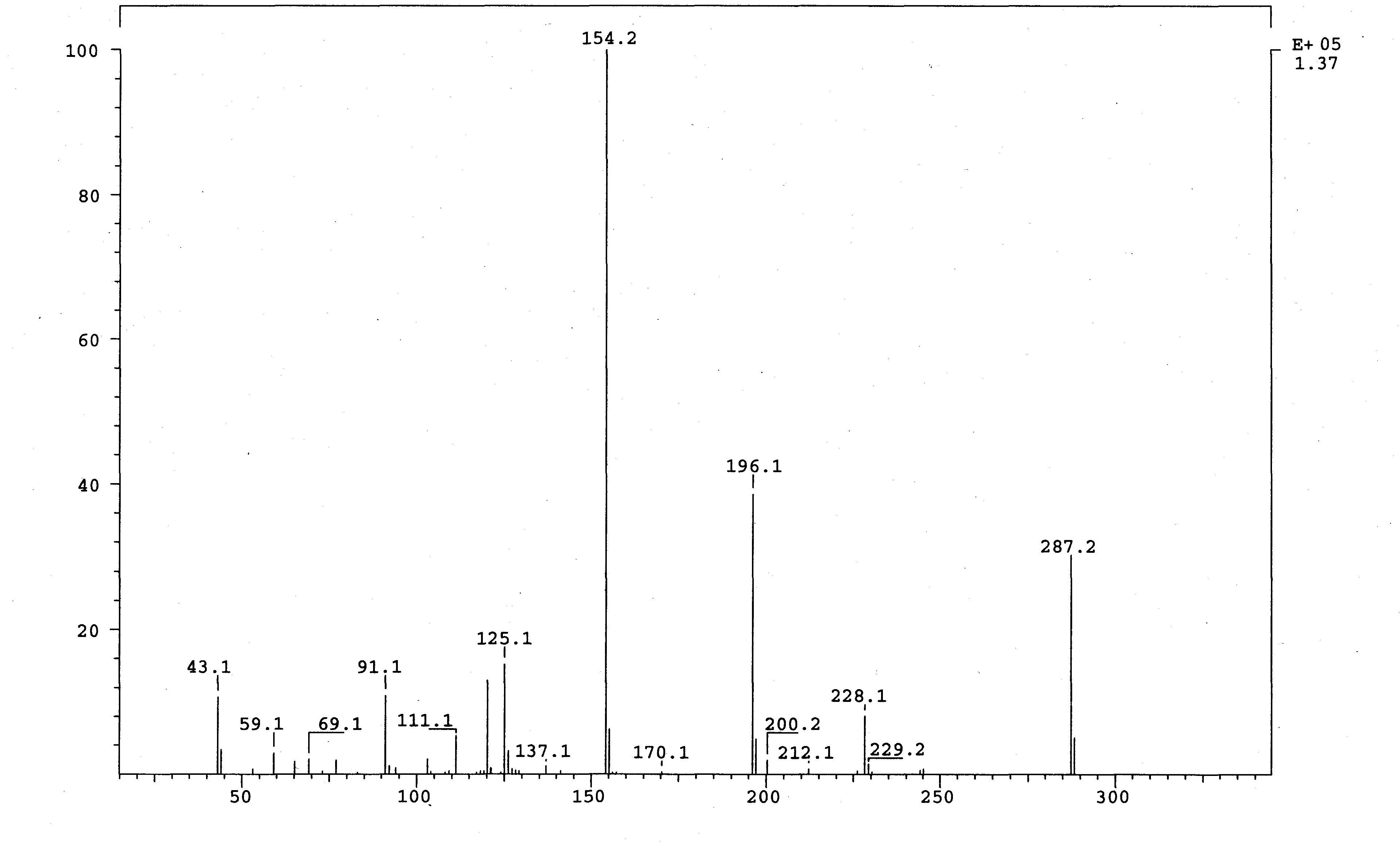
**

**Chart 3:** EI-MS spectrum of Pyrophen (**1**)

**
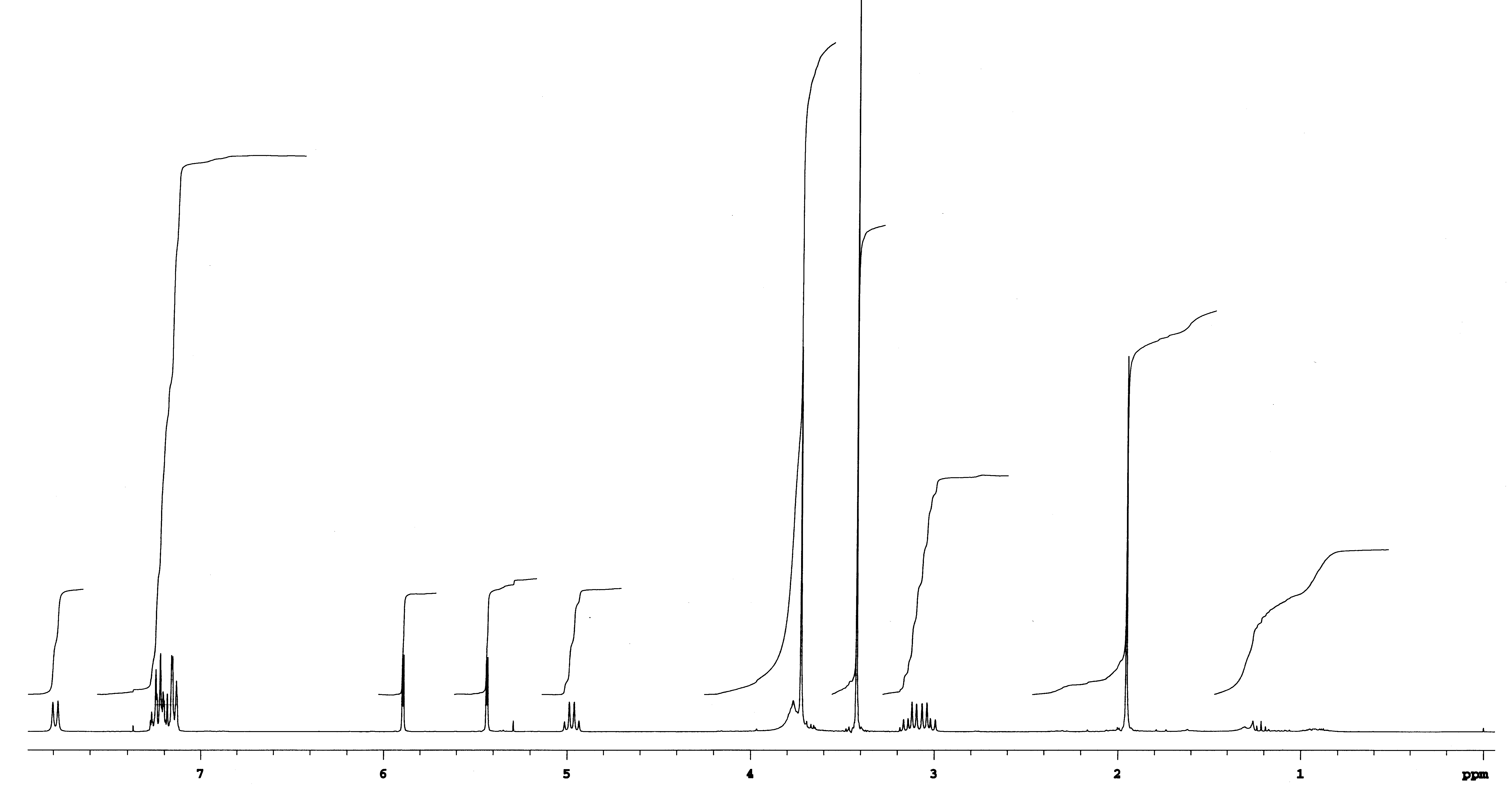
**

**Chart 4:** ^1^H NMR spectrum (CDCl_3_, 300 MHz) of Pyrophen (**1**)

**
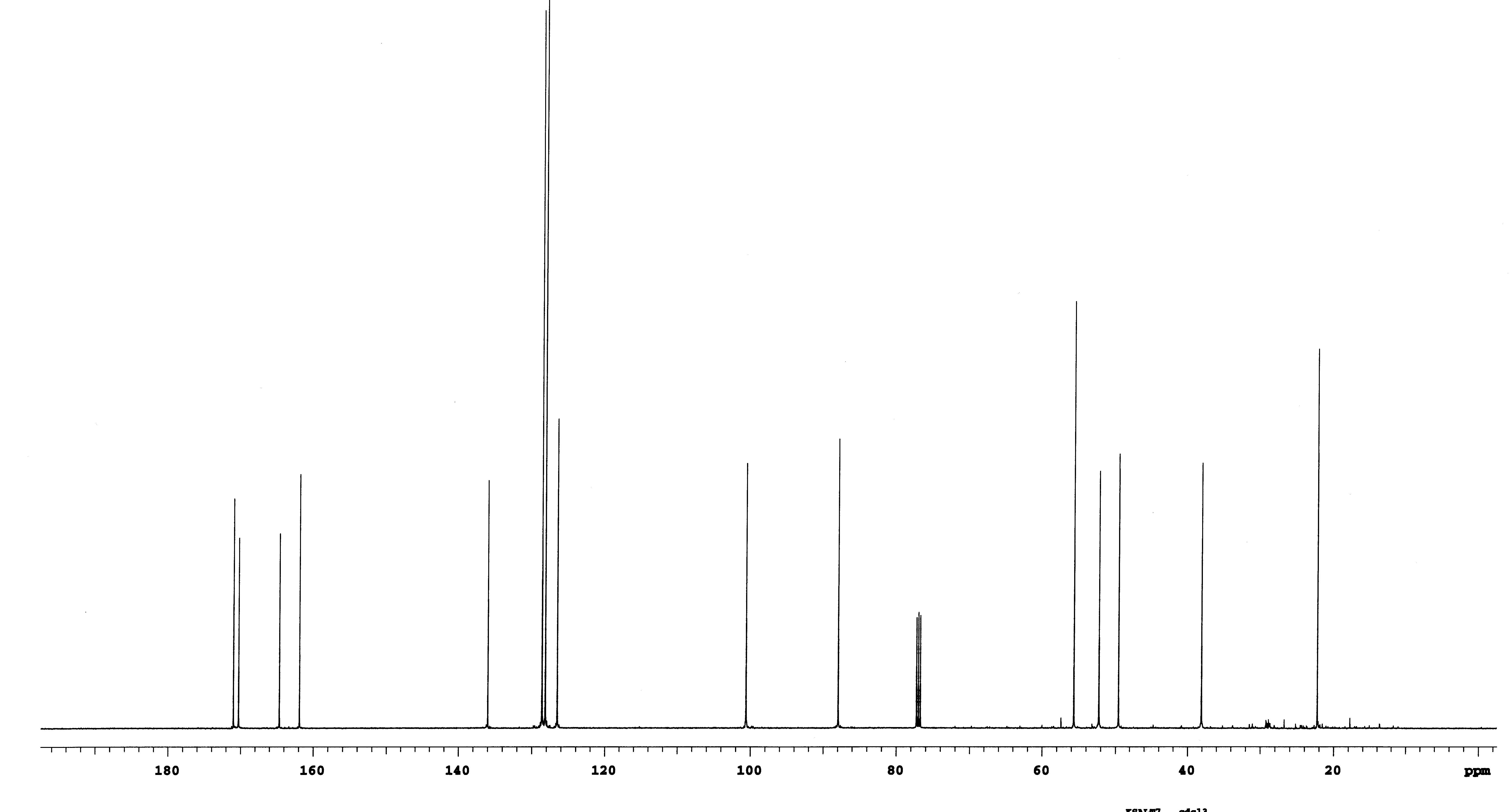
**

**Chart 5:** ^13^C NMR spectrum (CDCl_3_, 75 MHz) of Pyrophen (**1**)

**
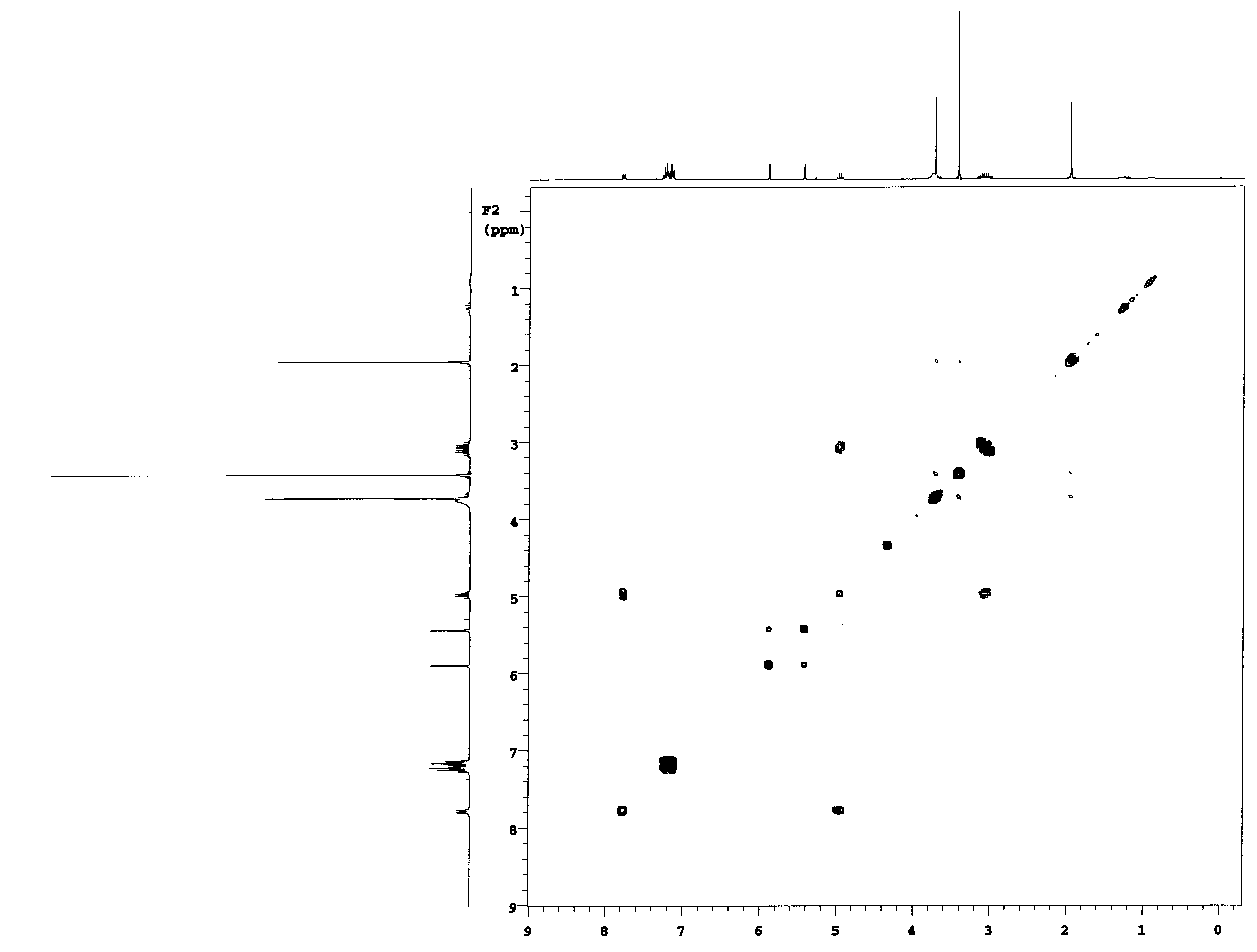
**

**Chart 6:** H,H COSY spectrum (CDCl_3_, 300 MHz) of Pyrophen (**1**)

**
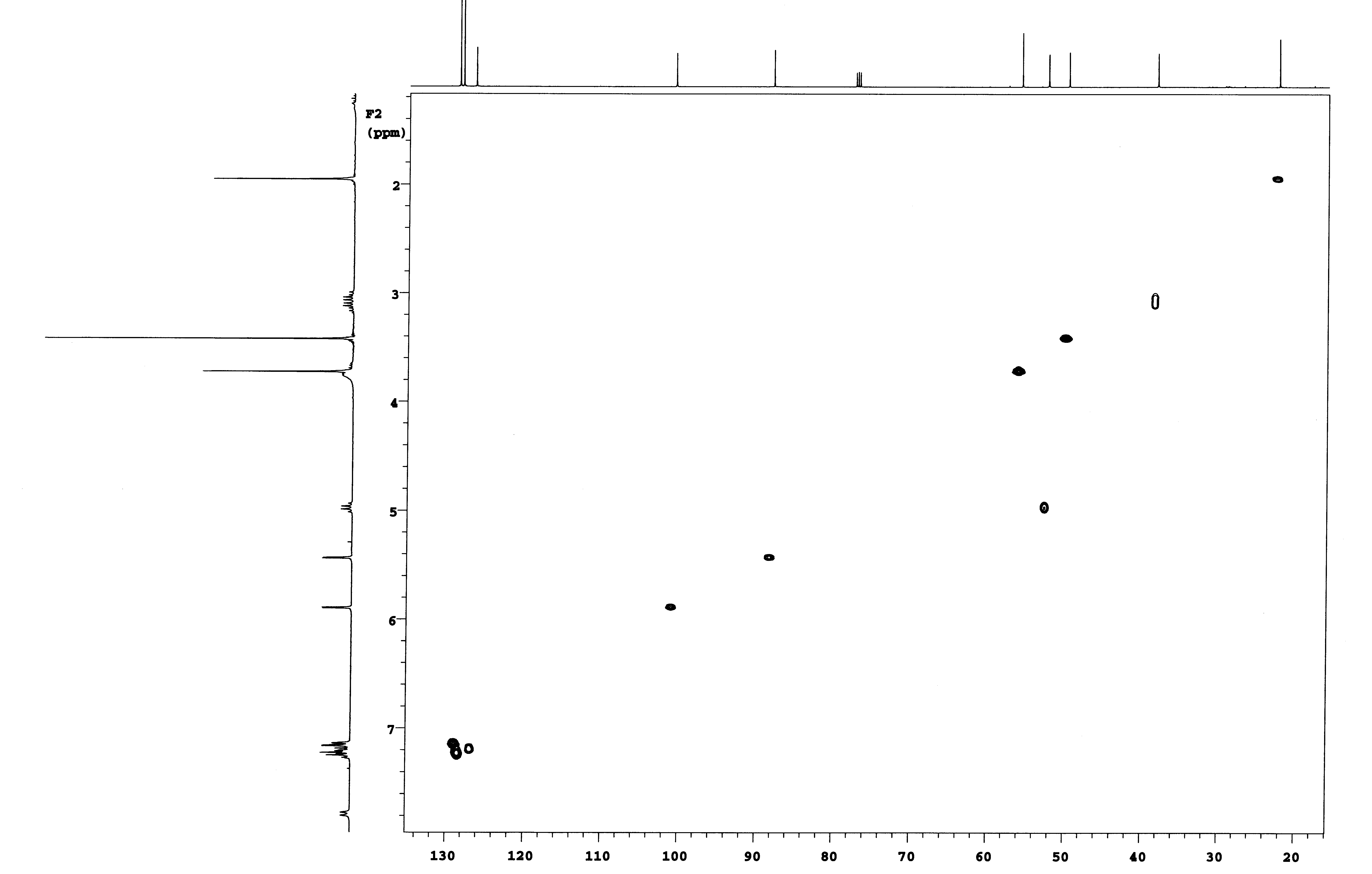
**

**Chart 7:** HMQC spectrum (CDCl_3_, 300 MHz) of Pyrophen (**1**)

**
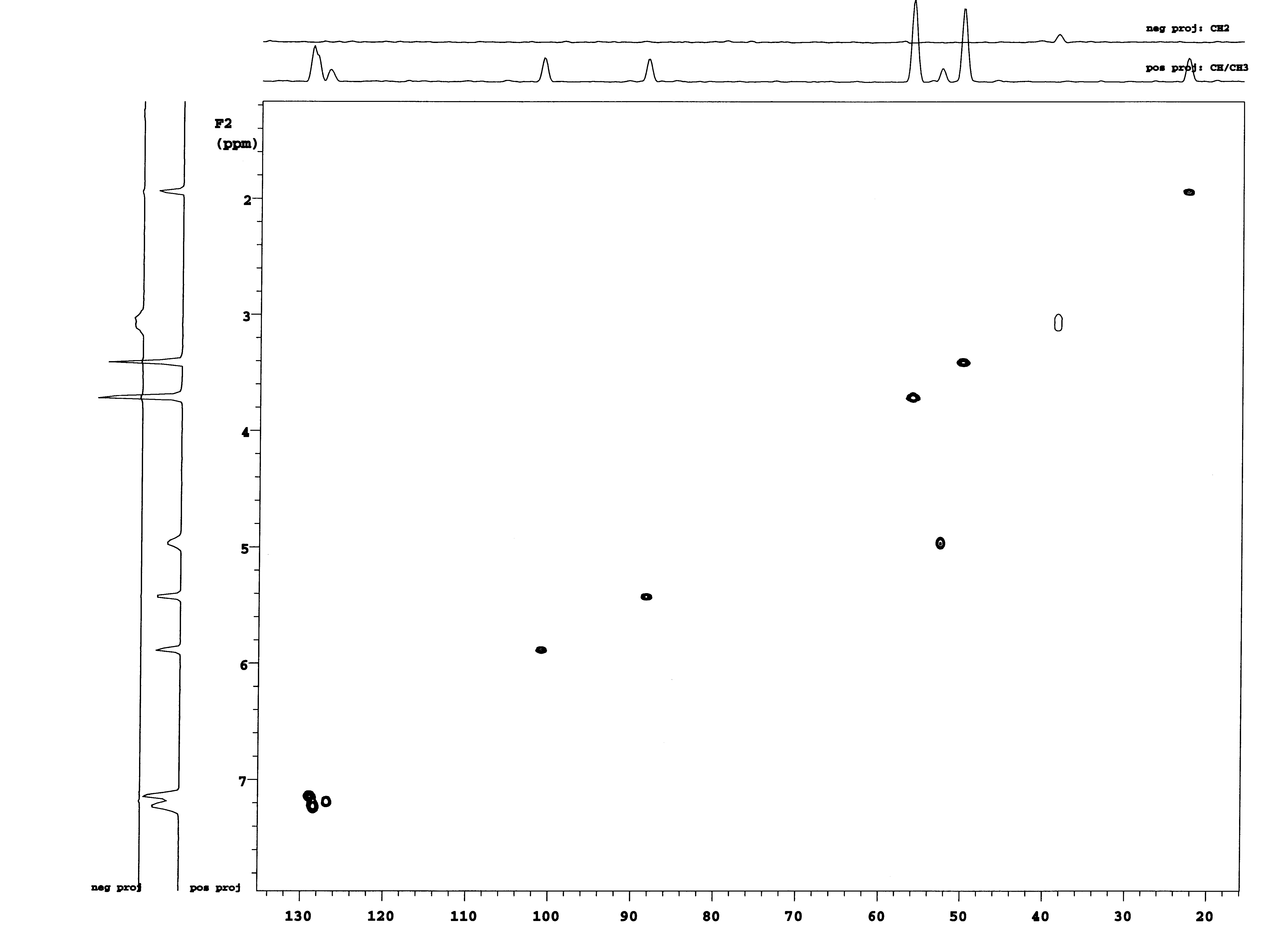
**

**Chart 8:** HSQC spectrum (CDCl_3_, 300 MHz) of Pyrophen (**1**)

**
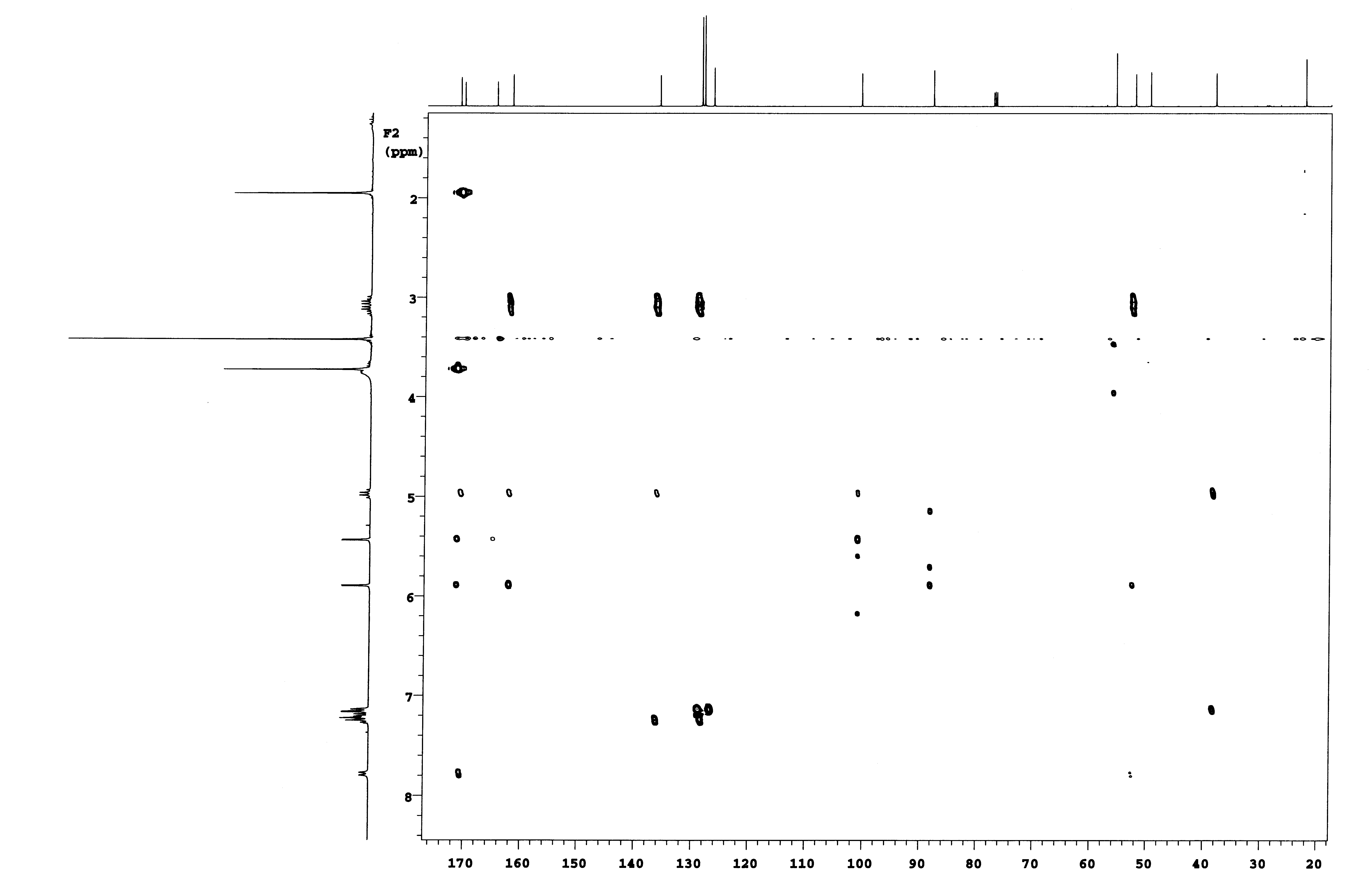
**

**Chart 9:** HMBC spectrum (CDCl_3_, 300 MHz) of Pyrophen (**1**)

**
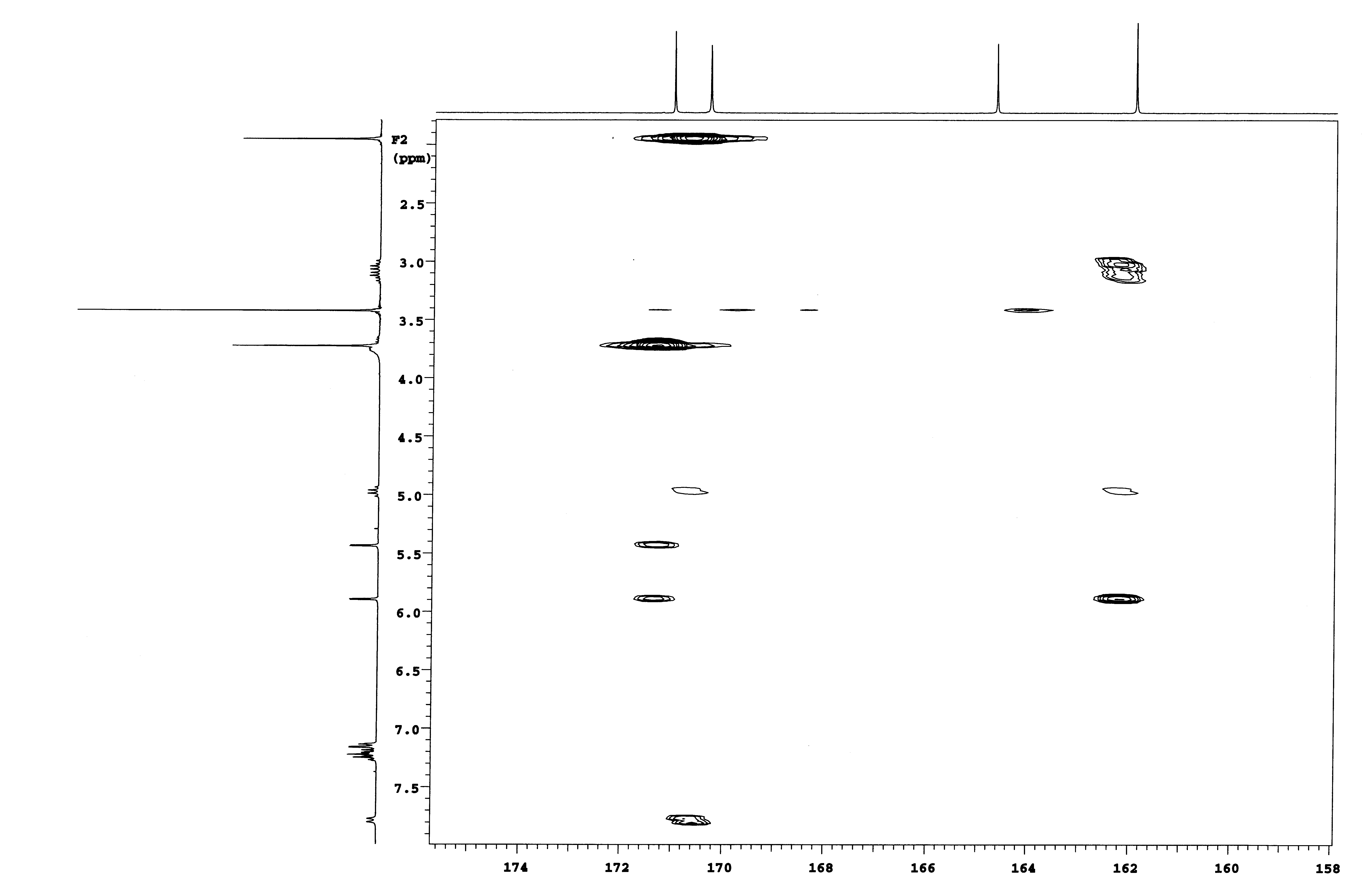
**

**Chart 10:** HMBC expansion spectrum (CDCl_3_, 300 MHz) of Pyrophen (**1**)
