# Supplementary material for: Seven naphtho-γ-pyrones from the marine-derived fungus Alternaria alternata: structure elucidation and biological properties
Source: Org Med Chem Lett. 2012 Feb 29;2:6. doi: 10.1186/2191-2858-2-6 (PMC3350997; doi:10.1186/2191-2858-2-6)
Supplement: Additional file 2 — Spectral data of Rubrofusarin B (2). Thirteen charts (chart 11-23) containing the mass (ESI, EI, HRESI MS) and NMR (1HNMR, 13CNMR, H, H COSY, HMQC, HSQC, HMBC) spectral data of Rubrofusarin B (2). [file 2191-2858-2-6-S2.DOC]

**2. Additional file 2**

**Title:** Spectral data of Rubrofusarin B (**2**)

**Description:** Thirteen charts (chart 11- 23) containing the mass (ESI, EI, HRESI MS) and NMR (^1^HNMR, ^13^CNMR, H,H COSY, HMQC, HSQC, HMBC) spectral data of Rubrofusarin B (**2**).

**
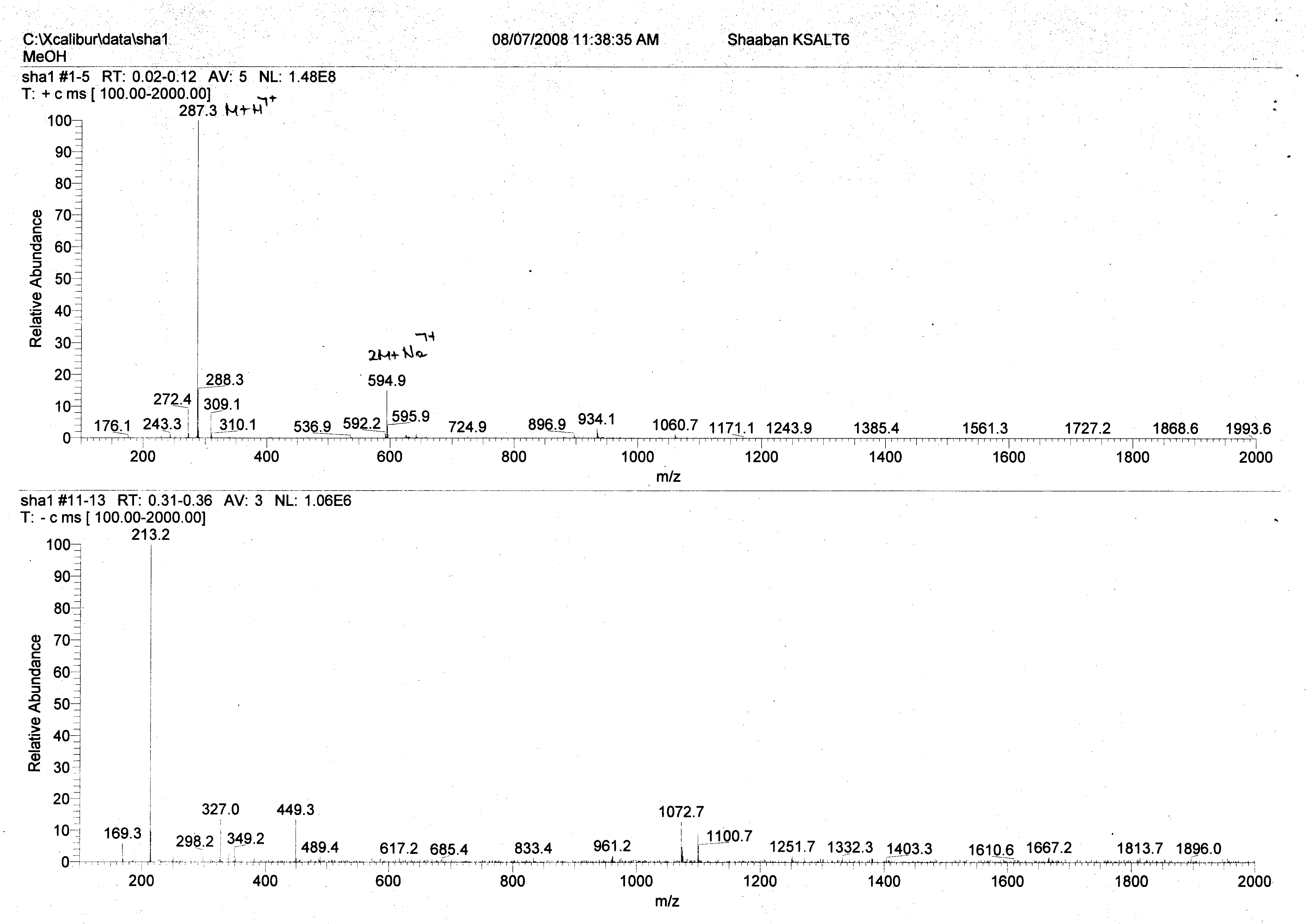
**

**Chart 11:** (+)-ESI-MS and (-)-ESI-MS spectrum of Rubrofusarin B (**2**)

**
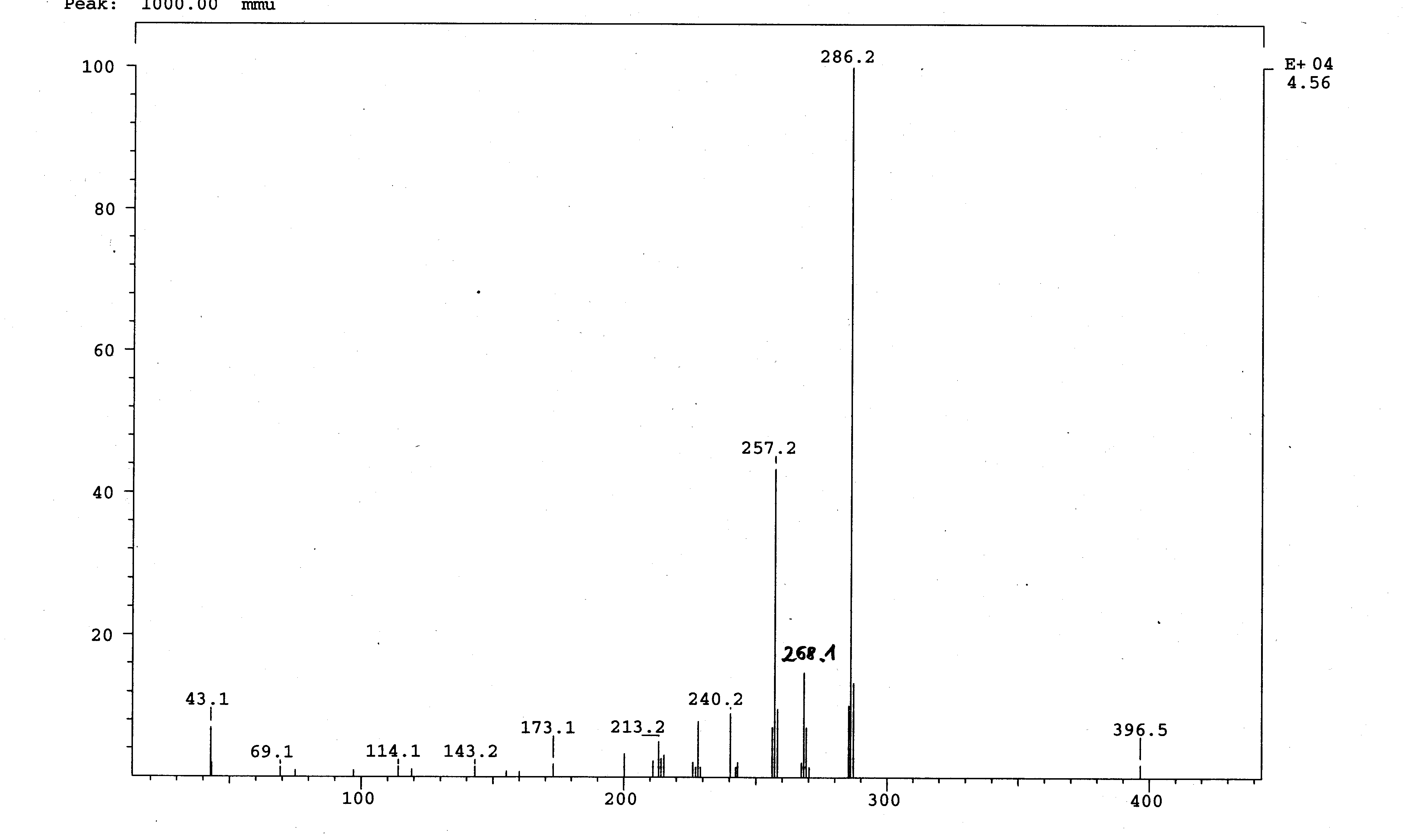
**

**Chart 12:** EI-MS spectrum of Rubrofusarin B (**2**)

**
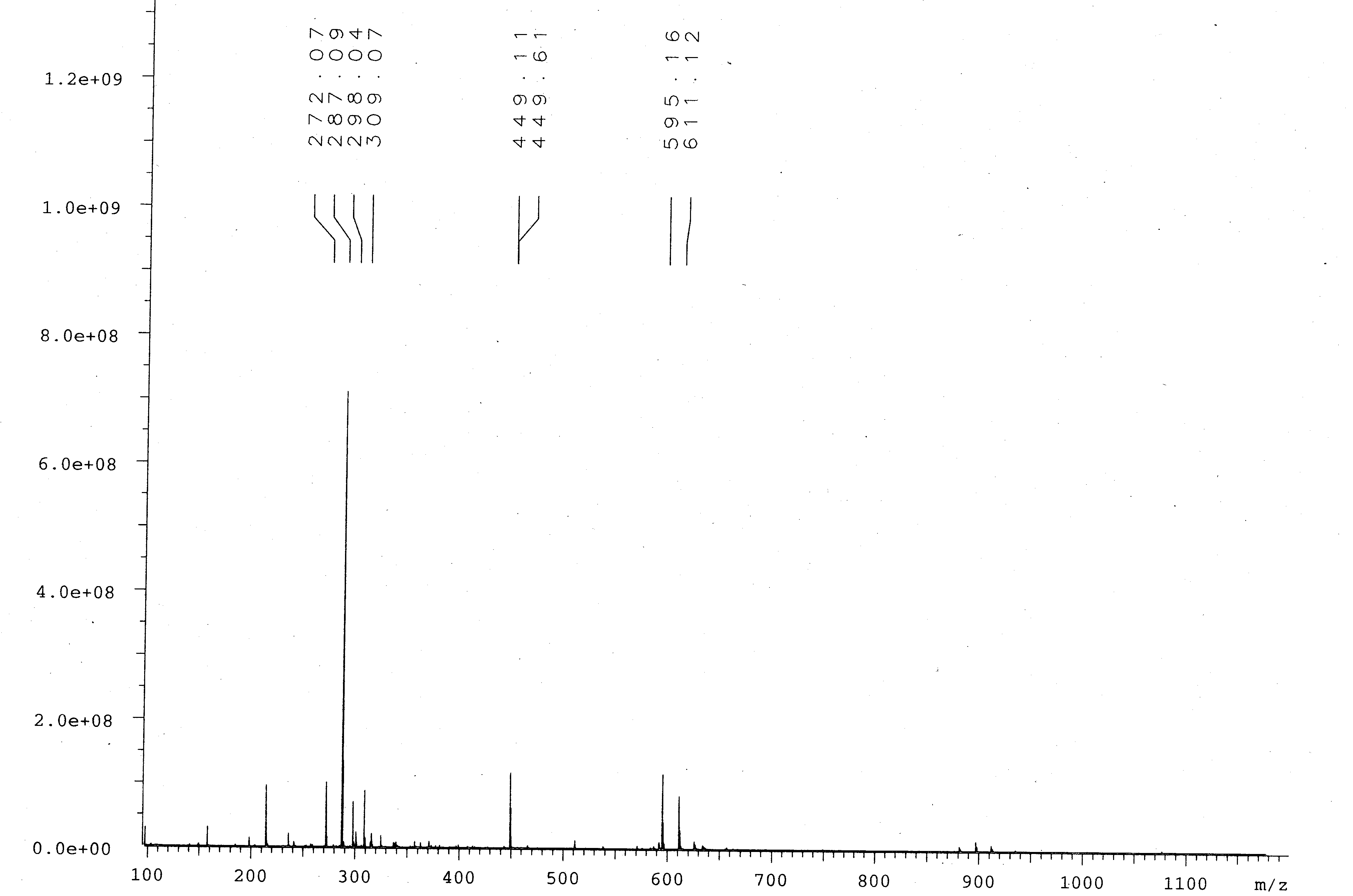
**

**Chart 13:** (+)-HRESI-MS spectrum of Rubrofusarin B (**2**)

**
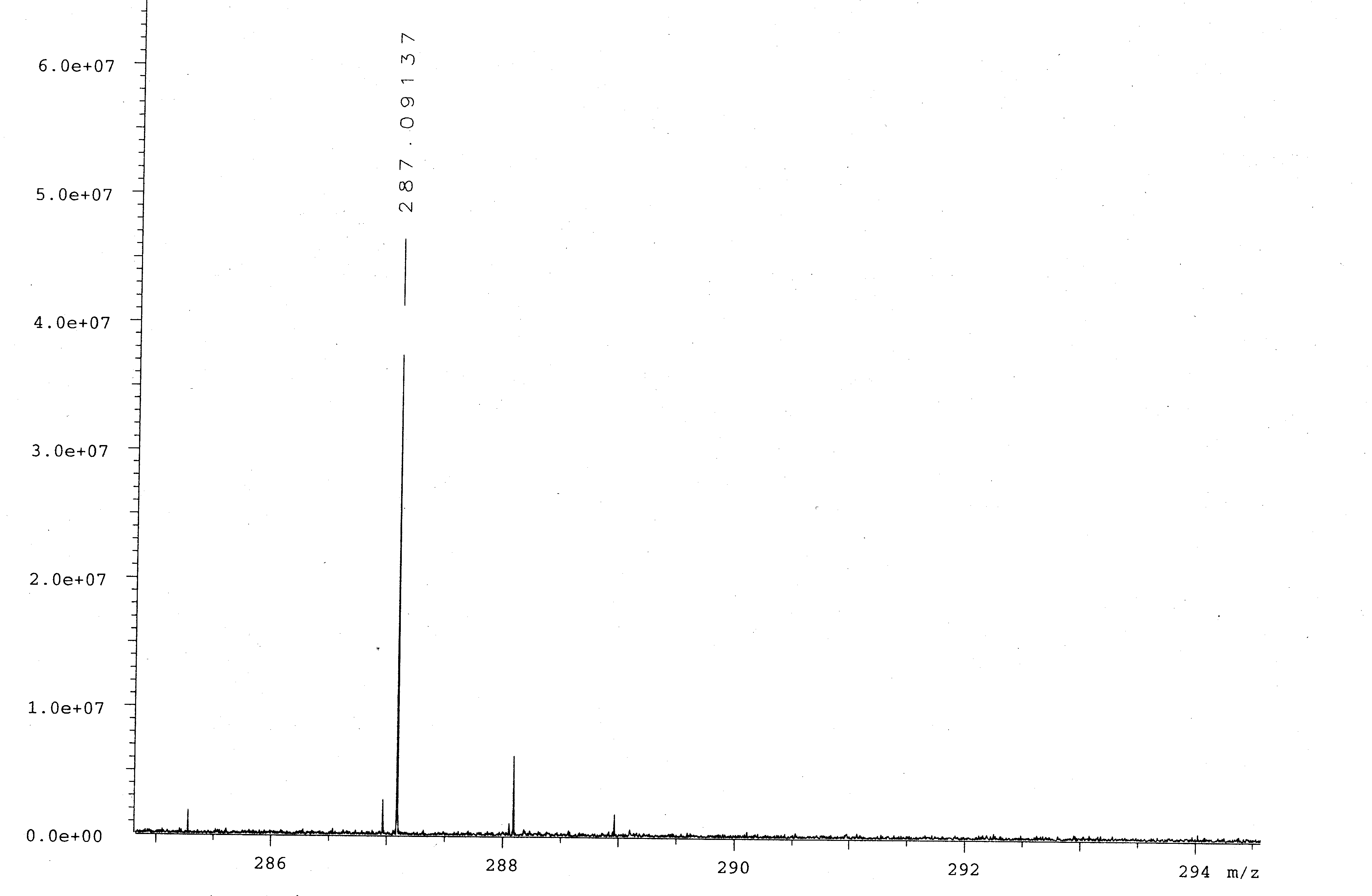
**

**Chart 14:** (+)-HRESI-MS expansion spectrum of Rubrofusarin B (**2**)

**
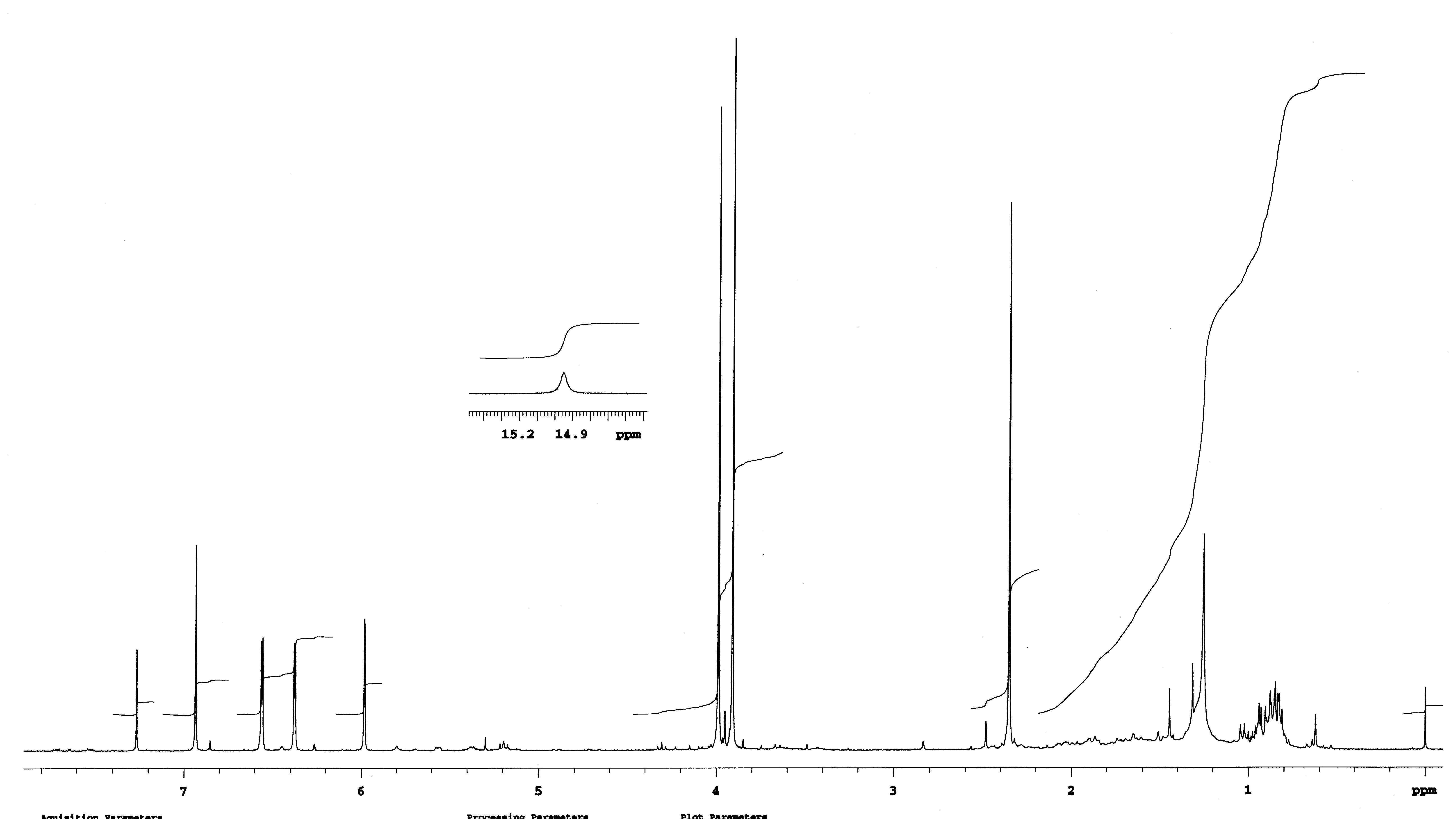
**

**Chart 15:** ^1^H NMR spectrum (CDCl_3_, 300 MHz) of Rubrofusarin B (**2**)

**
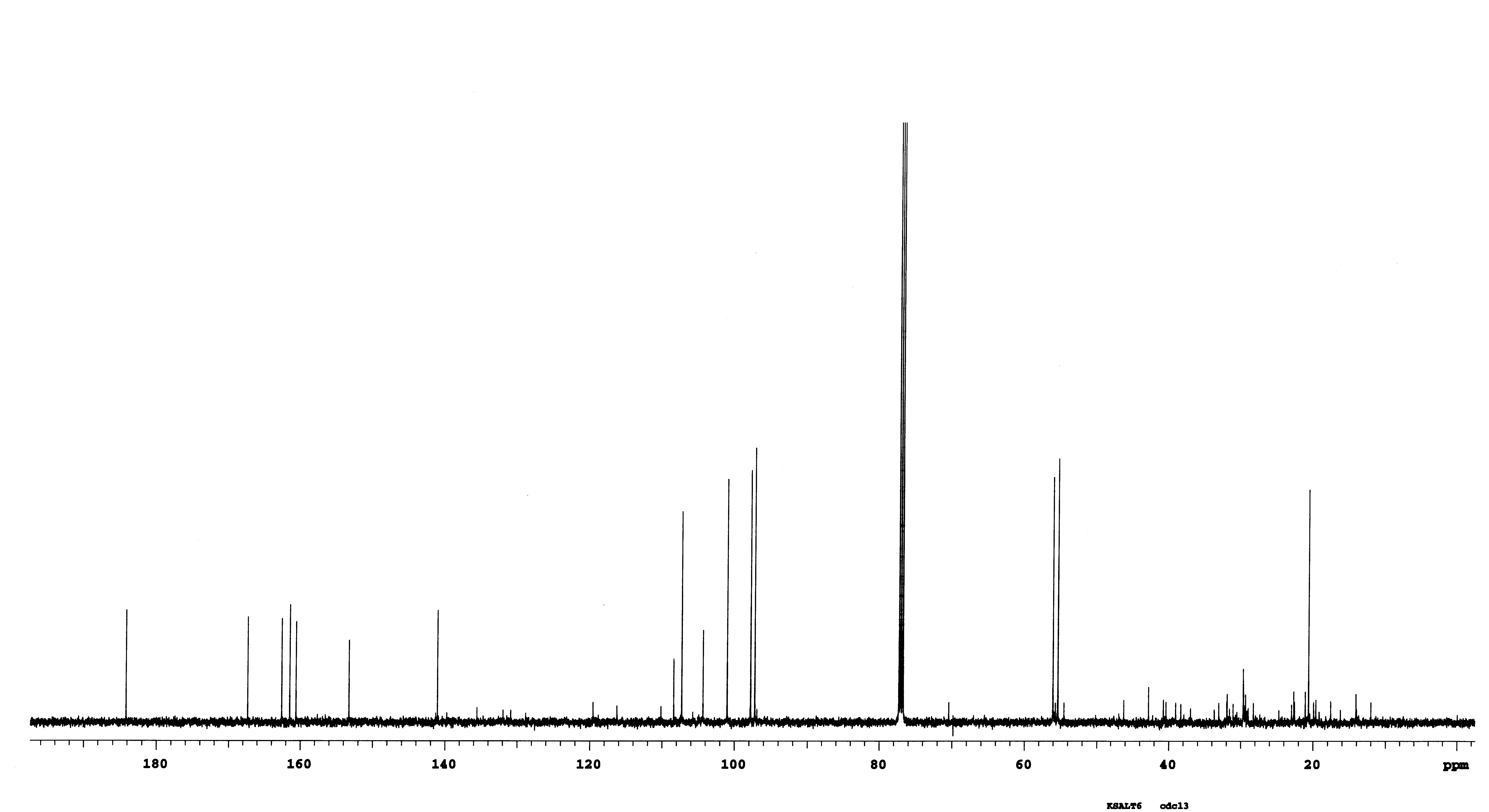
**

**Chart 16:** ^13^C NMR spectrum (CDCl_3_, 75 MHz) of Rubrofusarin B (**2**)

**
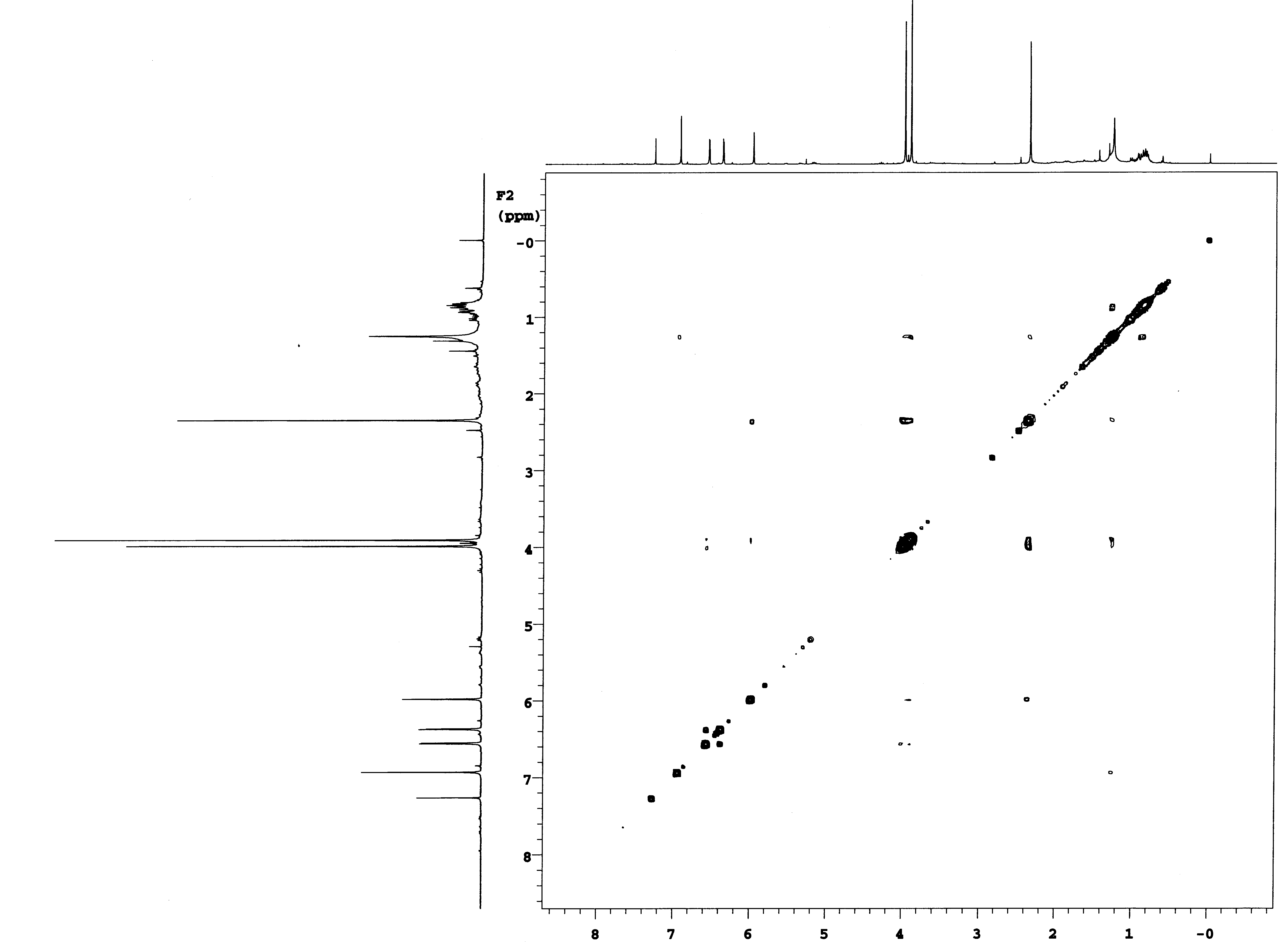
**

**Chart 17:** H,H COSY spectrum (CDCl_3_, 300 MHz) of Rubrofusarin B (**2**)

**
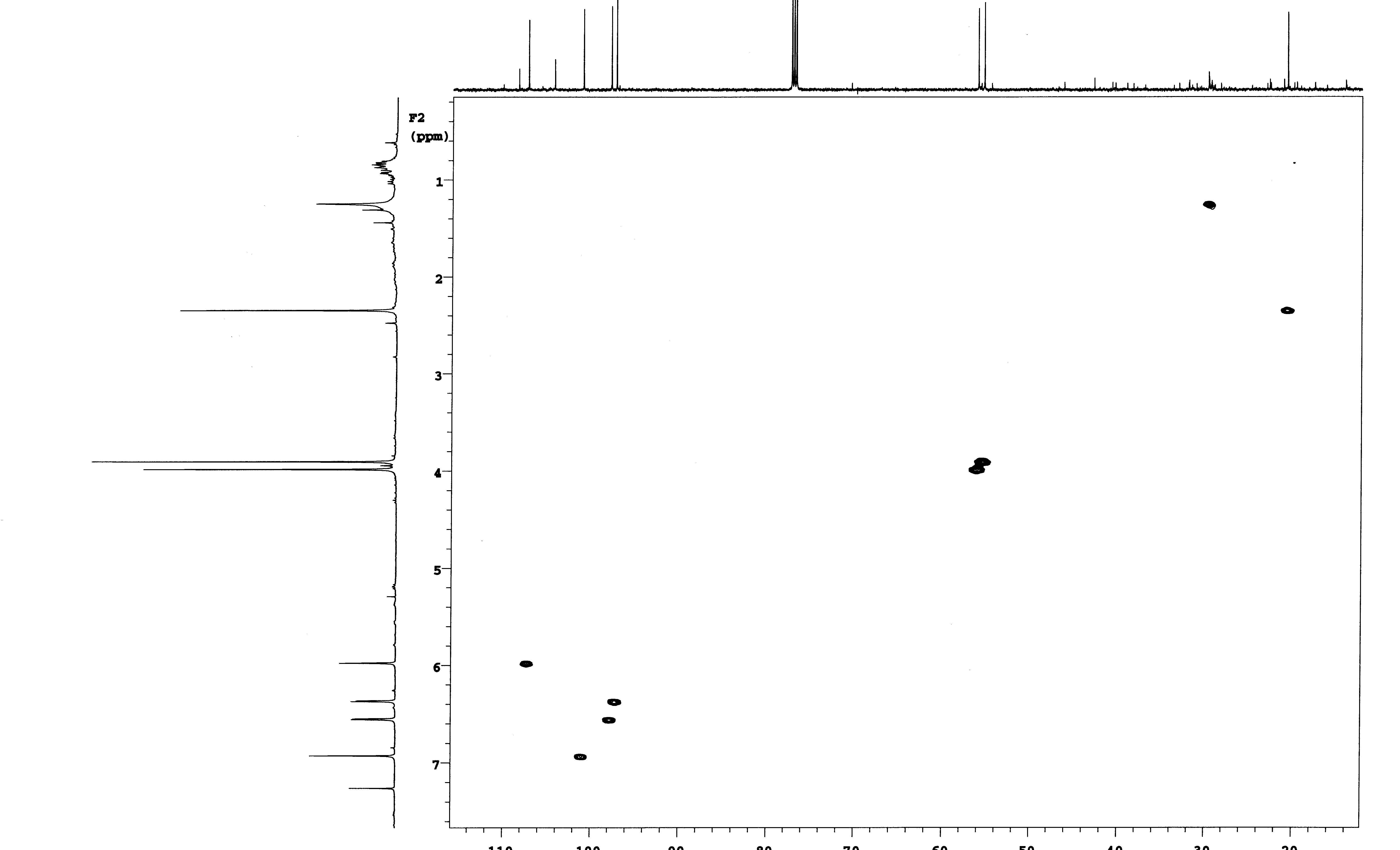
**

**Chart 18:** HMQC spectrum (CDCl_3_, 300 MHz) of Rubrofusarin B (**2**)

**
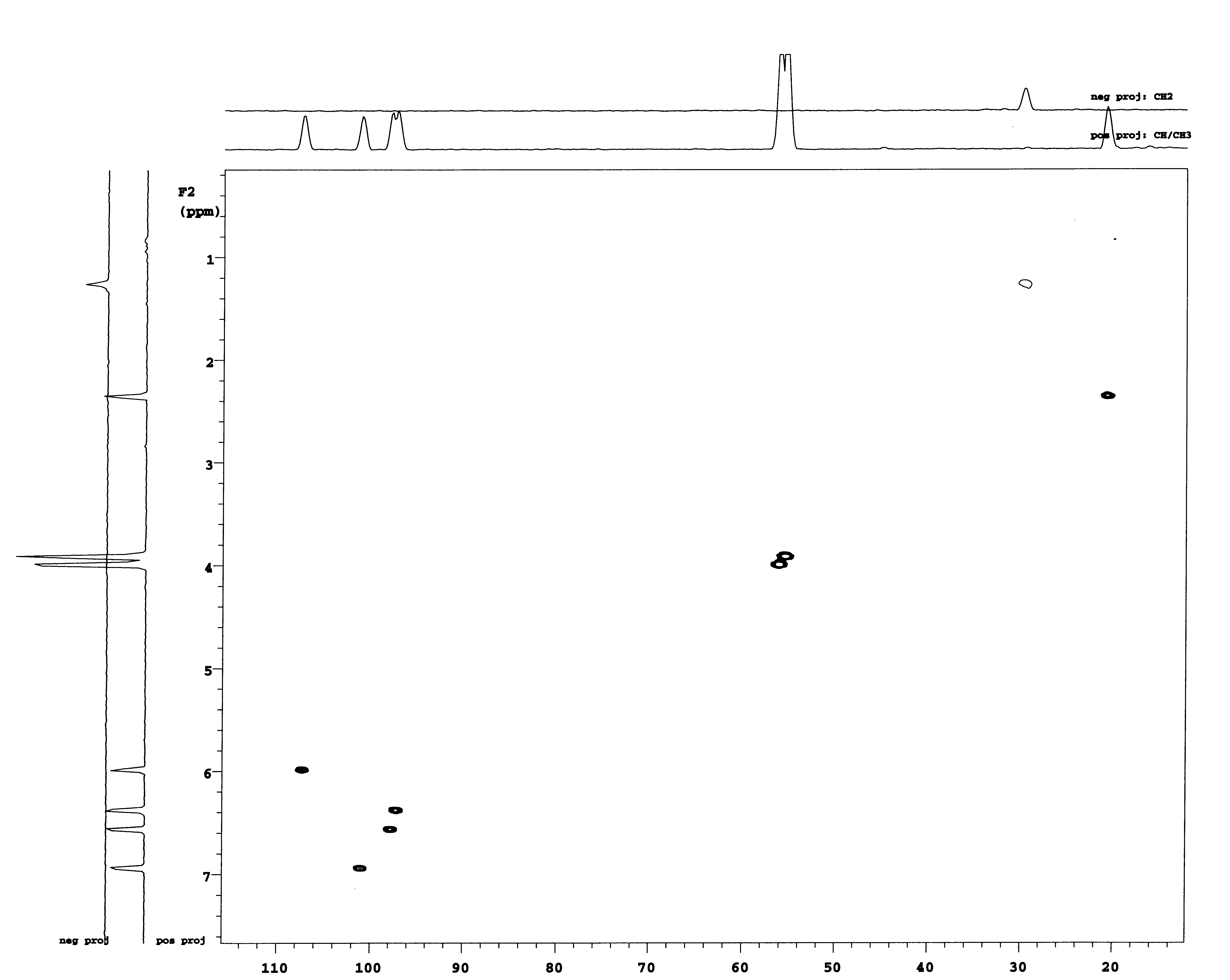
**

**Chart 19:** HSQC spectrum (CDCl_3_, 300 MHz) of Rubrofusarin B (**2**)

**
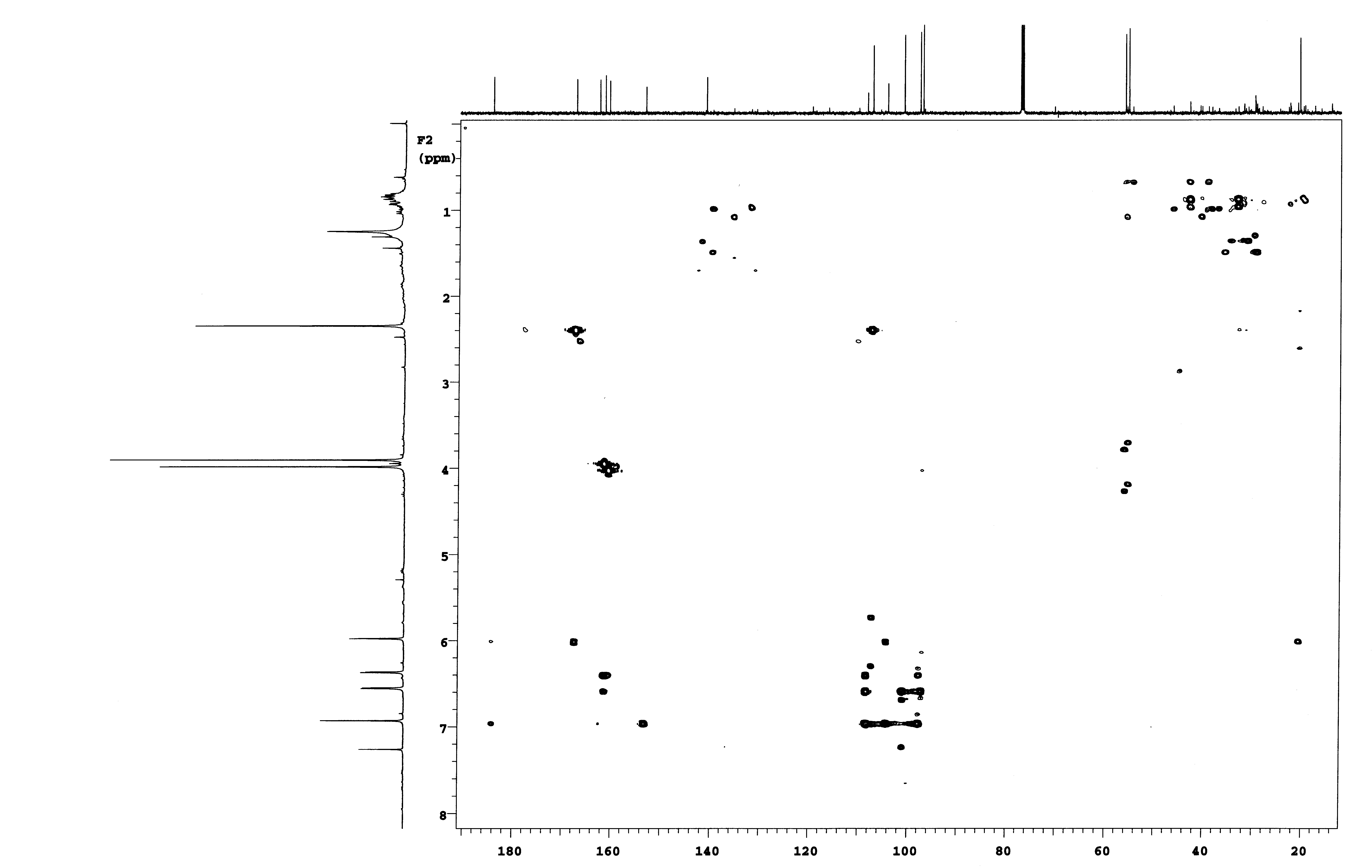
**

**Chart 20:** HMBC spectrum (CDCl_3_, 300 MHz) of Rubrofusarin B (**2**)

**
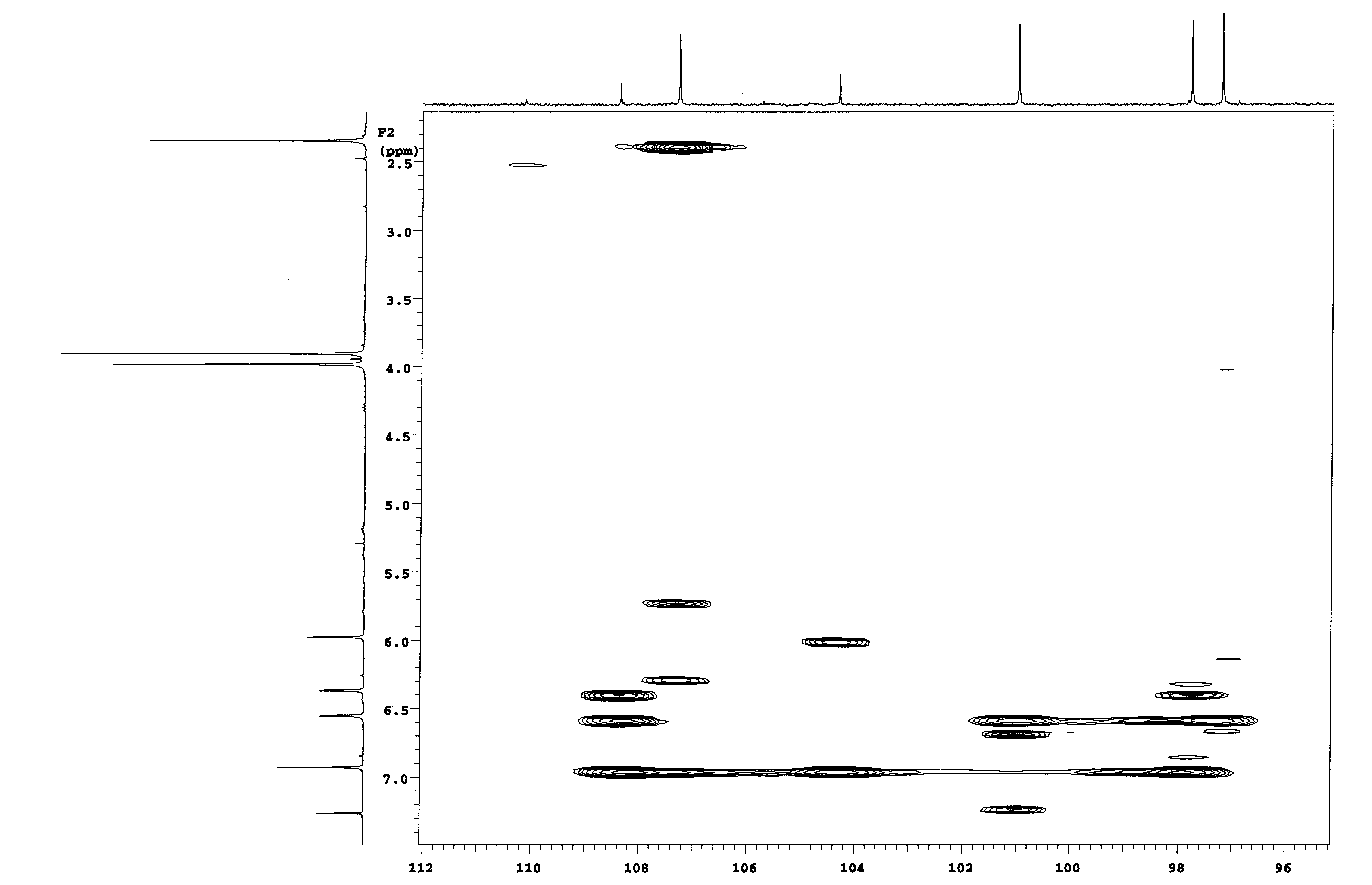
**

**Chart 21:** HMBC expansion spectrum (CDCl_3_, 300 MHz) of Rubrofusarin B (**2**)

**
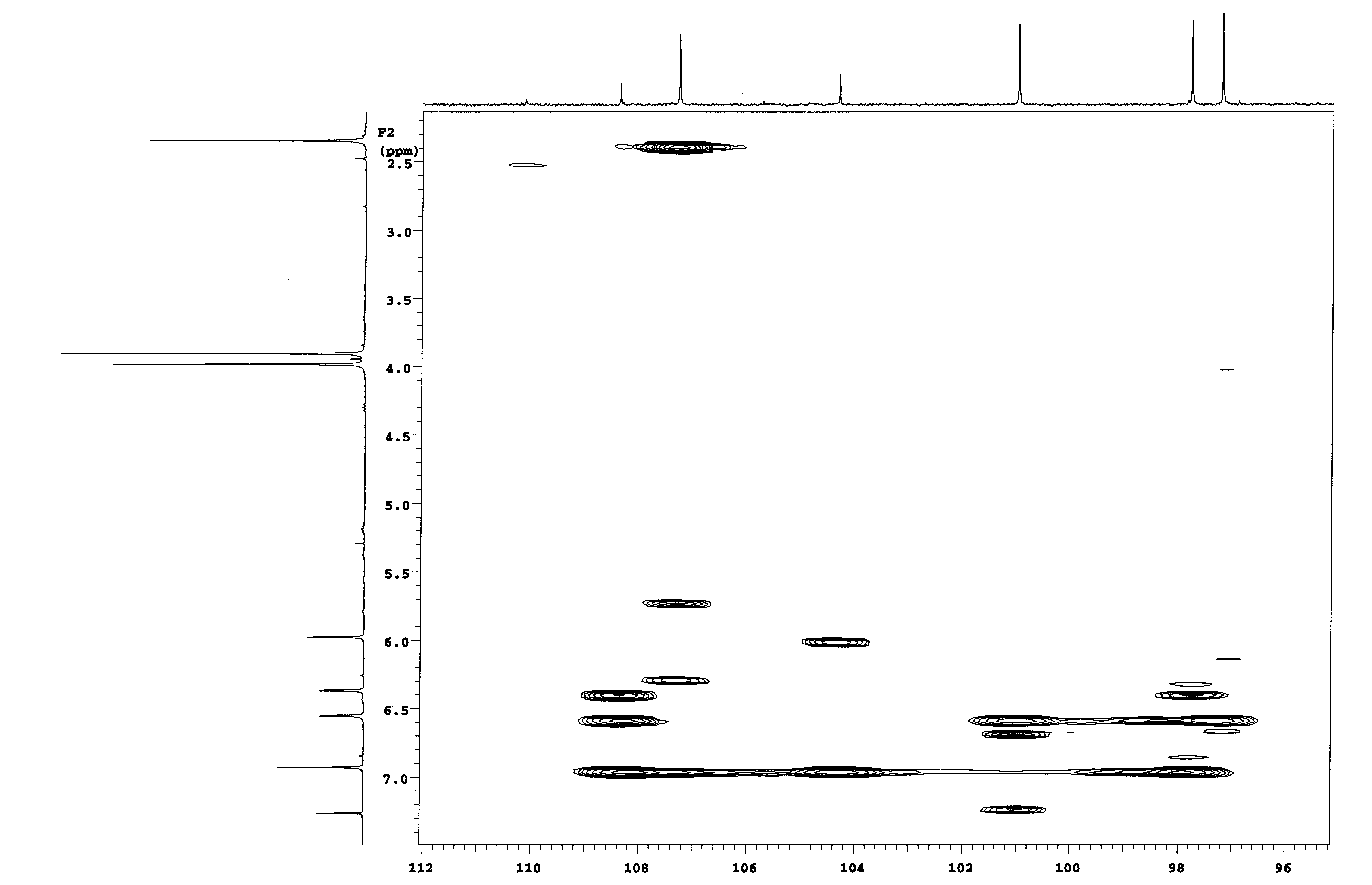
**

**Chart 22:** HMBC expansion spectrum (CDCl_3_, 300 MHz) of Rubrofusarin B (**2**)

**
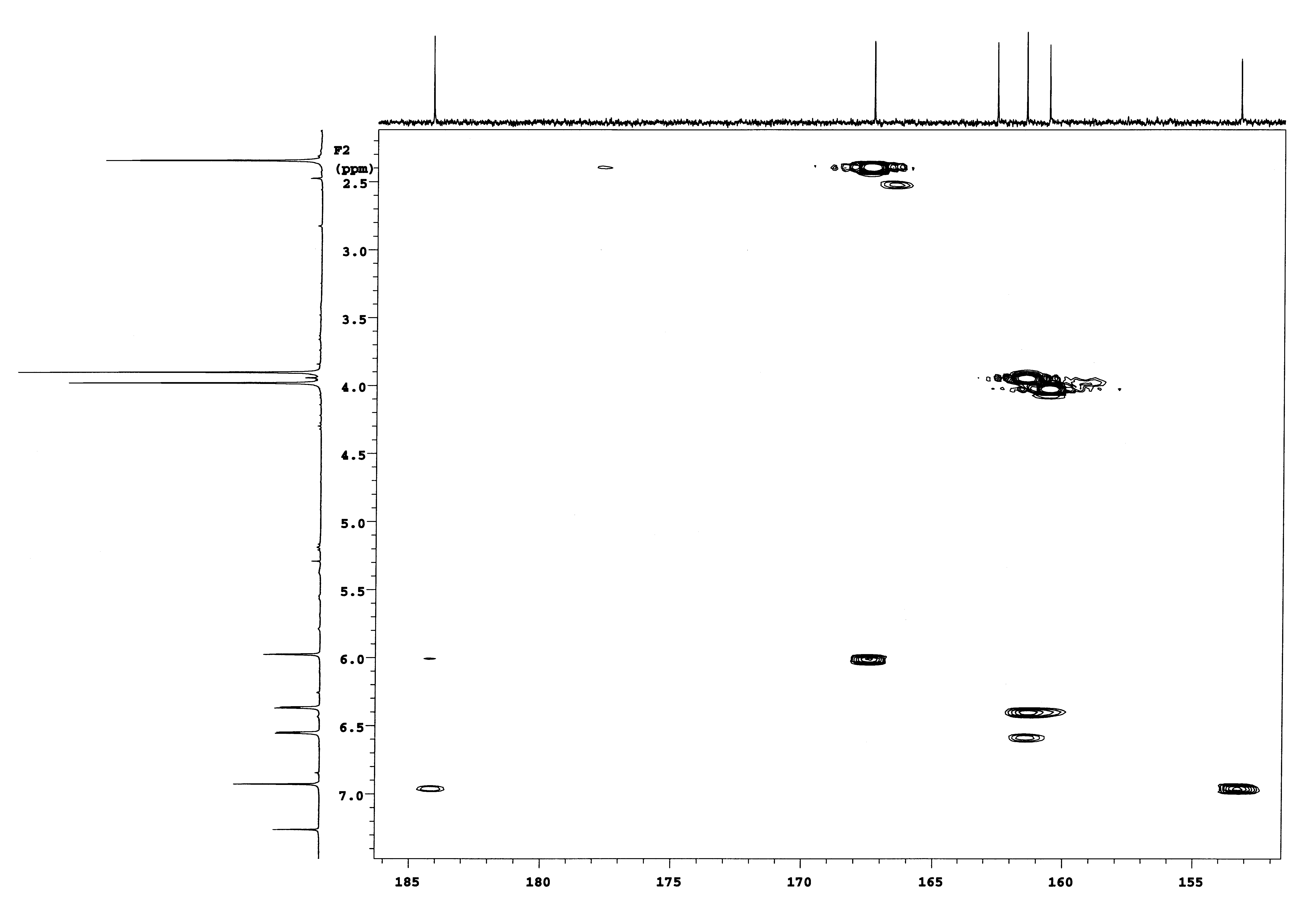
**

**Chart 23:** HMBC expansion spectrum (CDCl_3_, 300 MHz) of Rubrofusarin B (**2**)
