# Supplementary material for: Seven naphtho-γ-pyrones from the marine-derived fungus Alternaria alternata: structure elucidation and biological properties
Source: Org Med Chem Lett. 2012 Feb 29;2:6. doi: 10.1186/2191-2858-2-6 (PMC3350997; doi:10.1186/2191-2858-2-6)
Supplement: Additional file 3 — Spectral data of Fonsecin (3). Nine charts (chart 24-32) containing the mass (ESI, EI MS) and NMR (1HNMR, 13CNMR, H, H COSY, HMQC, HSQC, HMBC) spectral data of Fonsecin (3). [file 2191-2858-2-6-S3.DOC]

**3. Additional file 3**

**Title:** Spectral data of Fonsecin (**3**)

**Description:** Nine charts (chart 24-32) containing the mass (ESI, EI MS) and NMR (^1^HNMR, ^13^CNMR, H,H COSY, HMQC, HSQC, HMBC) spectral data of Fonsecin (**3**).


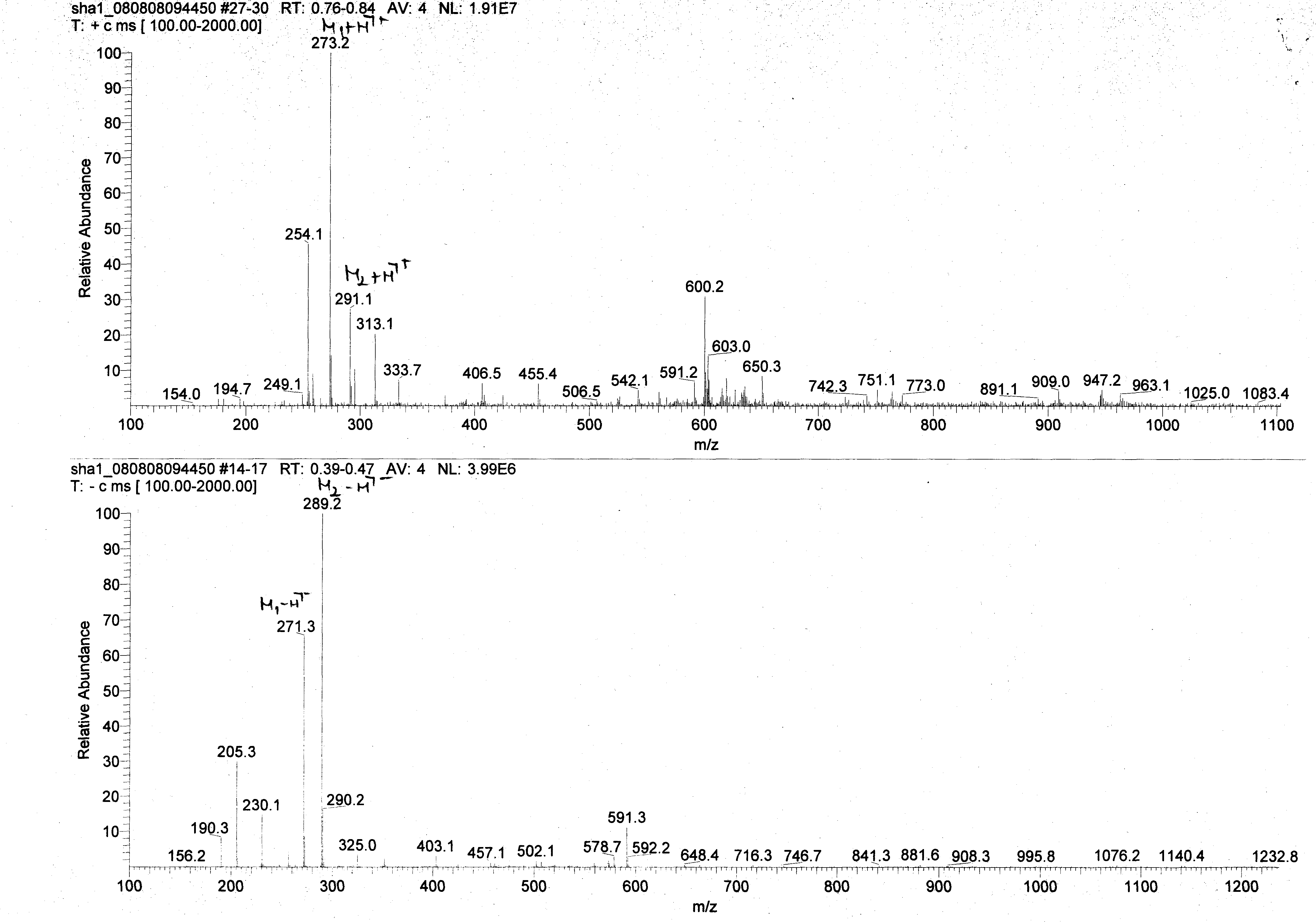


**Chart 24:** (+)-ESI-MS and (-)-ESI-MS spectra of Fonsecin (**3**)

**
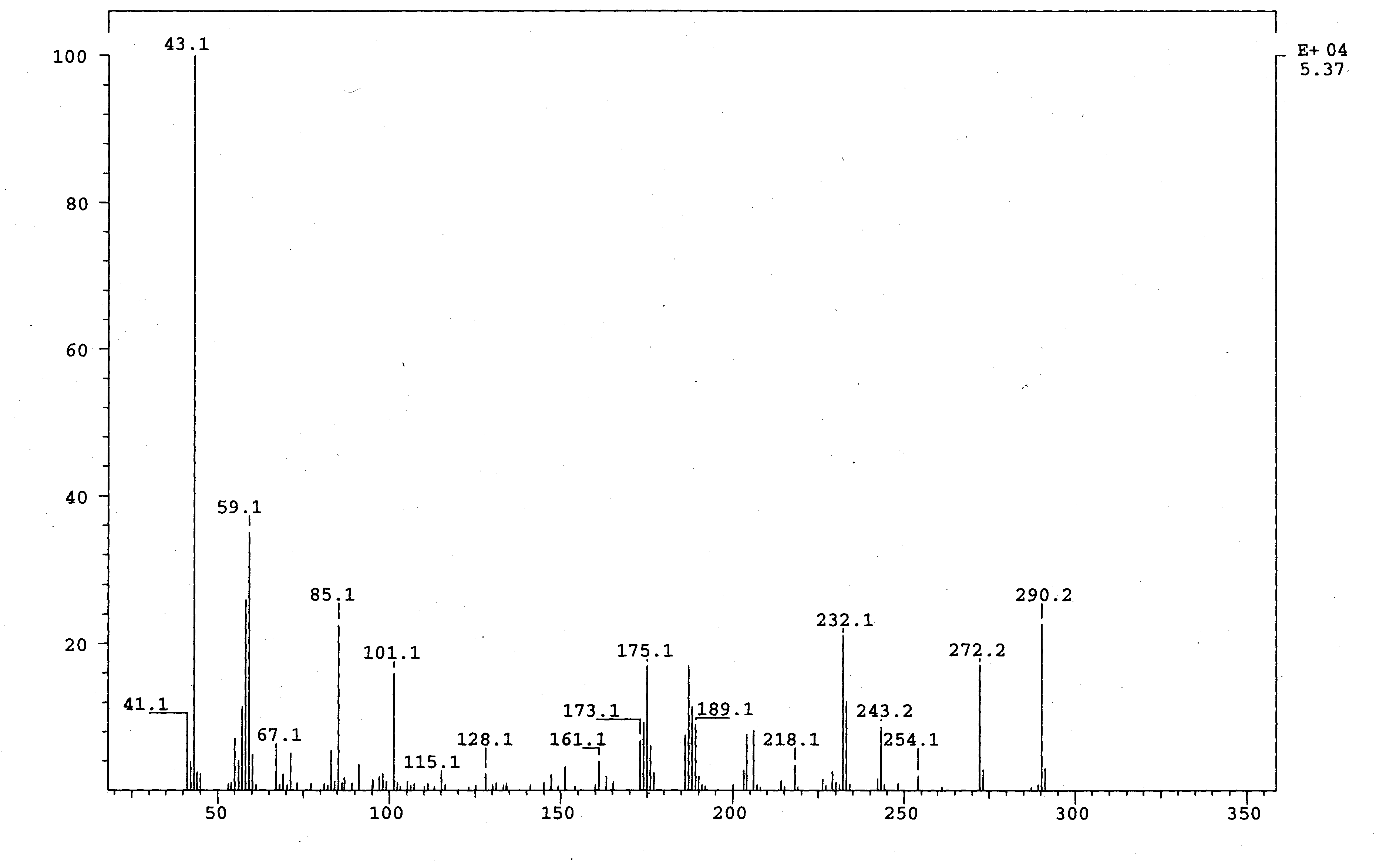
**

**Chart 25:** EI-MS spectrum of Fonsecin (**3**)

**
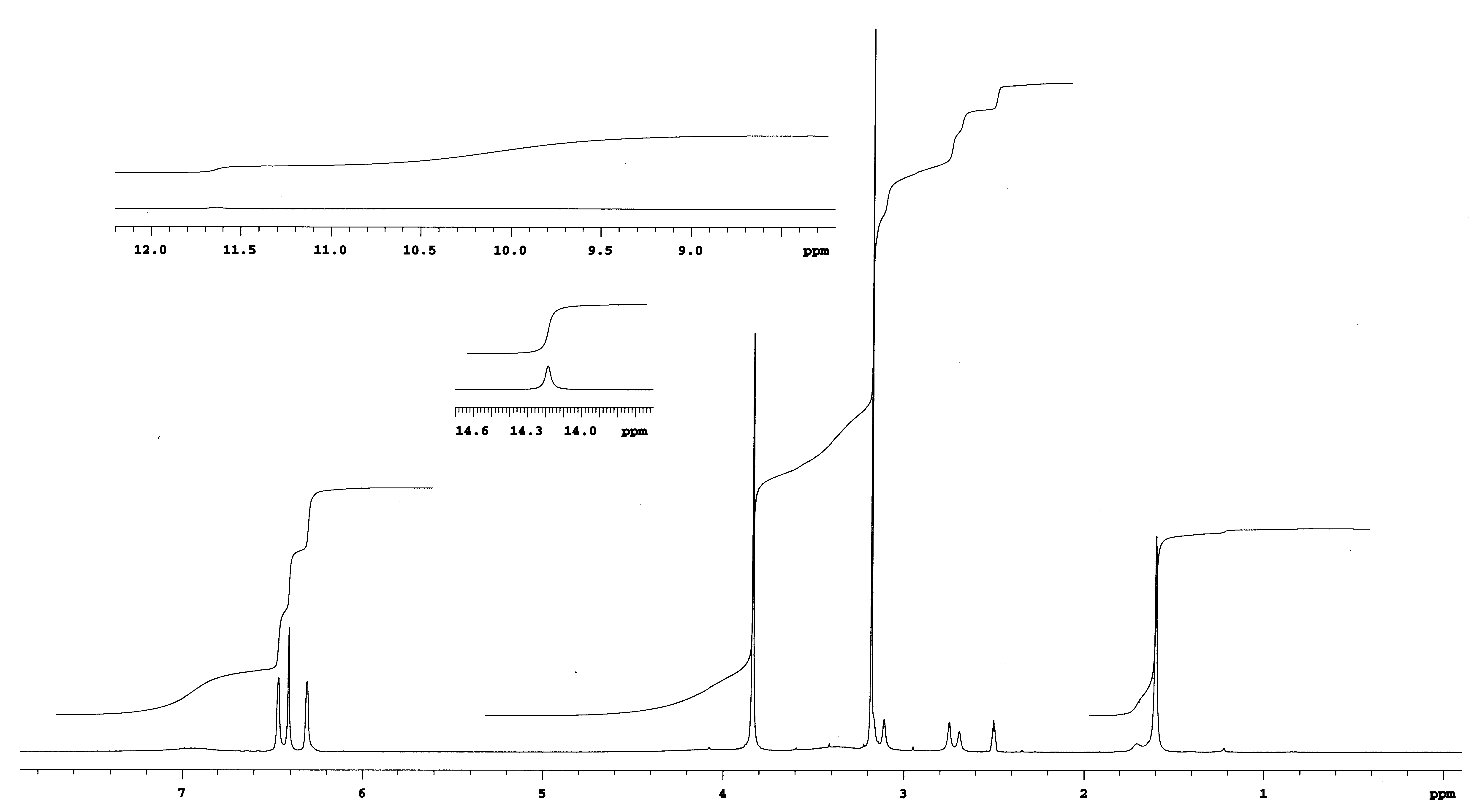
**

**Chart 26:** ^1^H NMR spectrum (DMSO-*d*_6_, 300 MHz) of Fonsecin (**3**)

**
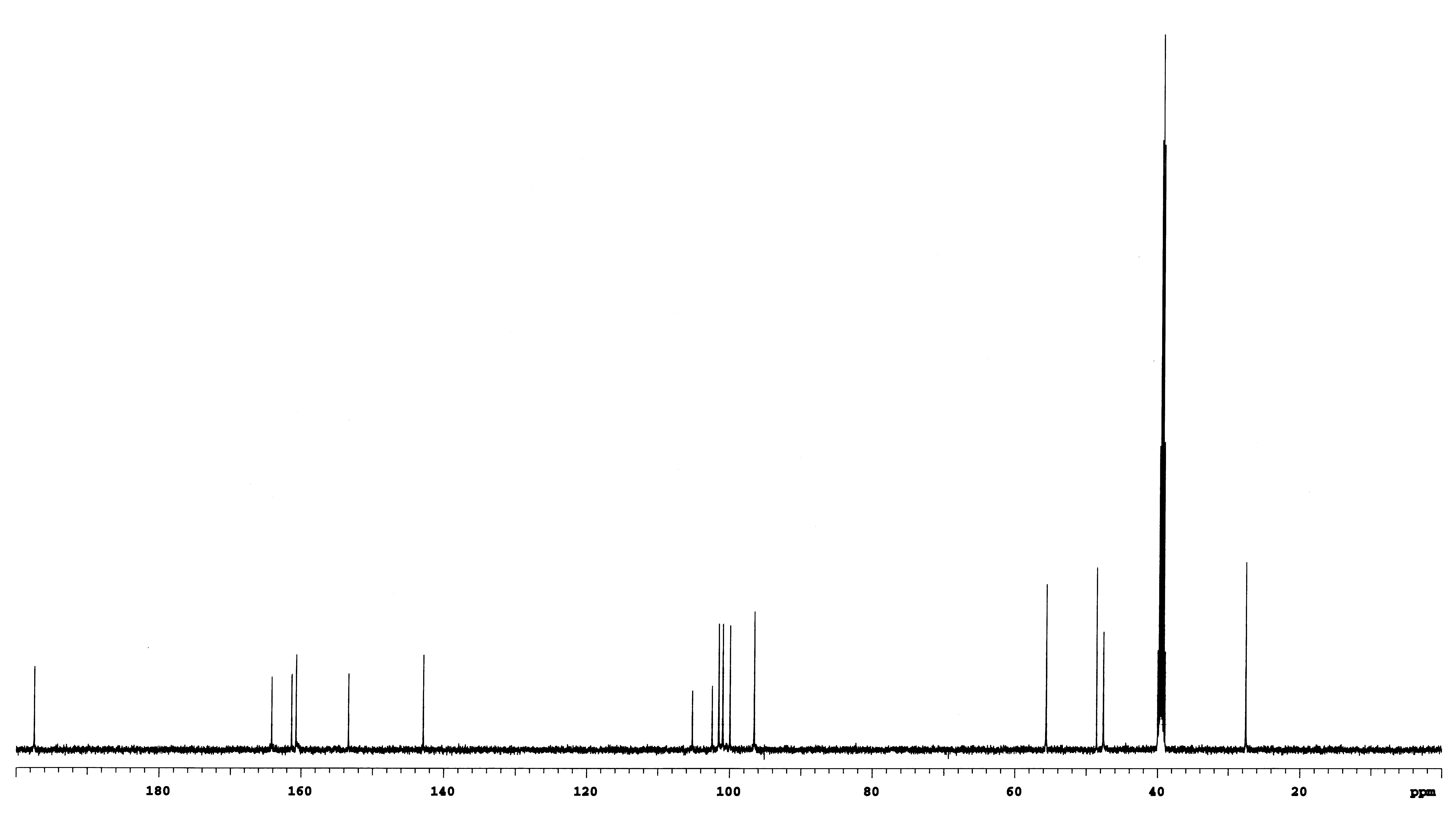
**

**Chart 27:** ^13^C NMR spectrum (DMSO-*d*_6_, 125 MHz) of Fonsecin (**3**)

**
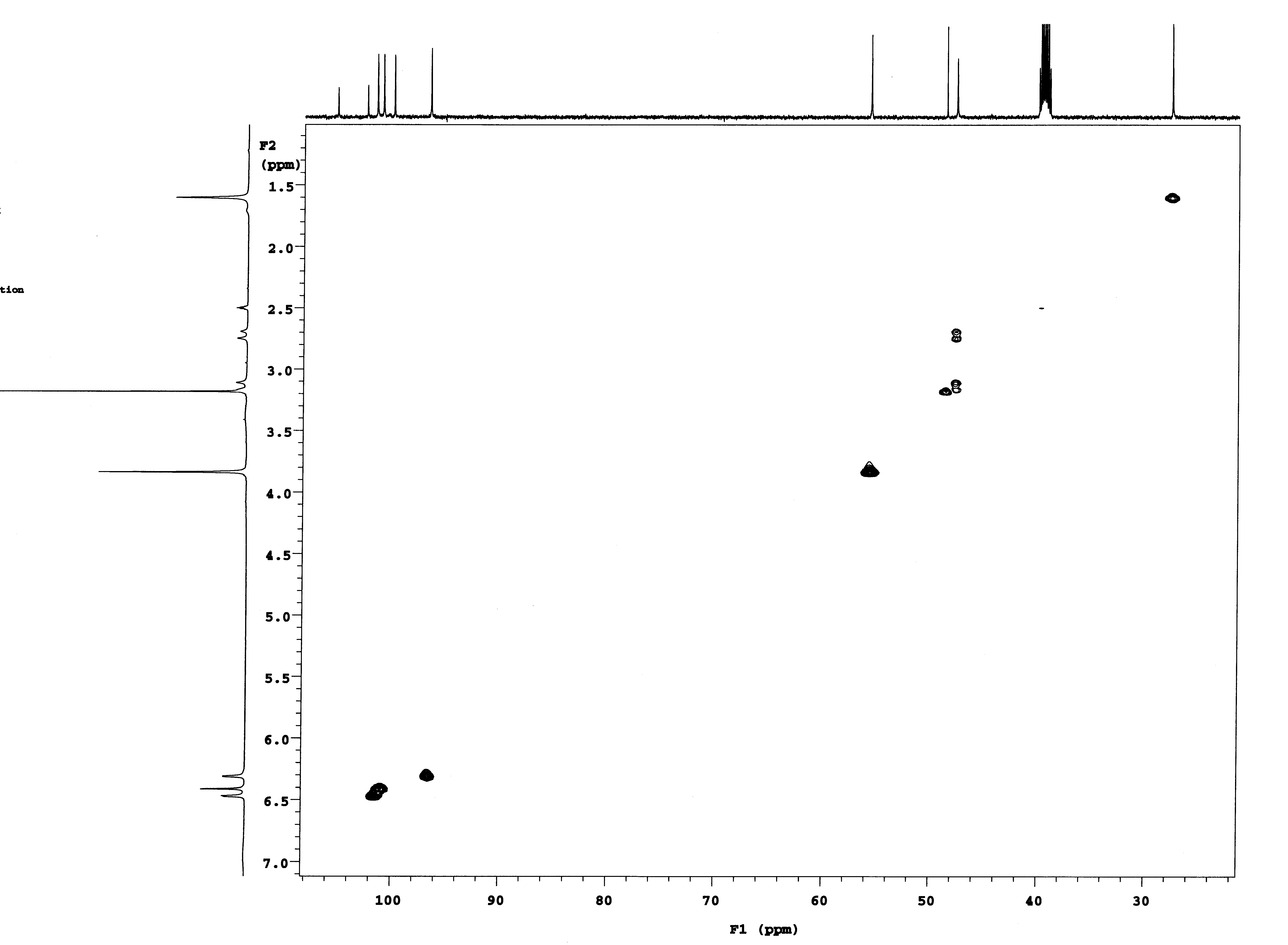
**

**Chart 28:** HMQC spectrum (DMSO-*d*_6_, 300 MHz) of Fonsecin (**3**)

**
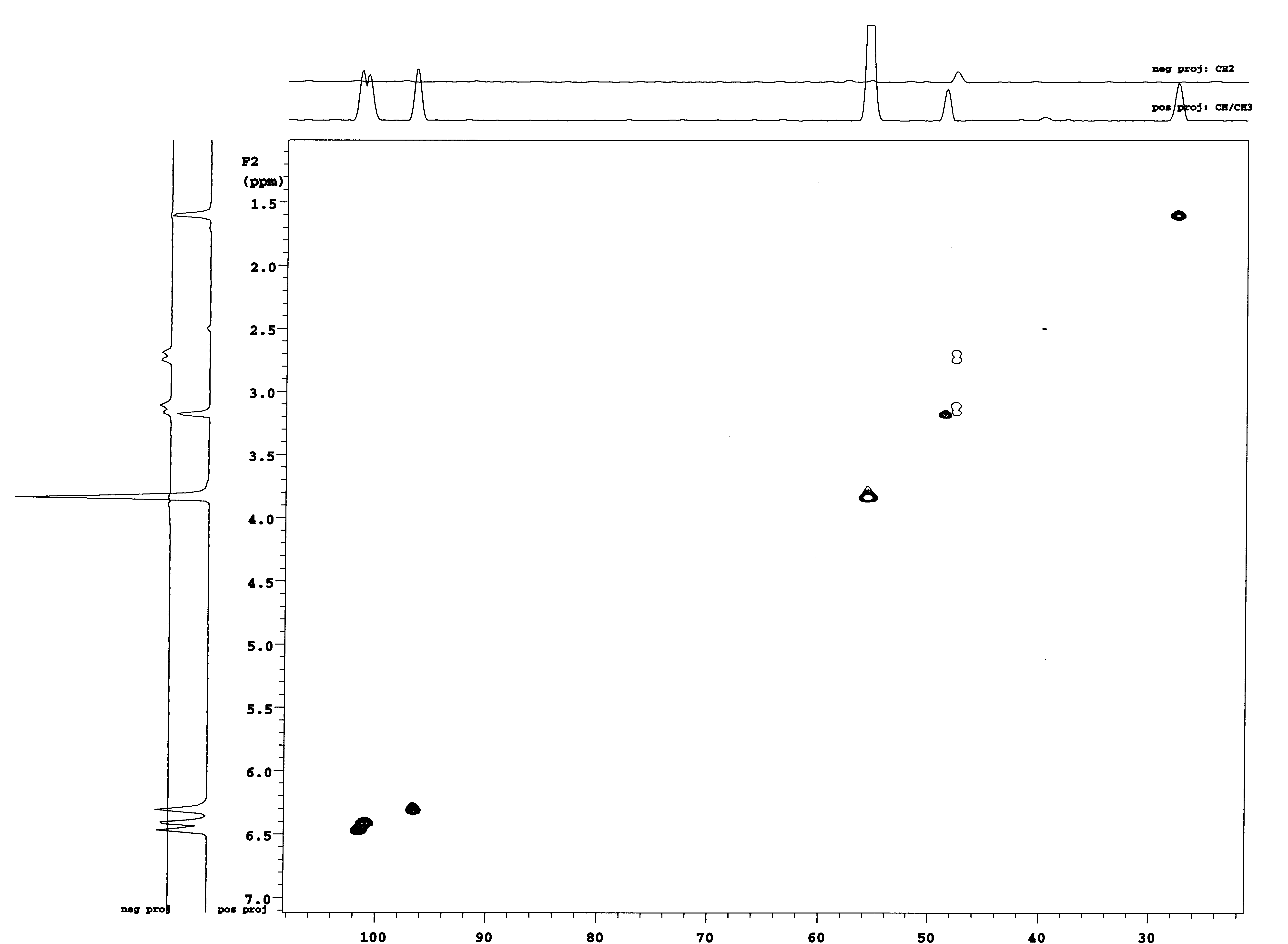
**

**Chart 29:** HSQC spectrum (DMSO-*d*_6_, 300 MHz) of Fonsecin (**3**)

**
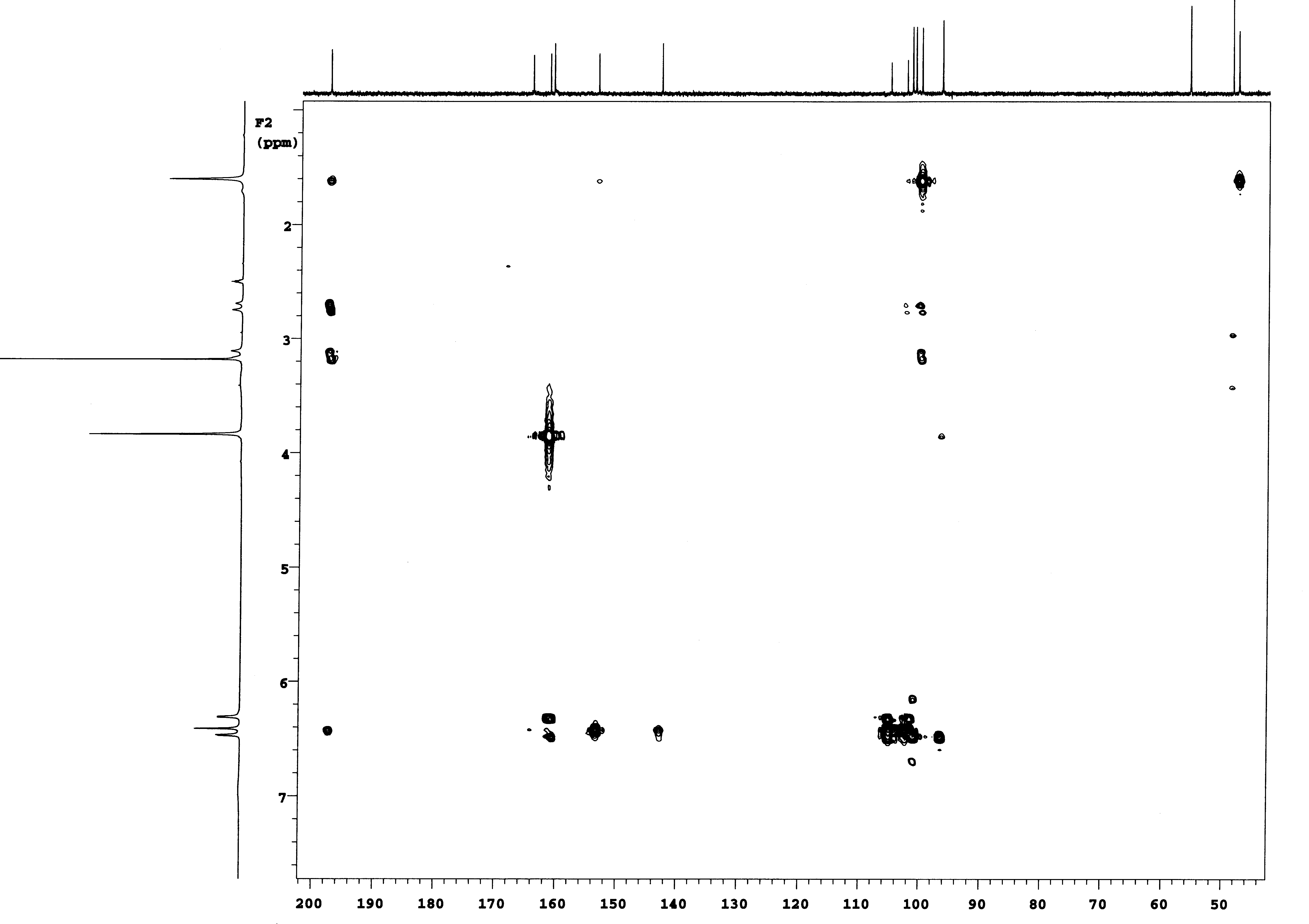
**

**Chart 30:** HMBC spectrum (DMSO-*d*_6_, 300 MHz) of Fonsecin (**3**)


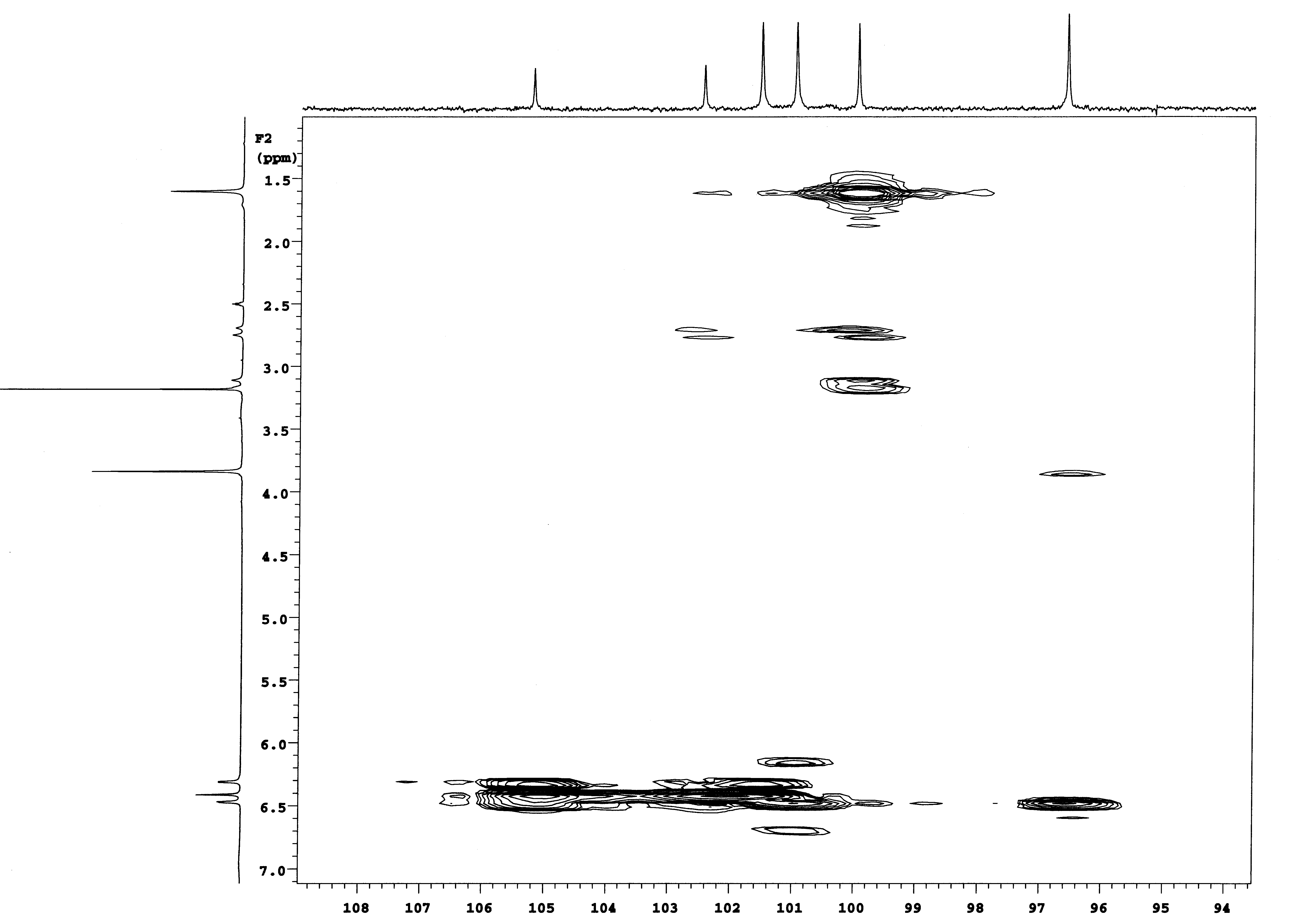


**Chart 31:** HMBC expansion spectrum (DMSO-*d*_6_, 300 MHz) of Fonsecin (**3**)


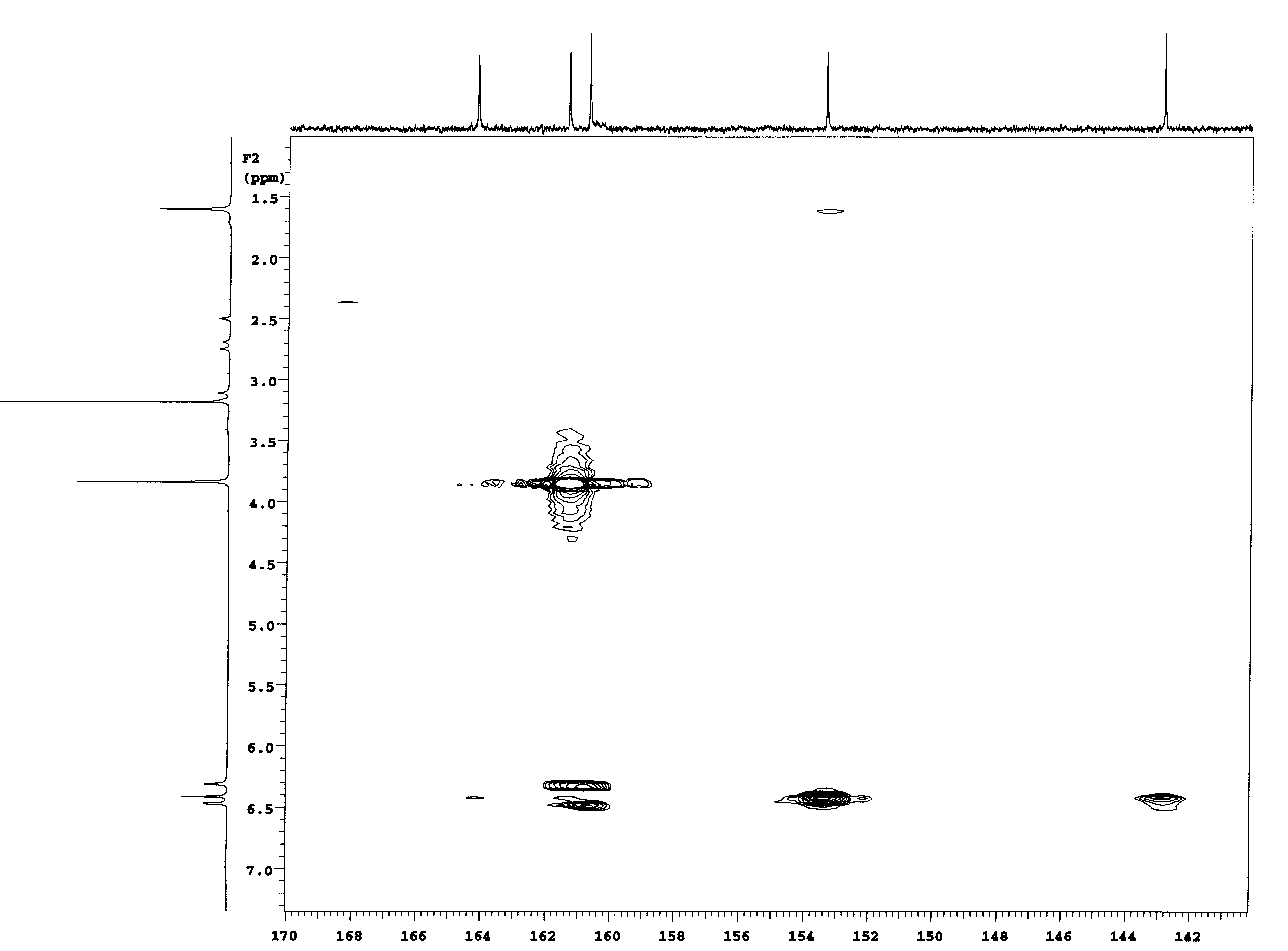


**Chart 32:** HMBC expansion spectrum (DMSO-*d*_6_, 300 MHz) of Fonsecin (**3**)
