# Supplementary material for: Seven naphtho-γ-pyrones from the marine-derived fungus Alternaria alternata: structure elucidation and biological properties
Source: Org Med Chem Lett. 2012 Feb 29;2:6. doi: 10.1186/2191-2858-2-6 (PMC3350997; doi:10.1186/2191-2858-2-6)
Supplement: Additional file 4 — Spectral data of Fonsecin B (5). Two charts (chart 33-34) containing the mass (EI MS) and NMR (1HNMR) spectral data of Fonsecin B (5). [file 2191-2858-2-6-S4.DOC]

**4. Additional file 4**

**Title:** Spectral data of Fonsecin B (**5**)

**Description:** Two charts (chart 33-34) containing the mass (EI MS) and NMR (^1^HNMR) spectral data of Fonsecin B (**5**).

**
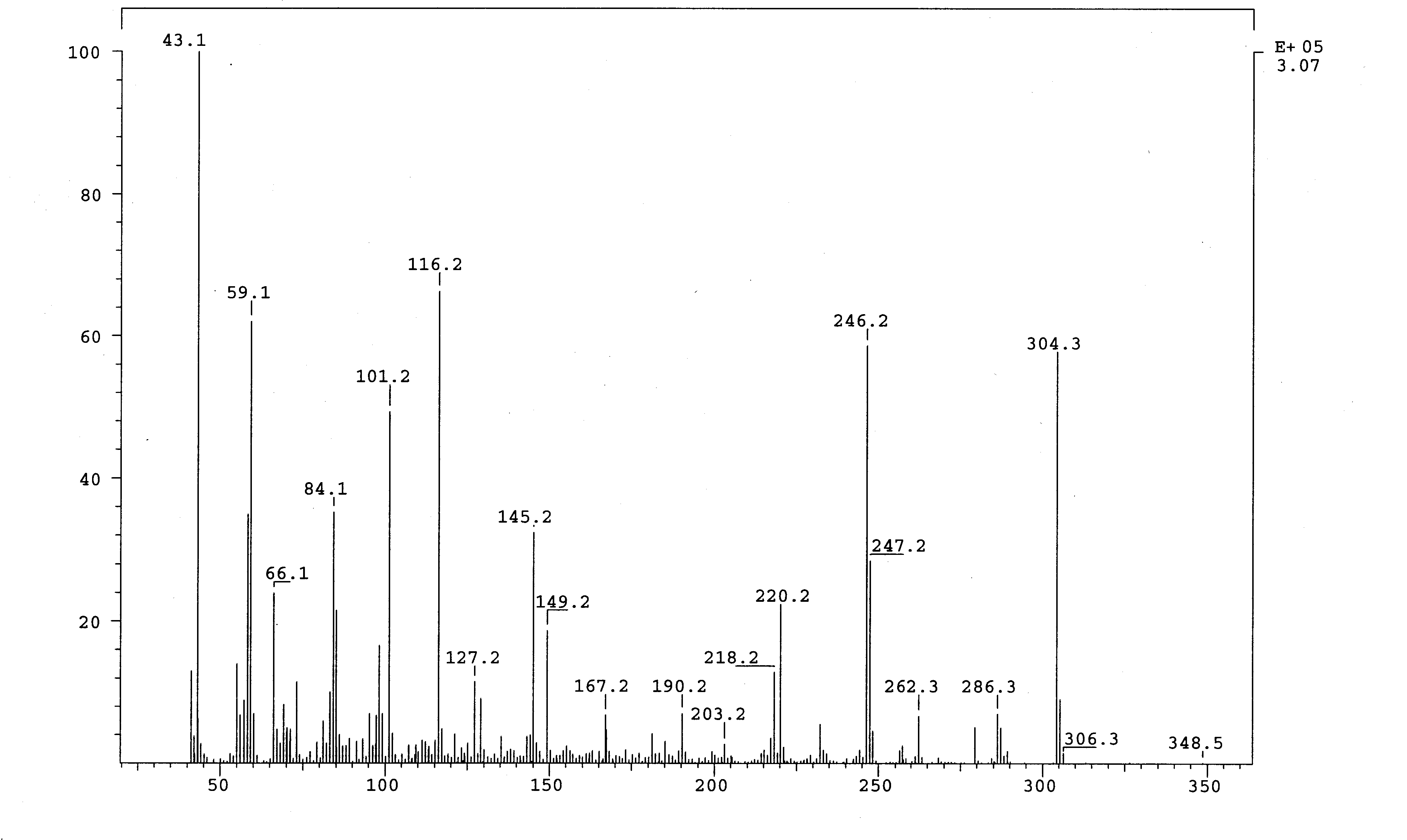
**

**Chart 33:** EI-MS spectrum of Fonsecin B (**5**)

**
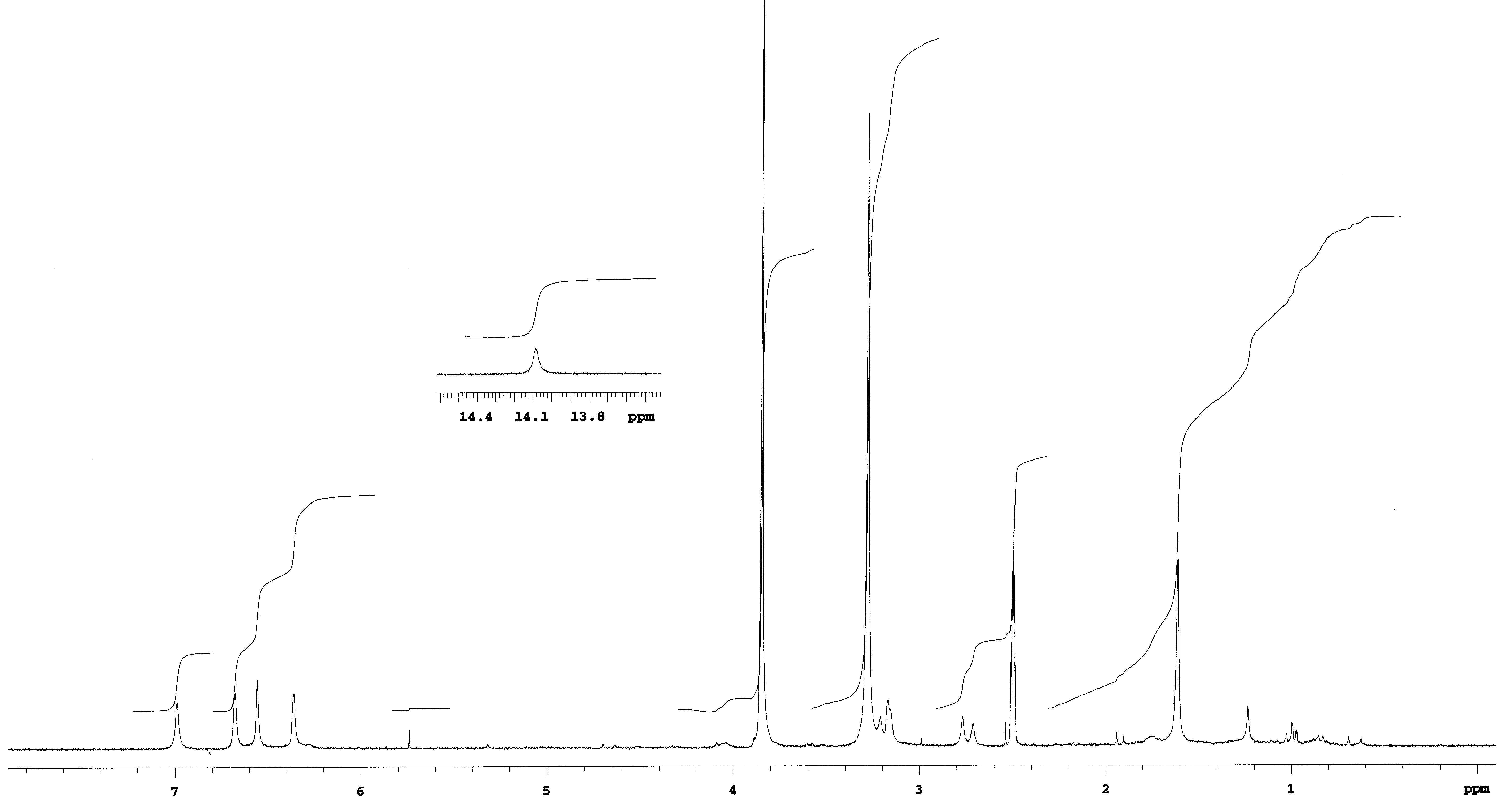
**

**Chart 34:** ^1^H NMR spectrum (DMSO-*d*_6_, 300 MHz) of Fonsecin B (**5**)
