# Supplementary material for: Seven naphtho-γ-pyrones from the marine-derived fungus Alternaria alternata: structure elucidation and biological properties
Source: Org Med Chem Lett. 2012 Feb 29;2:6. doi: 10.1186/2191-2858-2-6 (PMC3350997; doi:10.1186/2191-2858-2-6)
Supplement: Additional file 5 — Spectral data of Aurasperone A (6). Two charts (chart 35-36) containing the mass (EI MS) and NMR (1HNMR) spectral data of Aurasperone A (6) [file 2191-2858-2-6-S5.DOC]

**5. Additional file 5**

**Title:** Spectral data of Aurasperone A (**6**)

**Description:** Two charts (chart 35-36) containing the mass (EI MS) and NMR (^1^HNMR) spectral data of Aurasperone A (**6**)


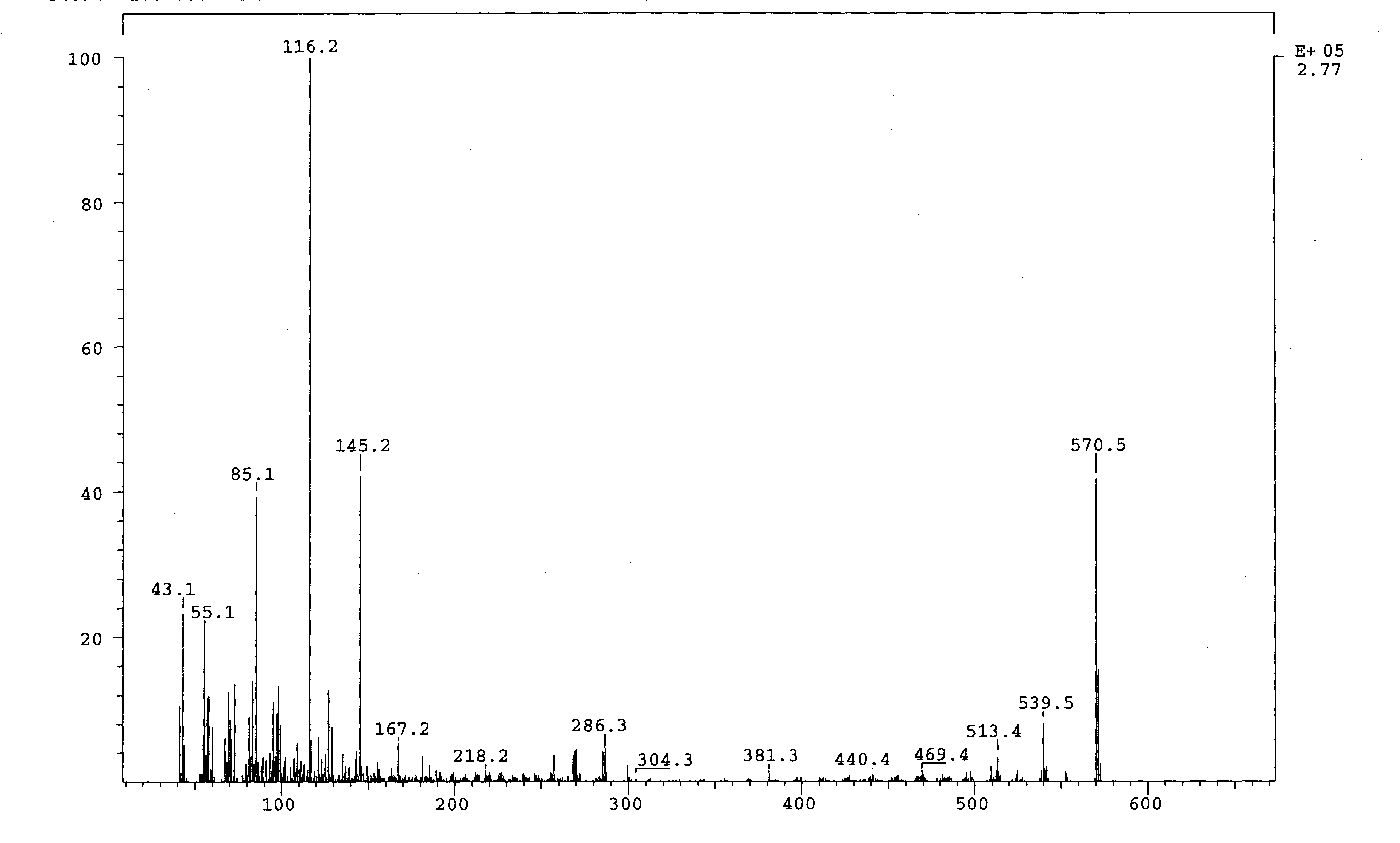


**Chart 35:** EI-MS spectrum of Aurasperone A (**6**)


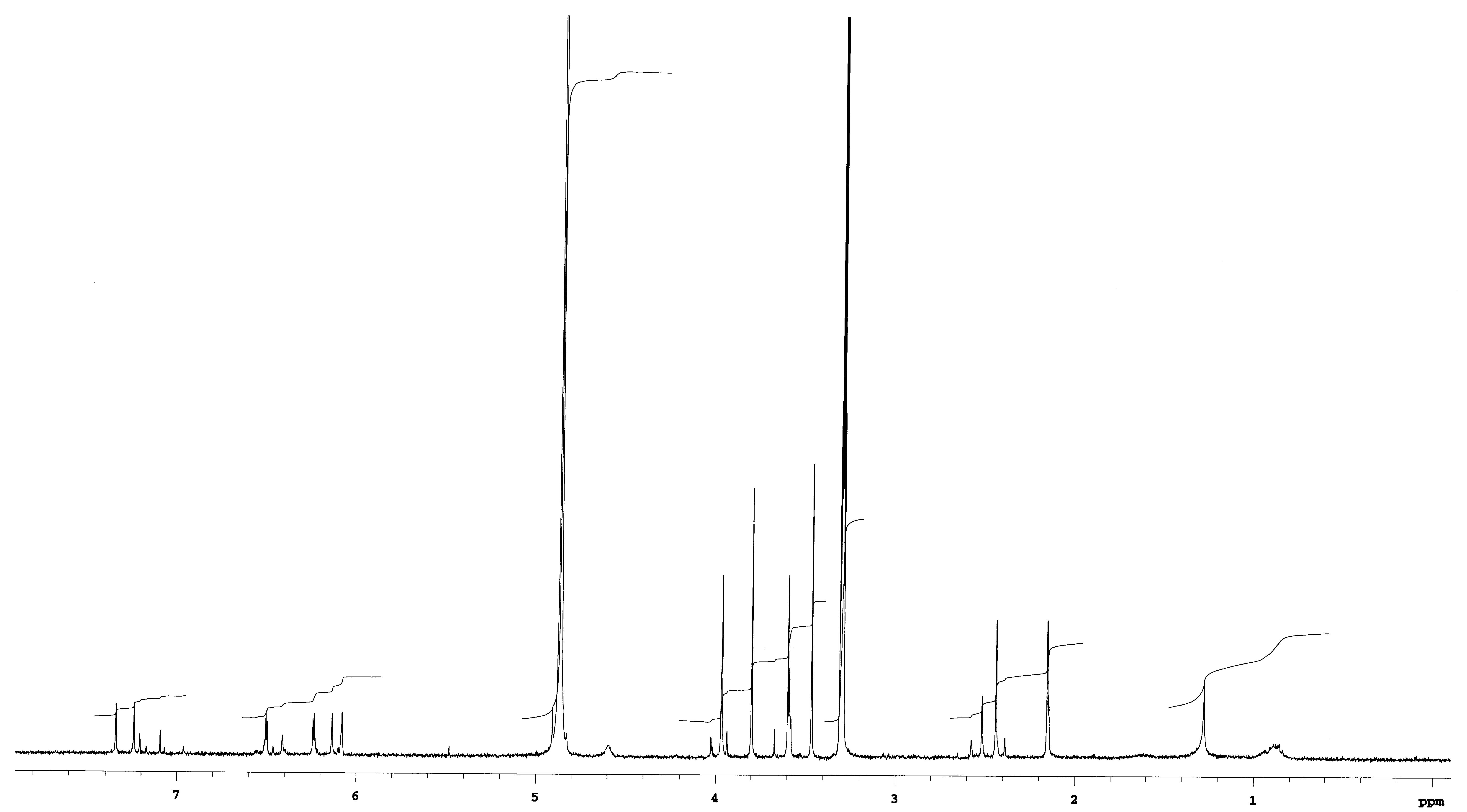


**Chart 36:** ^1^H NMR spectrum (CD_3_OD, 300 MHz) of Aurasperone A (**6**)
