# Supplementary material for: Seven naphtho-γ-pyrones from the marine-derived fungus Alternaria alternata: structure elucidation and biological properties
Source: Org Med Chem Lett. 2012 Feb 29;2:6. doi: 10.1186/2191-2858-2-6 (PMC3350997; doi:10.1186/2191-2858-2-6)
Supplement: Additional file 6 — Spectral data of Aurasperone B (7). Three charts (chart 37-39) containing the mass (HRESI, EI MS) and NMR (1HNMR) spectral data of Aurasperone B (7) [file 2191-2858-2-6-S6.DOC]

**6. Additional file 6**

**Title:** Spectral data of Aurasperone B (**7**)

**Description:** Three charts (chart 37-39) containing the mass (HRESI, EI MS) and NMR (^1^HNMR) spectral data of Aurasperone B (**7**)


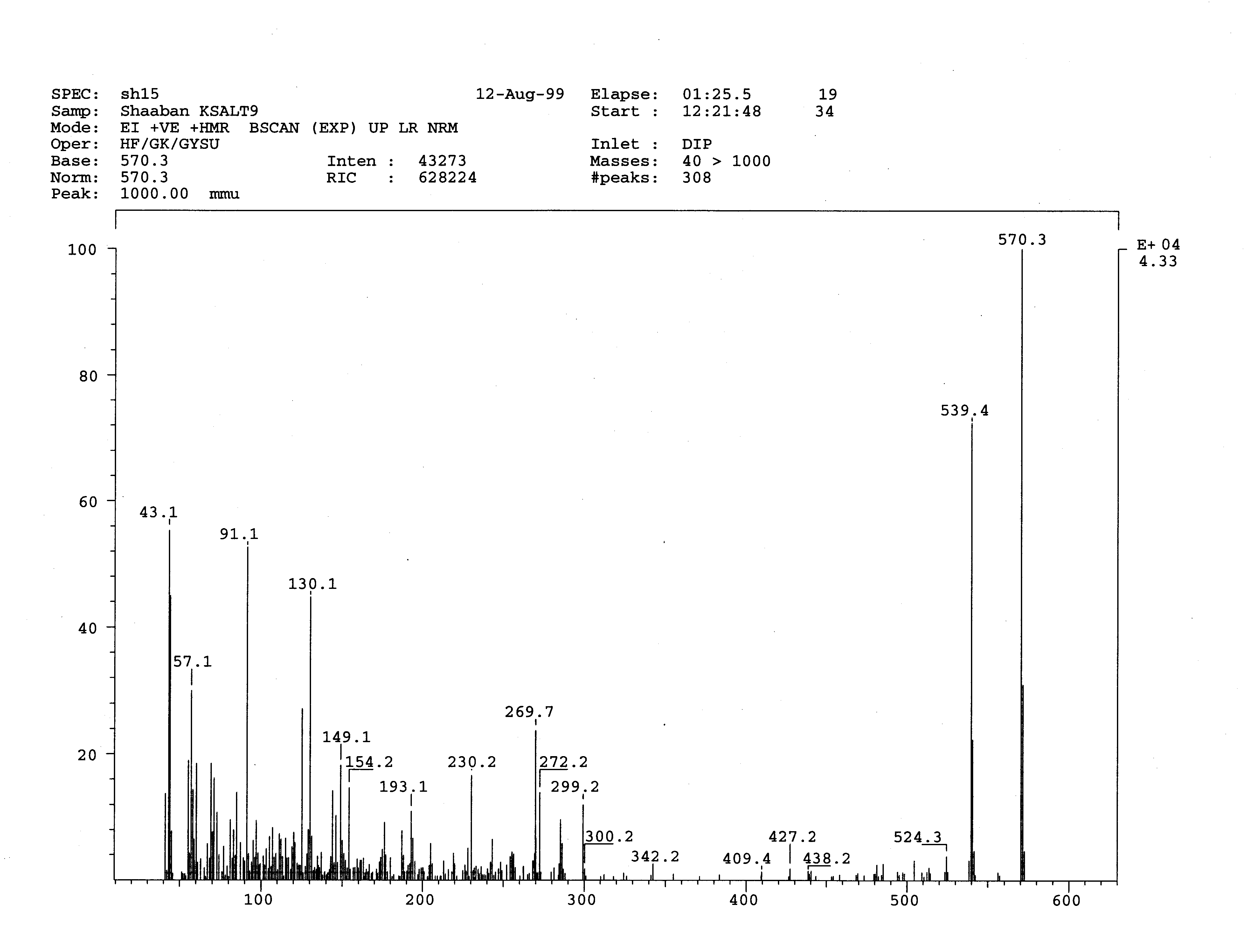


**Chart 37:** EI-MS spectrum of Aurasperone B (**7**)


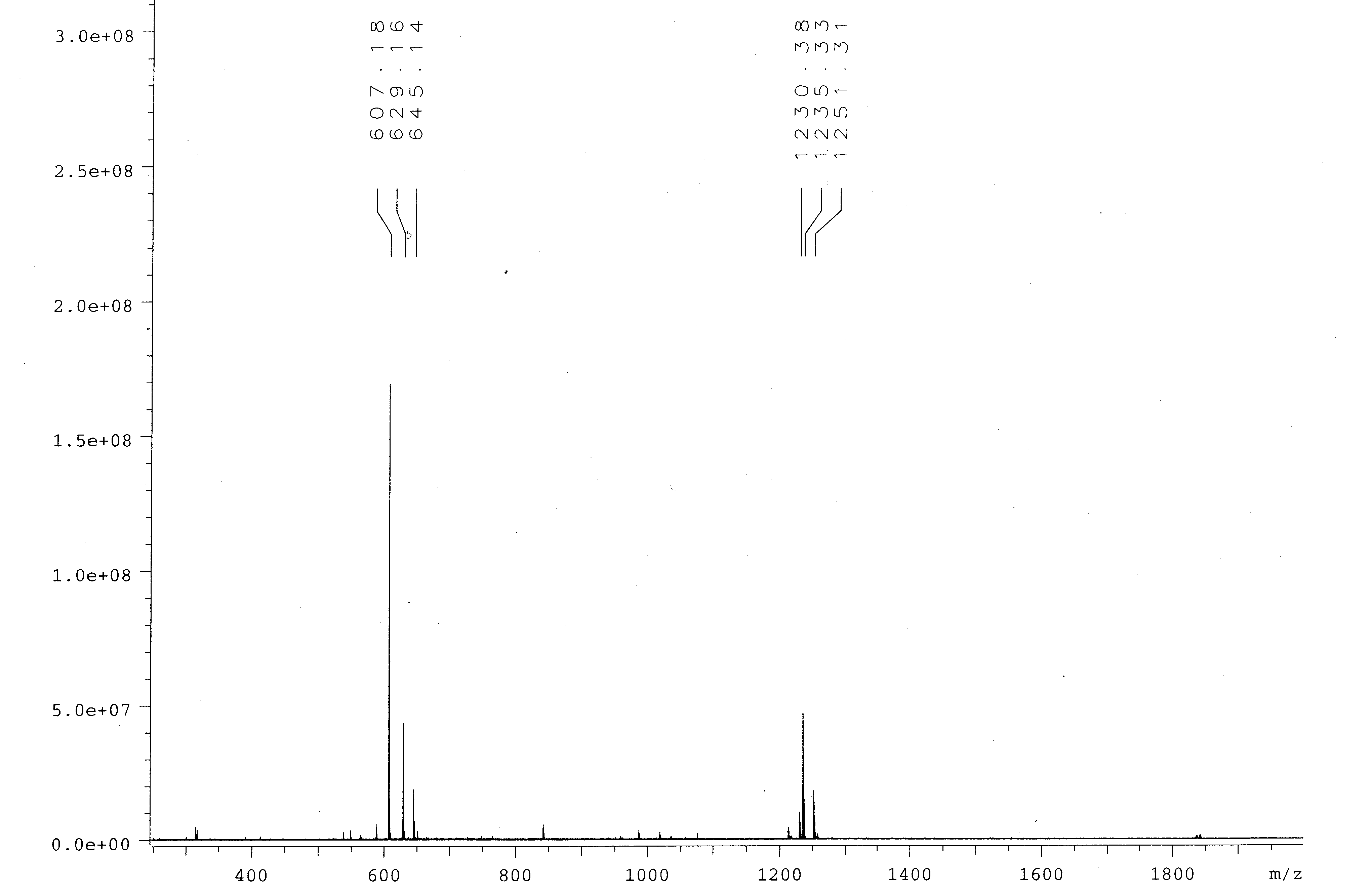


**Chart 38**: (+)-HRESI-MS spectrum of Aurasperone B (**7**)


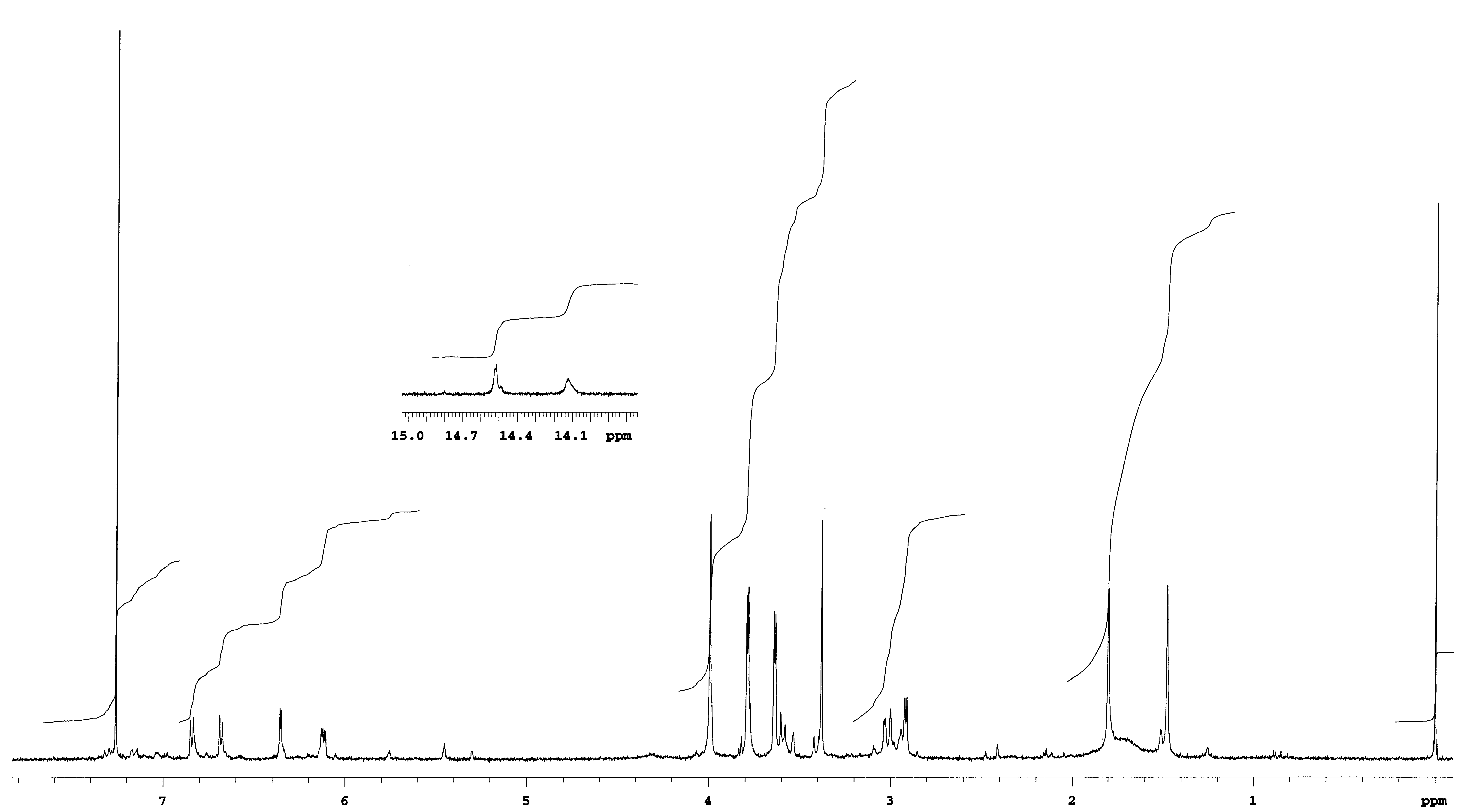


**Chart 39:** ^1^H NMR spectrum (CDCl_3_, 300 MHz) of Aurasperone B (**7**)
