# Supplementary material for: Seven naphtho-γ-pyrones from the marine-derived fungus Alternaria alternata: structure elucidation and biological properties
Source: Org Med Chem Lett. 2012 Feb 29;2:6. doi: 10.1186/2191-2858-2-6 (PMC3350997; doi:10.1186/2191-2858-2-6)
Supplement: Additional file 7 — Spectral data of Aurasperone C (8). Four charts (chart 40-43) containing the mass (ESI, HRESI MS) and NMR (1HNMR) spectral data of Aurasperone C (8) [file 2191-2858-2-6-S7.DOC]

**7. Additional file 7**

**Title:** Spectral data of Aurasperone C (**8**)

**Description:** Four charts (chart 40-43) containing the mass (ESI, HRESI MS) and NMR (^1^HNMR) spectral data of Aurasperone C (**8**)

**
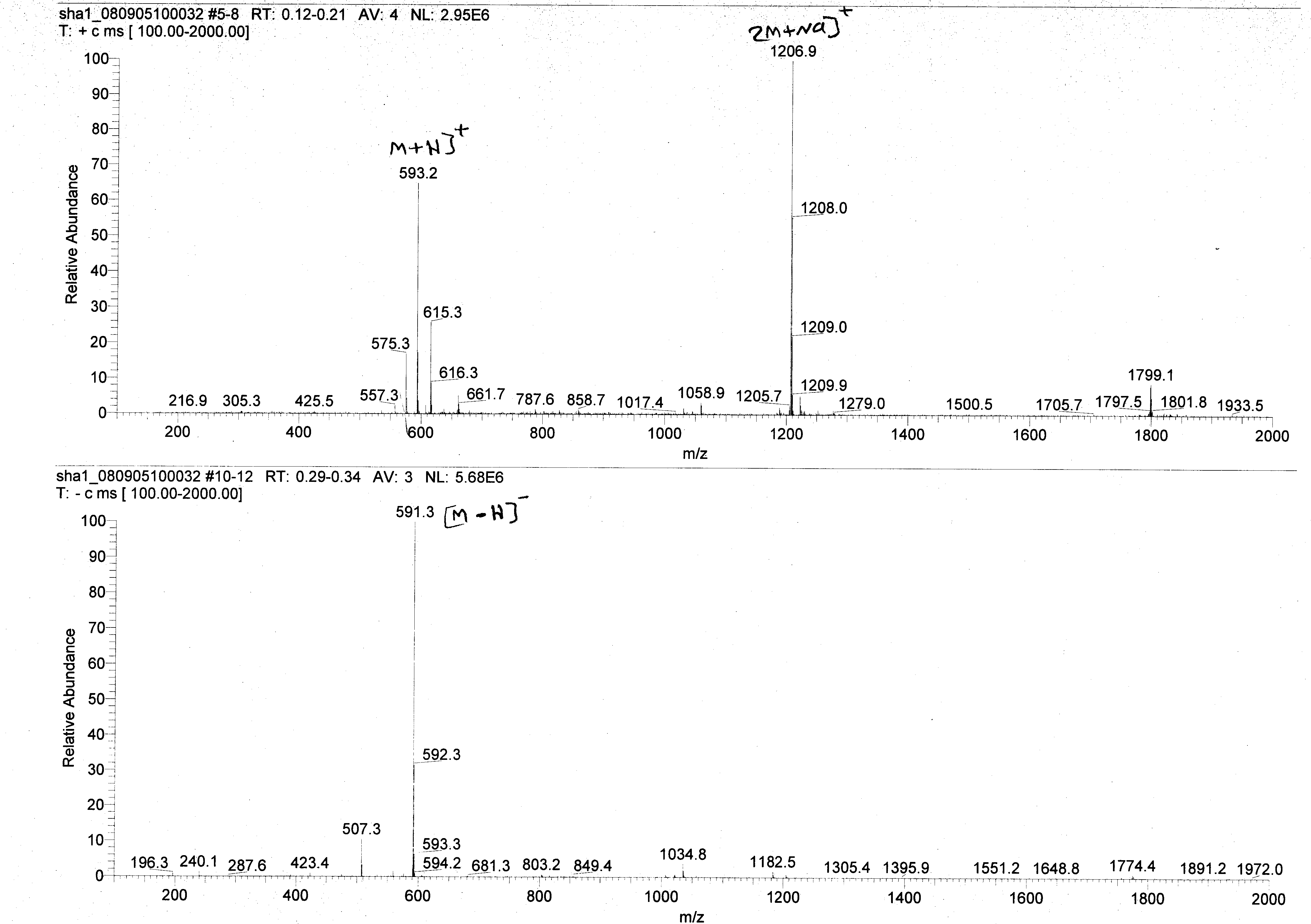
**

**Chart 40:** (+)-ESI-MS and (-)-ESI-MS spectra of Aurasperone C (**8**)


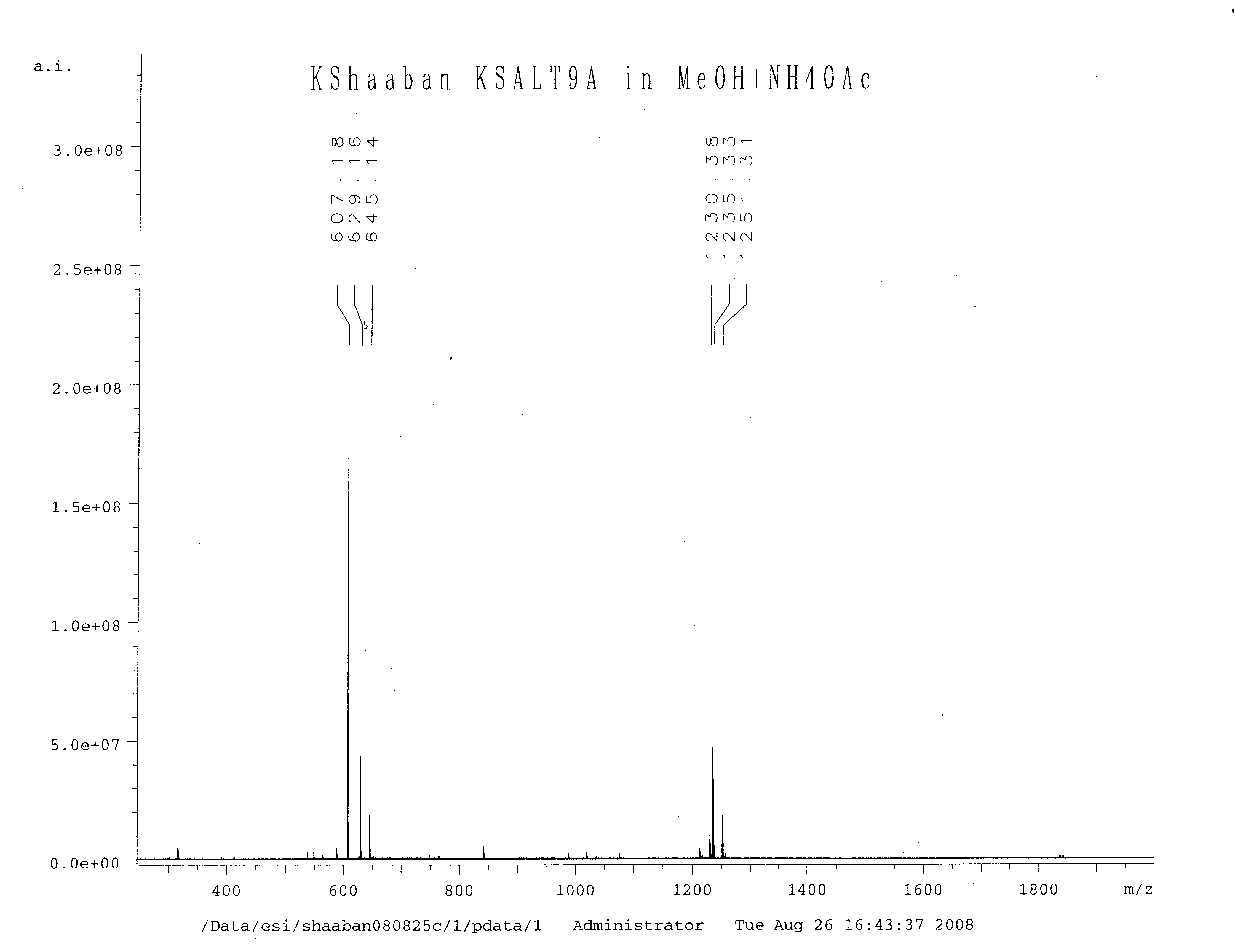


**Chart 41:** (+)-HRESI-MS spectrum of Aurasperone C (**8**)


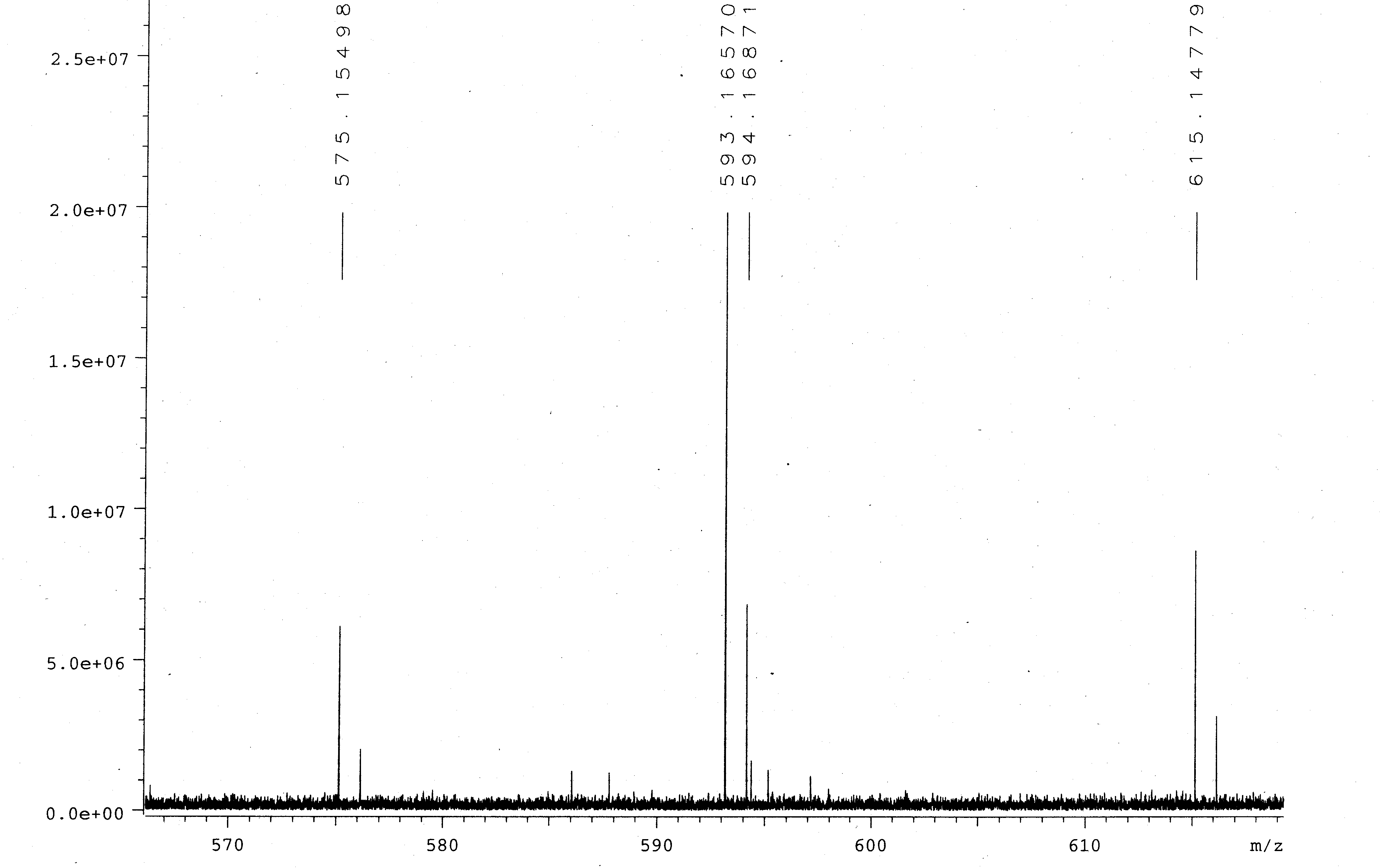


**Chart 42:** (+)-HRESI-MS expansion spectrum of Aurasperone C (**8**)


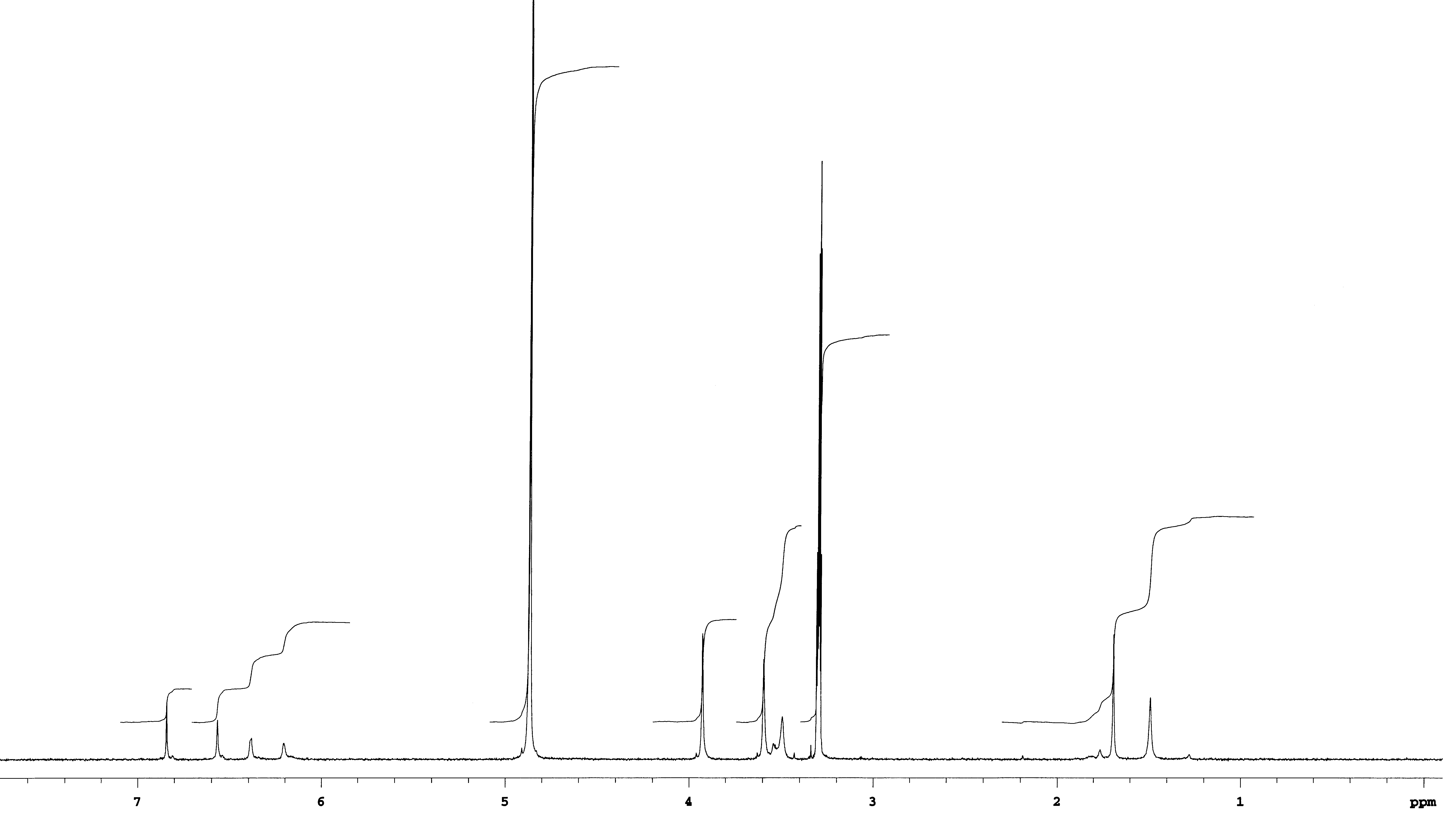


**Chart 43:** ^1^H NMR spectrum (CD_3_OD, 300 MHz) of Aurasperone C (**8**)
