# Supplementary material for: Seven naphtho-γ-pyrones from the marine-derived fungus Alternaria alternata: structure elucidation and biological properties
Source: Org Med Chem Lett. 2012 Feb 29;2:6. doi: 10.1186/2191-2858-2-6 (PMC3350997; doi:10.1186/2191-2858-2-6)
Supplement: Additional file 8 — Spectral data of Aurasperone F (9). Three charts (chart 44-46) containing the mass (ESI, EI MS) and NMR (1HNMR) spectral data of Aurasperone F (9) [file 2191-2858-2-6-S8.DOC]

**8. Additional file 8**

**Title:** Spectral data of Aurasperone F (**9**)

**Description:** Three charts (chart 44-46) containing the mass (ESI, EI MS) and NMR (^1^HNMR) spectral data of Aurasperone F (**9**)


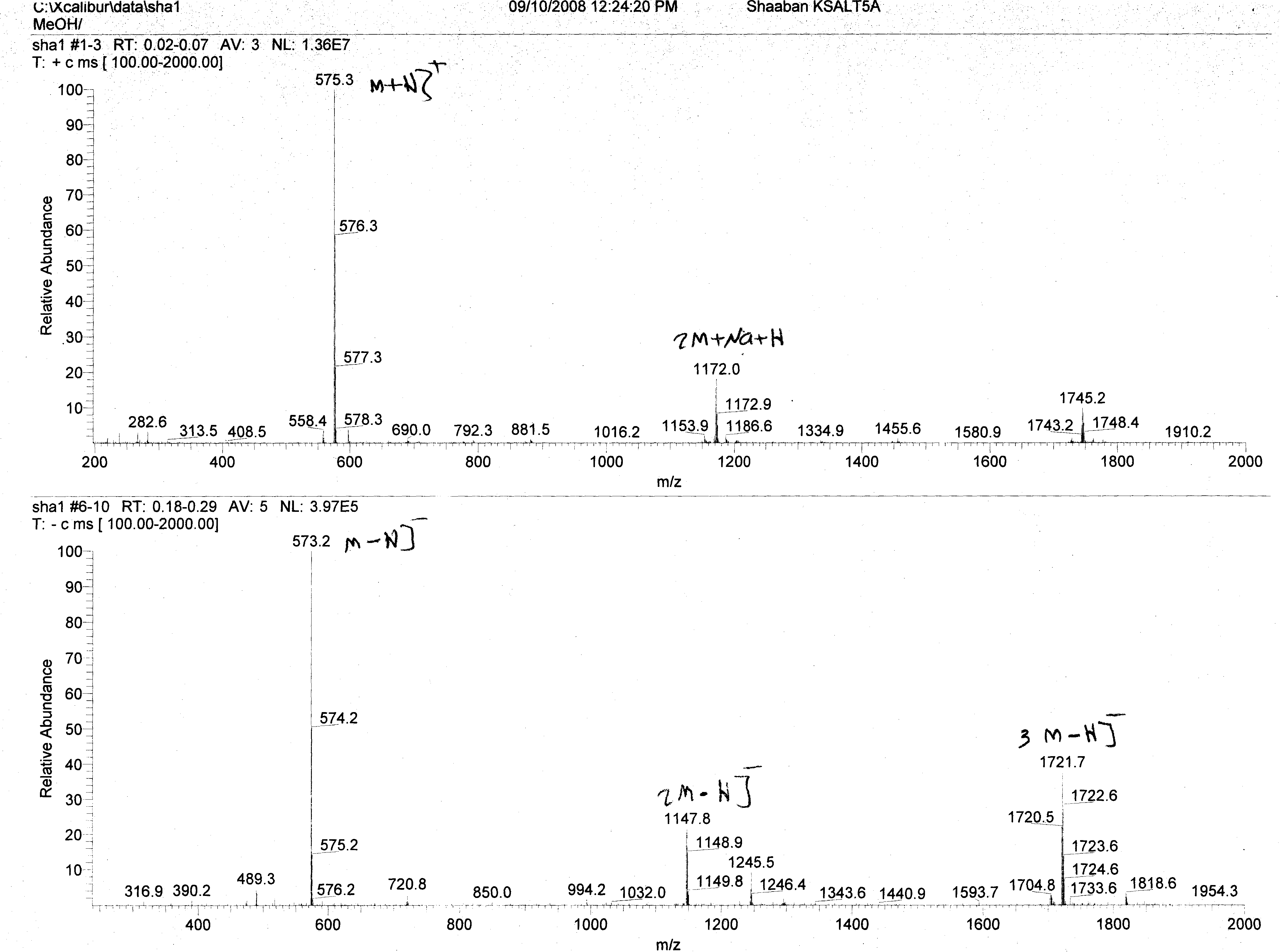


**Chart 44:** (+)-ESI MS and (-)-ESIMS spectra of Aurasperone F (**9**)


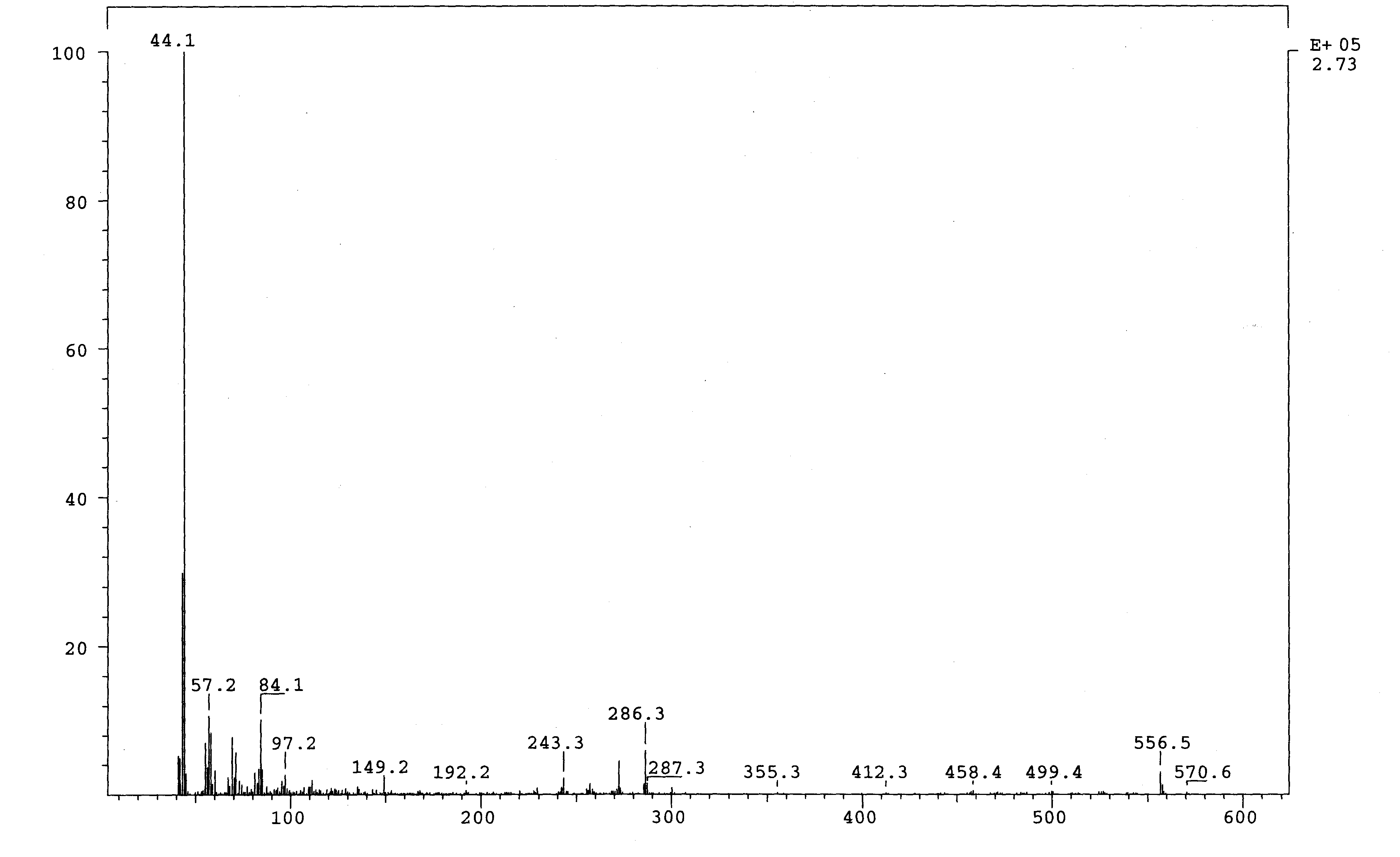


**Chart 45:** EI-MS spectrum of Aurasperone F (**9**)


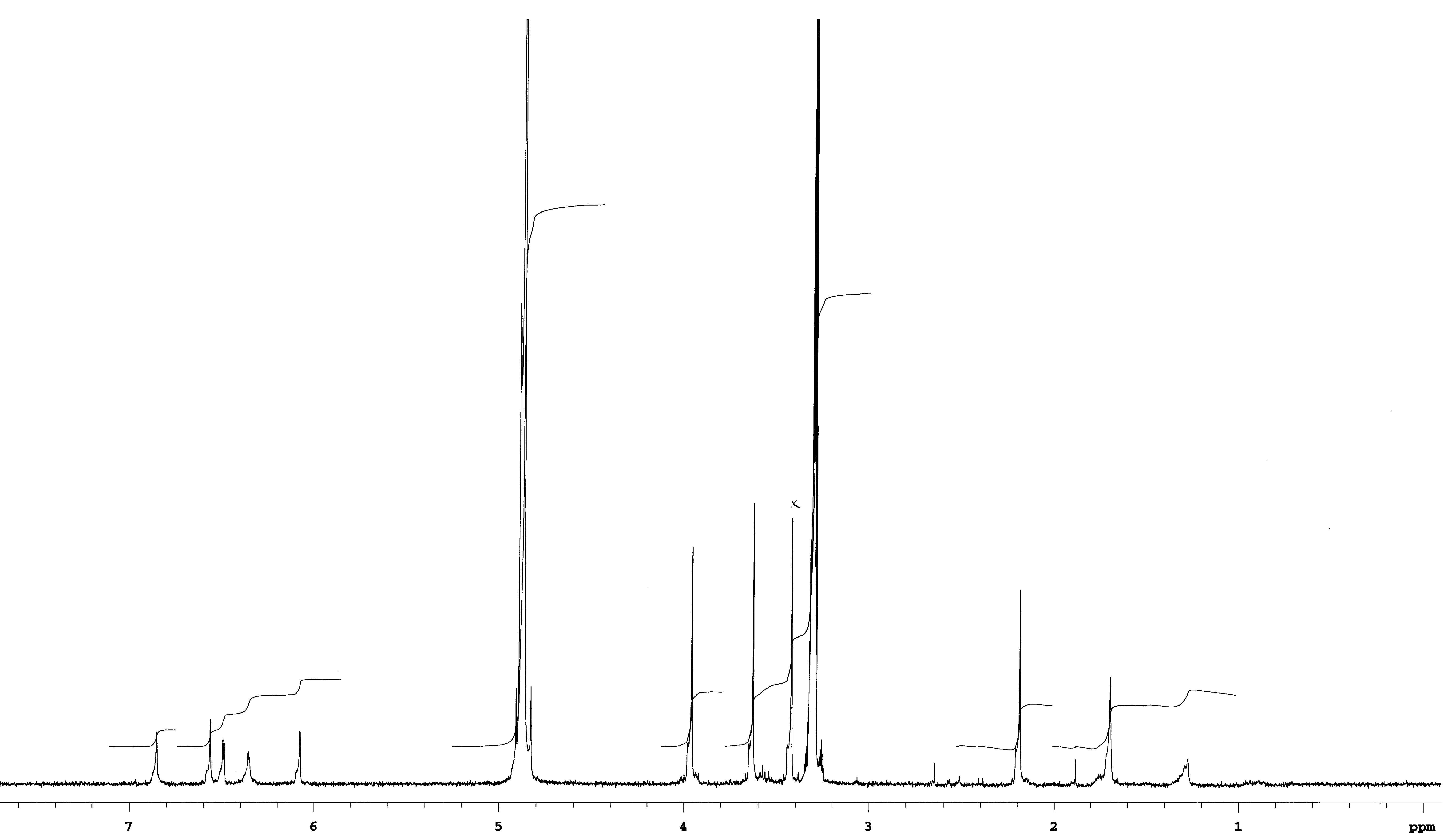


**Chart 46:** ^1^H NMR spectrum (CD_3_OD, 300 MHz) of Aurasperone F (**9**)
