# Supplementary material for: Seven naphtho-γ-pyrones from the marine-derived fungus Alternaria alternata: structure elucidation and biological properties
Source: Org Med Chem Lett. 2012 Feb 29;2:6. doi: 10.1186/2191-2858-2-6 (PMC3350997; doi:10.1186/2191-2858-2-6)
Supplement: Additional file 9 — CD Spectra of Aurasperones A-C (6-8). Three charts (chart 47-49) containing the CD spectral data of Aurasperones A-C (6-8). [file 2191-2858-2-6-S9.DOC]

**9. Additional file 9**

**Title:** CD Spectra of Aurasperones A-C (**6-8**)

**Description:** Three charts (chart 47-49) containing the CD spectral data of Aurasperones A-C (**6-8**)

**
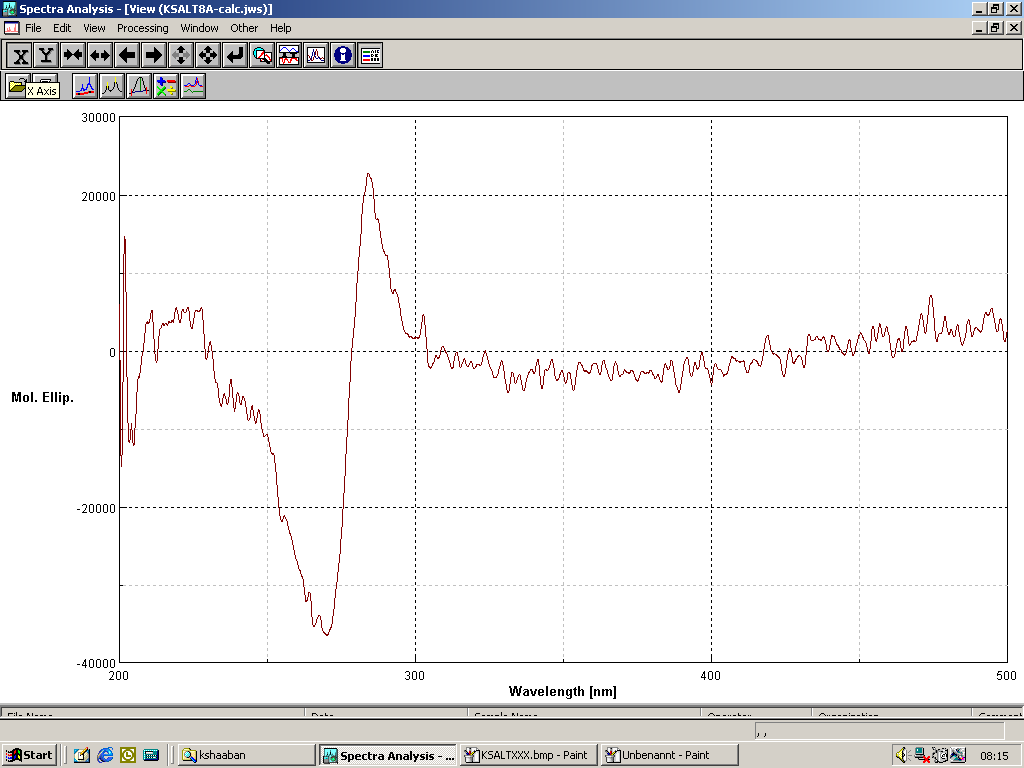
**

**Chart 47:** CD spectrum of Aurasperone A (**6**)

**
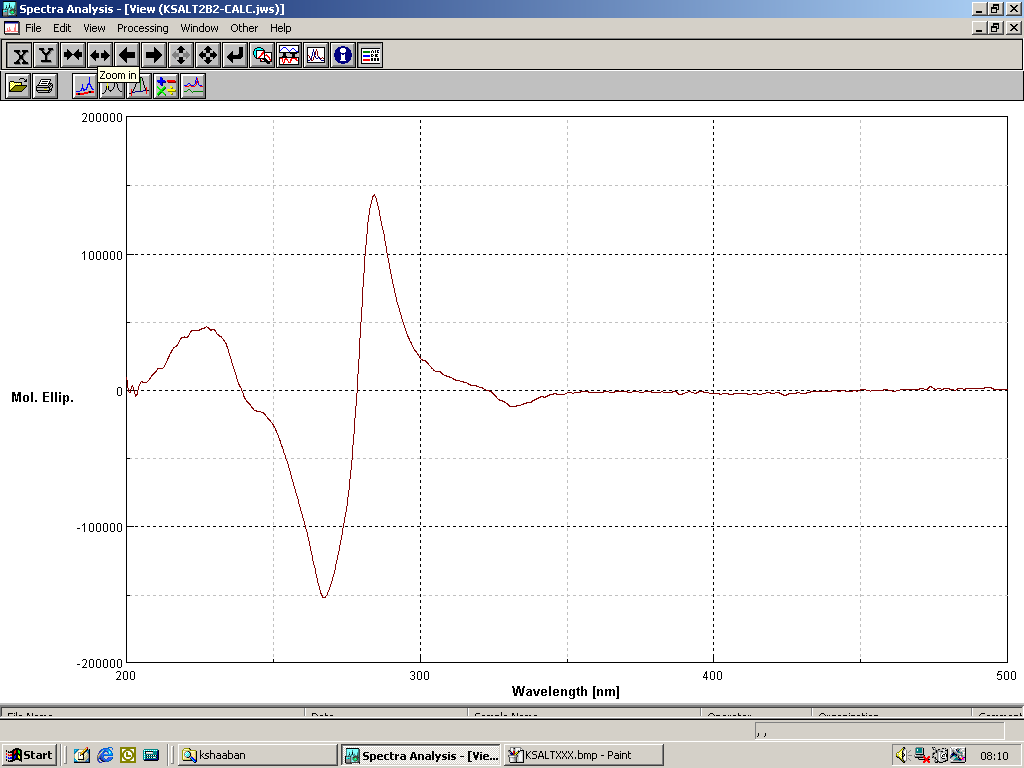
**

**Chart 48:** CD spectrum of Aurasperone B (**7**)

**
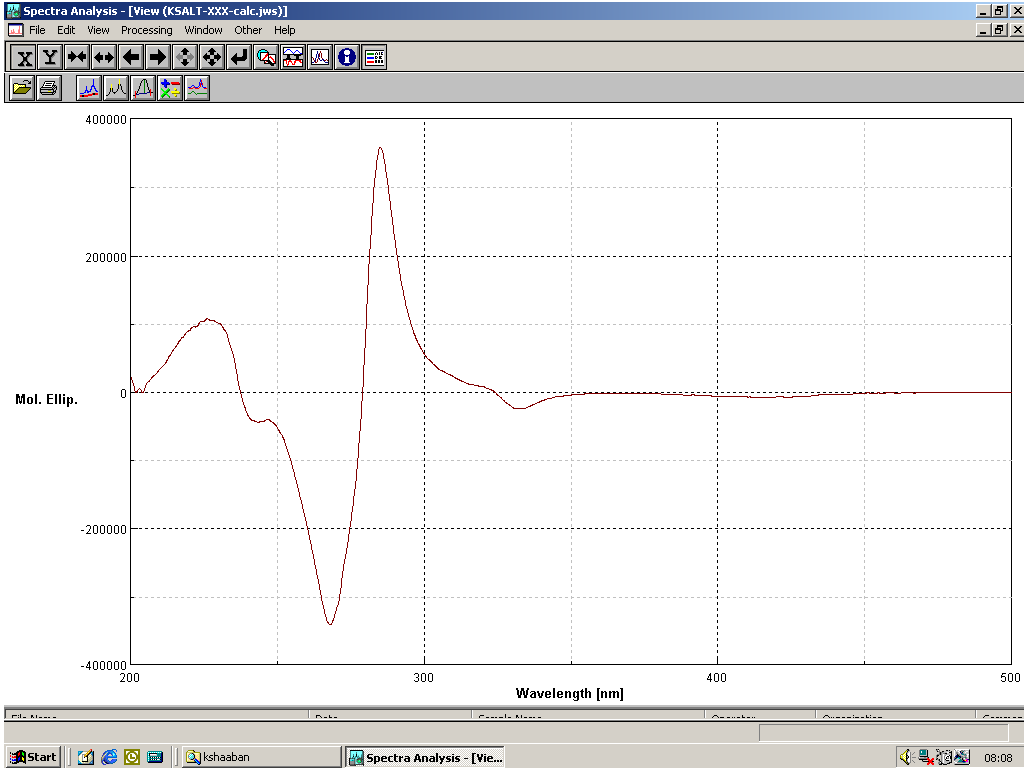
**

**Chart 49:** CD spectrum of Aurasperone C (**8**)
